# Socioeconomic inequalities in exposure to neighbourhood environments for physical activity: a systematic review

**DOI:** 10.1186/s12966-026-01912-1

**Published:** 2026-04-09

**Authors:** Jet D. S. van de Geest, Valeria-C. Cuenca, Linda J. Schoonmade, Luis Cereijo, Diana J. Mora, Paul Meijer, Jeroen Lakerveld

**Affiliations:** 1 Department of Epidemiology and Data Science, Amsterdam UMC Location Vrije Universiteit Amsterdam, De Boelelaan 1117, Amsterdam, 1081HV the Netherlands; 2 Amsterdam Public Health Research Institute, Amsterdam, 1105 AZ the Netherlands; 3 Upstream Team, https://www.upstreamteam.nl/; 4 Public Health and Epidemiology Research Group, School of Medicine and Health Sciences, Universidad de Alcalá, Alcalá de Henares, Madrid, Spain; 5 University Library, Vrije Universiteit Amsterdam, Amsterdam, The Netherlands

**Keywords:** Physical activity environment, Socioeconomic inequalities, Socioeconomic position, Health inequalities, Exposome, Spatial epidemiology

## Abstract

**Background:**

Insufficient physical activity (PA) is a major behavioural determinant of obesity and other noncommunicable diseases. Although the health benefits of PA are well established, many populations, particularly socioeconomically disadvantaged groups, remain insufficiently active. Neighbourhood characteristics play an important role in shaping population physical activity (PA) levels, for example through walkable streets, bikeable infrastructures, playgrounds, green- and blue spaces, and sport facilities. However, PA-supportive environments may be unequally distributed across SEP groups. This systematic review provides a comprehensive synthesis of evidence on socioeconomic inequalities in neighbourhood environments that promote PA across different life stages in high-income countries.

**Methods:**

In February 2024, we systematically searched Medline, Embase, Web of Science Core Collection, and Scopus. Eligible studies were quantitative primary research examining objective measures of neighbourhood PA-promoting environments alongside at least one individual- or area-level socioeconomic indicator. Titles and abstracts were screened with ASReview, and full texts were screened manually in duplicate. Data were extracted with a predefined form, and risk of bias was assessed with an adapted version of the AXIS tool. We synthesised our findings narratively and by reported proportions of associations indicating disadvantaged exposure, advantaged exposure, or no statistically significant difference for people with lower SEP.

**Results:**

A total of 250 studies were included. Overall, people with lower SEP were more likely to live in areas with (components of) walkable and bikeable infrastructure, and to have playgrounds nearby, but less likely to have access to formal sports facilities. For other PA-promoting environments, findings were largely null. Patterns varied across age groups, study regions, statistical approaches, and SEP indicators.

**Conclusions:**

Populations with lower SEP were more exposed to walkability components, bikeability, and playgrounds, but less exposed to sports facilities. For most other environments, no clear differences were found. Socioeconomic inequalities in exposure to PA environments are nuanced rather than uniform. This complexity highlights the need to consider multiple environmental features together and to tailor equity-focused interventions to local contexts.

**Supplementary Information:**

The online version contains supplementary material available at 10.1186/s12966-026-01912-1.

## Background

The health benefits of physical activity (PA) are well established. It contributes to a reduced risk of chronic diseases and all-cause mortality [1], and supports improvements in metabolic health, body composition, quality of life, and overall wellbeing [2, 3]. Conversely, insufficient PA is a major behavioural determinant of obesity [4] and other noncommunicable diseases [5]. Globally, more than one in four adults do not meet the World Health Organization’s PA recommendations [5]. However, insufficient PA is not evenly distributed across the population; socioeconomic differences in PA behaviours are well-documented. Reviews consistently show that adults with a higher SEP engage more in leisure-time PA, whereas occupational PA is more common among people with a lower SEP [6, 7]. Evidence for differences in active transport is mixed, with no clear socioeconomic pattern [7]. Among children and adolescents, youth with a lower SEP generally seem to be less physically active [8] and less likely to participate in organised PA, although findings for daily PA intensities (e.g., moderate-to-vigorous PA, total PA) are heterogeneous, with many studies reporting no association [9]. Some evidence shows that socioeconomic disadvantage in early life can contribute to lower PA levels later in adulthood, partly through its influence on socioeconomic circumstances across the life course [10].

Social inequalities in PA behaviour are shaped not only by individual factors but also by environmental conditions. The neighbourhood built environment plays an important role in facilitating opportunities to be physically active. Neighbourhood availability of features such as walkable infrastructure, mixed land use, green spaces, public transport, and playgrounds promotes active lifestyles, contributes to healthier weight outcomes, and lowers the risk of diabetes and cardiovascular diseases [11–20]. Recognising the importance of environmental factors on people’s health, the World Health Organization’s Global Action Plan on Physical Activity (2018–2030) [21] emphasises the need for a systems-based approach, combining upstream policy actions that address social, cultural, economic, and environmental determinants with downstream educational and informational strategies. The plan advocates for equitable access to safe and supportive environments that enable all individuals to engage in regular physical activity.

However, the existing evidence suggests that access to PA-supportive environments is not equally distributed across socioeconomic groups. Previous reviews examining socioeconomic inequalities in exposure to PA-supportive environments highlight that the evidence base remains highly inconsistent [22, 23]. For walkable infrastructure, both Jacobs et al. and Høyer-Kruse et al. report mixed findings, with no clear advantage for either higher- or lower-SEP populations [22, 23]. For bikeable infrastructure, both reviews suggest that areas with lower SEP tend to have fewer cycling facilities [22, 23]. Jacobs et al. also found that areas with lower SEP had slightly more favourable exposure to recreational facilities, whereas green space exposure tended to be less favourable in these areas [23]. Taken together, the two reviews show that socioeconomic differences in PA-supportive environments may not follow a consistent pattern.

To build on and update the existing evidence, the current systematic review aims to expand existing knowledge, incorporating both individual- and area-level SEP indicators and including more recent studies. We examine socioeconomic inequalities in exposure to neighbourhood PA-promoting environments across different life stages in high-income countries and include a broad range of environmental PA-promoting resources. We also explored whether broad study-level characteristics might contribute to variation in findings.

## Methods

This study is part of the Obesity: Biological, socioCultural, and environmental risk Trajectories (OBCT) project [24]. It is part of a two-part analysis of socioeconomic inequalities in exposure to obesogenic environment factors. The other review focuses on the food environment and is submitted elsewhere. Both reviews were conducted simultaneously using an identical protocol and methodology, which are described below.

The protocol for this systematic review was pre-registered in the International Prospective Register of Systematic Reviews (PROSPERO) (ID number CRD42024503986). The reporting is conducted according to the Preferred Reporting Items for Systematic Reviews and Meta-Analysis (PRISMA) guideline for systematic reviews [25, 26]. The PRISMA checklist is included in Supplementary File 1.

### Search strategy

A comprehensive literature search was performed in the bibliographic databases Medline/Ovid, Embase/Elsevier, Web of Science Core Collection/Clarivate and Scopus/Elsevier. The search was conducted in collaboration with a medical librarian (LS) and included studies published between January 1 st, 2000, and February 26th, 2024. The year 2000 was selected as the cut-off because it corresponds to the approximate period identified in the literature when interest in the measurement of PA environments began to emerge [27]. Search terms included controlled terms (MeSH in Medline and Emtree in Embase) as well as free text terms. Synonyms and closely related terms were used for (indicators of) individual- and neighbourhood level SEP (e.g. household income, neighbourhood-average education), inequality (e.g. differences, disparities), built environment PA resources(e.g. green spaces, walkability) and exposure measures (e.g. availability, proximity). The complete search strategy is reported in Supplementary File 2.

### Inclusion and exclusion criteria

Studies were eligible for inclusion if they were primary research articles published in English that examined objectively measured PA environment exposures in relation to at least one individual- or area-level SEP indicator, using quantitative data. Objectively measured exposures were defined as environmental characteristics assessed using non-self-reported data sources, including for example GIS-based indicators, audit-based assessments, and public facility registries.

No age restrictions were applied. To be considered quantitative, studies had to present numerical values (e.g., counts, proportions, statistical associations); studies presenting only graphical representations without precise numerical data were excluded. Additional exclusion criteria were: non-primary research (e.g., commentaries, reviews), studies focused solely on perceived exposures, studies examining air pollution, studies on activity space, and studies conducted in low- and middle-income countries (as classified by the World Bank) [3].

### Study selection and data extraction

Duplicate records were removed by LS. Title and abstract screening was primarily performed by one reviewer (JG), with aid of the artificial intelligence screening tool ASReview version 1.5 [28]. ASReview uses active learning techniques to continuously restack the order of articles to be screened according to potential relevance for inclusion. The ASReview model was initially trained using a random set of 1% of the total number of records. To ensure consistency and reliability during the screening process, a random sample of 150 papers were independently screened by another reviewer (JL, also using ASReview), and discrepancies were resolved through discussion. After 280 articles were subsequently classified as irrelevant and the set of articles eligible for both full-text screenings appeared comprehensive, the title and abstract screening was stopped in agreement between PM, JL, and JG.

Full-text screening was performed in duplicate by three independent reviewers (VC, DM, and JG), using Covidence. Any discrepancies were resolved through discussion. Data extraction was independently carried out by VC, JG, and a research assistant (LvS), using a predefined data extraction form created by JG (Supplementary File 3). Key data included SEP indicators (e.g. income, education, occupation/employment, or composite SEP scores), exposure measures, setting (urban/rural), unit of analysis, sample size, and analytical methods. Predetermined environmental PA resources were: greenspace (including greenness indices), blue and public open spaces, sports facilities, walkability (components and indices), bikeability, public transport stops, and playgrounds [29]. Combined scores (e.g. walking and cycling paths) or indices on the overall PA environment were classified as ‘other’ PA resource.

### Quality assessment

To evaluate the study quality of the included primary studies, VC, JG, and LvS independently applied a shortened version of the Appraisal Tool for Cross-sectional Studies [30], adapted for both individual-level and ecological studies. Ten percent of the assessments were conducted in duplicate to ensure consistency. The adapted tool focused on transparency in reporting, including variable definition, reproducibility, limitations, and treatment of missing data, and is included in Supplementary File 4. The AXIS tool does not provide guidelines on the overall grading of each study. Consequently, a scoring system was devised whereby studies were assigned a quality rating based on the percentage of total points they received. Studies were categorised as poor (< 50%), fair (50–75%), or good quality (> 75%) based on the percentage of total criteria met.

### Synthesis of results

The findings were synthesised narratively, stratified by PA resource. Within each resource, we analysed results across all SEP operationalisations together, as well as stratified by individual SEP proxy (i.e., income, education, occupation, ‘other’, or composite SEP measures). This allowed us to better understand the variations across different socioeconomic contexts and PA resources. In our synthesis we distinguished between adjusted regression analyses and ‘non-regression’ methods (e.g., ANOVA, Chi-squared tests, bivariate regressions). Adjusted regression-based associations were investigated separately and deemed more important because of their ability to account for potential confounding. In this context, confounders were considered variables that may influence both SEP and exposure to PA-supportive environments. These include neighbourhood sociodemographic characteristics (e.g., proportion of children and elderly, ethnic composition, neighbourhood-level income) and built-environment features (e.g., population density, proportion of built-up area), which together can shape both residential SEP and the distribution of PA resources. In individual-level analyses, commonly adjusted variables such as age, sex, household composition, and additional SEP indicators may also act as confounders because they influence both an individual’s SEP and their likelihood of living in particular neighbourhoods. Bivariate (unadjusted) regressions were categorised under ‘non-regression’ methods due to their primarily descriptive nature. They were only included in the review when no adjusted regression results were reported in the papers. We reported the proportions of associations indicating an advantage, disadvantage, or no statistically significant difference for the lowest SEP group compared to the highest, irrespective of the number of SEP categories used. Additionally, we examined patterns emerging from descriptive statistics.

### Additional stratified analyses

To provide further nuance beyond the main descriptive synthesis, we conducted several stratified analyses to uncover potential hidden patterns. Specifically, we stratified associations by geographical context (USA vs. non-USA studies), level of analysis (individual-level vs. ecological-level studies), and age group (children vs. adults and older adults). We examined these subgroups to explore whether broad study-level differences across the included studies contributed to variation in findings. The decision to include USA-based stratification was made post hoc, based on the observation that nearly half of all included studies were conducted in the USA. For age-stratified analyses, we only included associations that explicitly focused on children or adults/older adults. Associations referring to the total population or to combined age groups were excluded from the age-stratified analyses to allow for clearer group comparisons. Older adults were grouped with adults due to the limited number of associations focused exclusively on this age group. In contrast to the main analysis, the stratified analyses combined all SEP indicators and statistical methods to facilitate the identification of broad patterns across strata.

## Results

**Fig. 1:**
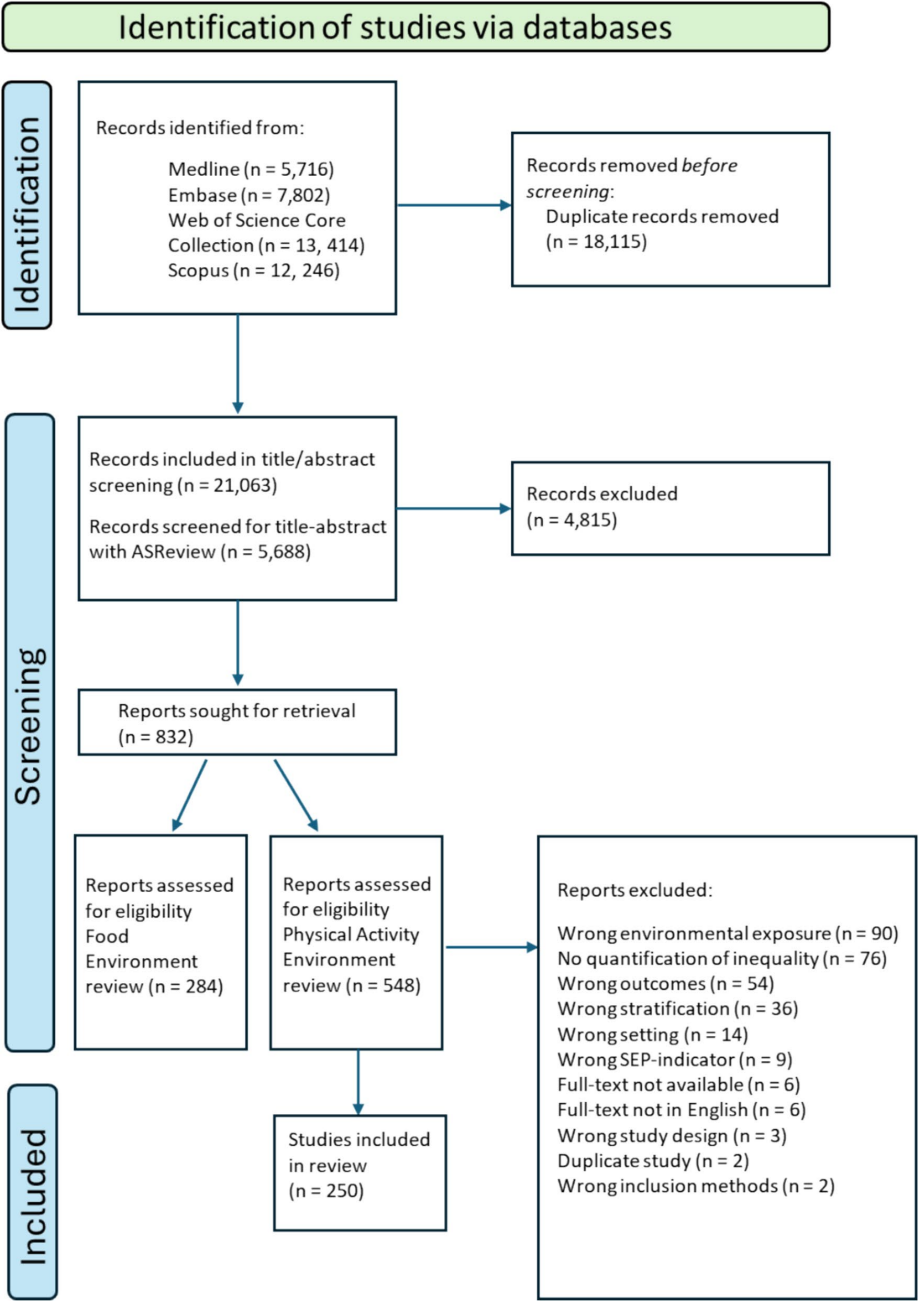
Flow diagram of the search and selection procedure of studies Note: the identification and screening phase was combined for the current review as well as for another review on SEP inequalities in neighbourhood food environments

The literature search yielded 39,178 records. After deduplication of overlapping entries across databases, a total of 21,063 unique references remained. An overview of the selection process is provided in Fig. 1. Reasons for exclusion included: wrong environmental exposure (i.e., studies that did not examine a relevant PA-supportive environmental feature), no quantification of inequality (i.e., only graphical/qualitative presentation), wrong outcomes (i.e., studies in which the exposure was a PA facilitator or SEP, but the outcome was unrelated to our review question, such as disease outcomes), wrong stratification (i.e., no relevant stratification to analyse SEP inequalities), wrong setting (i.e., studies conducted outside Europe), wrong SEP indicator (e.g. SEP operationalised or combined with ethnicity), full-text not available, full-text not in English, wrong study design, duplicate study, or wrong inclusion methods (i.e., participants selected to represent specific demographic groups).

### Study characteristics

A total of 250 studies were included in the synthesis. The most frequently examined environmental PA facilitator was green, blue, and public open space, assessed in 117 studies (46.8%). Income was the most commonly used indicator of SEP, featuring in 158 studies (63.2%). Regression analyses were performed in 87 studies (34.8%). Other statistical procedures that occurred most often were bivariate correlations (in 55 studies; 22%), ANOVA (in 20 studies; 8%), and t-tests (in 19 studies; 7.6%). Almost half of the studies (41.6%) were conducted in the USA. The majority of studies adopted an ecological scale of analysis. Regarding age groups under study, 24% focused only on adults or older adults, and 13.6% on children. The remaining studies either targeted the general population or did not specify an age group. An overview of all included studies is provided in Table 1. To aid interpretation, Supplementary File 5 provides a visual illustration of commonly used units and buffer sizes, offering a general sense of their relative scale. More details on operationalisations of SEP measures and PA resources, as well as analytical approaches and outcome of study quality assessments is available in the extended descriptive table (Supplementary File 6).

**Table 1:** Basic information on included studies

Author, year of publication	Country: region(s) (setting)	SEP-indicator(s) studied	Environmental PA resource(s) studied	Geographic unit(s) of analysis (n)
Aamodt et al., 2023 [31]	Finland: Espoo; Norway: Stavanger; Sweden: Täby (urban)	Income and education	Green, blue, public open spaces	Neighbourhood (7, 9, 38)
Abercrombie et al., 2008 [32]	USA: Baltimore-Washington DC (urban)	Income	Green, blue, public open spaces and sports facilities	Block group (833) and census tract (833)
Agay-Shay et al., 2019 [33]	Israel: Tel Aviv (urban)	Education and SEP index	Greenness index	Individual/household (300 m buffer around home) (73,221)
Akaraci et al., 2021 [34]	Australia: Sydney (urban)	SEP index	Green, blue, public open spaces	Statistical Area Level 2 (approx. 200)
Alderton et al., 2022 [34]	Australia: Adelaide (urban)	Education and SEP index	Playgrounds	Individual/household (400 and 800 m buffer around home) (199,200)
Apparicio et al., 2016 [35]	Canada: Island of Montreal (urban)	Income	Green, blue, public open spaces	City blocks (10,210)
Astell-Burt et al., 2014 [36]	Australia: Sydney, Melbourne, Brisbane, Perth, Adelaide (urban)	Income	Green, blue, public open spaces	Statistical Area Level 1 (28,626)
Aznarez et al., 2023 [37]	Spain: Vitoria-Gasteiz (urban)	Education	Green, blue, public open spaces	Neighbourhood (28)
Badland et al., 2010 [38]	New Zealand: Waitakere City (urban)	SEP index	Sports facilities	Clusters of 5 contiguous mesh-blocks (geographic units of approximately 100 households) (69)
Banzhaf et al., 2017 [39]	Chile: Cerro Navia, La Florida, and Vitacura (urban)	Income	Green, blue, public open spaces	Municipality (3)
Bao et al., 2023 [40]	UK: Greater London, Northeast England, Northwest England, Yorkshire and the Humber, West Midlands, East Midlands, East England, Southwest England, and Southeast England (urban)	Education and housing	Green, blue, public open spaces	Lower Layer Super Output Areas (32,844)
Baró et al., 2021 [41]	Spain: Barcelona (urban)	Income and education	Green, blue, public open spaces and playgrounds	School (324)
Barton et al., 2017 [42]	USA: New York City (urban)	Income	Public transport	Census tract (2073)
Bereitschaft et al., 2017 [43]	USA: Charlotte, Pittsburgh, Portland (urban)	Income, education, and occupation	Walkability index	Block groups (463)
Bezold et al., 2018 [44]	USA (urban and rural)	Income and education	Greenness index	Individual/household (1,250 m buffer around home) (9385) and census tract
Billaudeau et al., 2011 [45]	France: Paris and surroundings (urban)	Income	Sports facilities	Census blocks (2608)
Botticello et al., 2015 [46]	USA, New Jersey (urban)	Housing	Green, blue, public open spaces and walkability	Community(5 mile/8 km buffer around home)(503)
Brown et al., 2018 [47]	USA: Miami-Dade County (urban)	Income	Greenness index	Census block (36,563)
Buckland et al., 2022 [48]	UK: Birmingham; Belgium: Brussels; Italy: Milan; Czech Republic: Prague; Sweden: Stockholm (urban)	Income	Green, blue, public open spaces	Districts
Buszkiewicz et al., 2022 [49]	USA: Seattle (urban)	Education	Walkability	Individual/household (800 m buffer around home) (819)
Carrier et al., 2016 [50]	Canada: Island of Montreal (urban)	Income	Green, blue, public open spaces	City blocks (10,290)
Carroll et al., 2023 [51]	Australia: Sydney, Melbourne, Brisbane, Adelaide, Perth, Hobart, Darwin, Canberra (urban & rural)	SEP index	Walkability	Individual/household (1,000 m buffer around home) (1675, 1735, 1383, 1067, 1173, 533, 718, 1050)
Casey et al., 2017 [52]	USA: Entire urban USA (urban)	SEP index and housing	Greenness index	Census tract (59,483)
Cereijo et al., 2022 [53]	Spain: Madrid (urban)	SEP index	Sports facilities	Individual/household (1,000 m buffer around home) (186,071, 237,381)
Cereijo et al., 2023 [54]	Spain: Madrid (urban)	SEP index	Sports facilities	Individual/household (1,000 m buffer around home) (1,214,281)
Chandrabose et al., 2022 [55]	Australia: All states (urban & rural)	SEP index	Walkability (components and index), bikeability	Individual/household (1,000 m buffer around home) (3590)
Chaney et al., 2019 [56]	USA: Utah (urban)	Income, education, and housing	Trails	Municipality (33)
Chaparro et al., 2018 [57]	England: Wales, Scotland, London, rest of the regions are not stated (urban & rural)	SEP index	Green, blue, public open spaces	Census area statistic ward (4929)
Cheruvalath et al., 2022 [58]	USA: Milwaukee County, Wisconsin (urban & rural)	SEP index	Green, blue, public open spaces and Greenness index	Census block group (5870)
Choi et al., 2020 [59]	USA: Seattle, Salt Lake City, Fresno: Minneapolos, Kansas City, Grand Rapids, Houston, Raleigh, Brandenton, Boston, Providence, Buffalo (urban)	Income and education	Green, blue, public open spaces	Block groups (14,285)
Choi et al., 2022 [60]	Canada: Toronto (urban)	Income	Green, blue, public open spaces and walkability index	Neighbourhood (140)
Christie et al., 2023 [61]	Canada: 31 cities (urban)	Housing	Walkability index	Dissemination areas (33,026)
Chuang et al., 2023 [62]	New Zealand: Auckland (urban)	Housing	Green, blue, public open spaces	Individual/household (1000) and 300 × 300 m grid centroids (3070)
Clennin et al., 2019 [63]	USA: South Carolina (urban)	SEP index	PA index	Census tract (42)
Cohen et al., 2013 [64]	USA: Philadelphia, Columbus, Chapel Hill/Durham, Albuquerque (urban)	Income	Green, blue, public open spaces	Neighbourhood’s parks (24)
Cohen-Cline et al., 2015 [65]	USA: Washington (urban)	Income and SEP index	Greenness index	Individual/household (1,000 m buffer around home)(4338)
Collyer et al., 2022 [66]	Australia: Perth and Peel metropolitan regions of Western Australia (urban)	SEP index	Green, blue, public open spaces, walkability, bikeability, and public transport	Statistical Area Level 1 (260)
Comer et al., 2008 [67]	USA: Oklahoma City (urban)	Income	Green, blue, public open spaces	Block groups (620)
Conderino et al., 2021 [68]	USA: Midwest, Northeast, South, and West (urban)	Income	Walkability index	City/town (500) and census tract (28,130)
Cote-Lussier et al., 2015 [69]	Canada: Quebec (urban)	Income	Greenness index	Individual/household (500 m buffer around home) (2120)
Cottagiri et al., 2022 [70]	Canada: Victoria, Vancouver, Surrey, Calgary, Winnipeg, Ottawa, Hamilton, Montreal, Sherbrooke, Halifax:, St. John’s (urban)	Income	Greenness index	500 m buffer around postal codes(26,811)
Coutinho et al., 2023 [71]	Norway: Oslo (urban)	SEP index	Green, blue, public open spaces, Sports facilities, public transport	sub-district level defined by administrative boundaries (97)
Cowie et al., 2016 [72]	Australia: Sydney (urban)	SEP index	Walkability index	Census Collection District (5858)
Cradock et al., 2005 [73]	USA: Boston (urban)	Income and education	Playgrounds	Census block groups (591)
Crawford et al., 2008 [74]	Australia: Melbourne (urban)	SEP index	Green, blue, public open spaces, walkability, bikeability, and playgrounds	Public open space locations (1497)
Creatore et al., 2016 [75]	Canada: London, Ottawa, Hamilton, Toronto, and surrounding communities (urban & rural)	Income, education, and occupation	Walkability index	Dissemination areas composed of several adjacent city blocks (8777)
Csomós et al., 2020 [76]	Hungary: Debrecen (urban)	Income and education	Green, blue, public open spaces	Neighbourhood
Csomós et al., 2021 [77]	Hungary: Debrecen, Kecskemet, and Szeged (urban)	Income and education	Greenness index	100 × 100 m grid cells (3093, 2151, and 2540)
Curtis et al., 2024 [78]	USA: Utah (urban)	SEP index	Green, blue, public open spaces and walkability index	Census tract (436)
Cutts et al., 2009 [79]	USA: Phoenix (urban)	Income and education	Green, blue, public open spaces and walkability index	Census block groups (1046)
Dahmann et al., 2010 [80]	USA: Los Angeles (urban & rural)	Income	Recreational courses	Municipality (71)
Darcy et al., 2022 [81]	Australia: Queensland (urban & rural)	SEP index	Walkability, bikeability	Suburb (25)
Davis et al., 2012 [82]	USA: Chicago (urban)	Income	Green, blue, public open spaces	Census tract (822)
DelaBarerra et al., 2019 [83]	Chile: Santiago de Chile (urban)	Income	Greenness index	Neighbourhood (7)
DelaBarrera et al., 2016 [84]	Chile: Santiago de Chile (urban)	Income	Green, blue, public open spaces	Municipality (3)
Dobbs et al., 2023 [85]	Chile: Metropolitan Region of Santiago (urban & rural)	SEP index	Green, blue, public open spaces and green space index	Municipality (52)
Doiron et al., 2020 [86]	Canada: Toronto, Montreal, Vancouver (urban)	SEP index	Greenness index, active transportation index	Postal codes (41,200)
Duncan et al., 2012 [87]	USA: Boston (urban)	Income	Walkability index	Census tract (167)
Edwards et al., 2011 [88]	USA: North Carolina (urban & rural)	Income, education, and SEP index	Recreation index	County (100)
Eime et al., 2017 [89]	Australia: Victoria (urban & rural)	SEP index	Sports facilities	Local government areas (79)
Evans et al., 2012 [90]	Italy: Forlì; Lithuania: Vilnius; Portugal: Ferreira do Alentejo; Germany: Bonn; Switzerland: Geneva; France: Angers; Slovakia: Bratislava; Hungary: Budapest (urban & rural)	Income	Green, blue, public open spaces	Individual/household (1184)
Farkas et al., 2022 [91]	Hungary: Budapest (urban)	Income and education	Greenness index	100 × 100 m Grid cells (23,459)
Fazli et al., 2019 [92]	Canada: Southern Ontario (urban & rural)	Income	Walkability index	Individual/household (1,128,181)
Ferguson et al., 2018 [93]	UK: Bradford (urban)	Income	Green, blue, public open spaces	Lower Layer Super Output Areas (218)
Fernández et al., 2016 [94]	Chile: Santiago de Chile (urban)	SEP index	Greenness index	Census block
Fian et al., 2023 [95]	Austria: Forli, Vilnius, Ferreira do Alentejo, Bonn, Geneva, Angers, Bratislava, Budapest (urban & rural)	Income, education, and occupation	Green, blue, public open spaces and Greenness index	Individual/household (1,000 m buffer around home) (2258)
Field et al., 2004 [96]	New Zealand: Auckland (urban)	SEP index	Green, blue, public open spaces	Statistical Area Level 1 (8770)
Field et al., 2024 [97]	USA: Ohio, New York, Indiana, Illinois, California, Utah, Pennsylvania (urban)	Income	Walkability index	Individual/household (7500 and 9148)
Flacke et al., 2016 [98]	Germany: Dortmund (urban & rural)	Occupation	Green, blue, public open spaces	Neighbourhood (170)
Franzini et al., 2010 [99]	USA: Birmingham (AL): Houston (TX): Los Angeles (CA) (urban)	Income	Walkability, playgrounds	Block-faces (632)
Fraser et al., 2024 [100]	USA: Boston (urban)	Income	Green, blue, public open spaces	Buildings (85,592)
Frey et al., 2017 [101]	USA: Washington D.C. (urban)	Housing	Green, blue, public open spaces	Census tract (175, 46, and 129)
Fuller et al., 2013 [102]	Canada: Montreal (urban)	Income	Bikeability and public transport	Individual/household (6495)
Fuller et al., 2017 [103]	Canada: Calgary, Halifax, Moncton, Montreal, Saskatoon, Toronto, Vancouver, and Victoria (urban)	Income	Bikeability index	Census tract (1282)
Garrison et al., 2019 [104]	USA: New York City (urban)	Income	Green, blue, public open spaces	Census block group (6211)
Giles-Corti et al., 2002 [105]	Australia: Perth (urban)	SEP index	Green, blue, public open spaces and sports facilities	Individual/household (1803)
Gilliland et al., 2006 [106]	Canada: London, Ontario (urban)	SEP index	Recreational opportunities	Dissemination area (22)
Gonzales-Inca et al., 2022 [107]	Finland: Entire urban Finland (urban)	Education, occupation, and SEP index	Green, blue, public open spaces and Greenness index	Individual/household (100 m buffer around home) (14,424, 14,271, 14,375)
Gordon-Larsen et al., 2006 [108]	USA: USA wide (urban & rural)	Education	Sports facilities	Block group (42,857)
Gullón et al., 2017 [109]	Spain: Madrid (urban)	SEP index	Index	Census Sect. (2415)
Gullon et al., 2021 [110]	USA: North Carolina, South Carolina, Georgia, Alabama, Mississippi, Tennessee, Arkansas, and Louisiana (urban & rural)	Income and education	Green, blue, public open spaces, Sports facilities, and walkability	Individual/household (1,000 m buffer around home) (20,808)
Gullón et al., 2023 [111]	Spain: Madrid (urban)	SEP index	Greenness index	Individual/household (200, 300, 500, and 1,000 m buffer around home) (437,513)
Gunn et al., 2022 [112]	Australia: Melbourne (urban)	SEP index	Walkability components and index and public transport	Suburb
Guo et al., 2017 [113]	Hong Kong: Hong Kong (urban)	Income	Green, blue, public open spaces, sports facilities, and public transport	Large street blocks (1629)
Hannon et al., 2006 [114]	USA: Boston (urban)	Income	Sports facilities	Neighbourhood (12)
Harris et al., 2015 [115]	USA: Entire USA (urban & rural)	Income and education	Green, blue, public open spaces	Block groups (216,013)
Hart et al., 2022 [116]	USA: Kentucky (urban)	Income and education	Greenness index	Individual/household (300 m buffer around home) (175)
Heo et al., 2023 [117]	USA: New Haven, Baltimore, Durham (urban)	Income and education	Green, blue, public open spaces and Greenness index	Census block group (2285)
Herrera et al., 2018 [118]	Germany: Munich and Dresden (urban)	Education and occupation	Greenness index	Individual/household (500 m buffer around home) (1632)
Hill et al., 2012 [119]	USA: Danville (Rural)	Income	Green, blue, public open spaces, sports facilities, and walkability index	Block group (39)
Hillsdon et al., 2007 [120]	UK: Entire UK (urban & rural)	SEP index	Sports facilities	Super Output Area (32,482)
Hirsch et al., 2013 [121]	USA: Baltimore, Chicago, Forsyth County, Los Angeles, New York, St. Paul (urban)	Income, education, and occupation	Walkablity index	Individual/household (4552)
Hirsch et al., 2016 [122]	USA: Los Angeles, Chicago, Baltimore, St. Paul, Hinds County, Forsyth County, New York (urban)	Income	Sports facilities	Census tract (8383)
Hirsch et al., 2017 [123]	USA: Birmingham, Chicago, Minneapolis, Oakland (urban)	Income and occupation	Bikeability, public transport, and trails	Neighbourhood (387)
Hobbs et al., 2017 [124]	England: Yorkshire (urban)	SEP index	Green, blue, public open spaces	Individual/household (4723)
Hoekman et al., 2016 [125]	The Netherlands: Entire Netherlands (urban & rural)	SEP index	Sports facilities	Postal code areas
Hoffimann et al., 2017 [126]	Portugal: Porto (urban)	SEP index	Green, blue, public open spaces	Census tract (2064)
Houde et al., 2018 [127]	Canada: Montreal (urban)	Income	Bikeability	Census tract
Hughey et al., 2016 [128]	USA: Southeastern USA (urban)	SEP index	Green, blue, public open spaces	Census block group (255)
Iraegui et al., 2020 [129]	Spain: Barcelona (urban)	Income	Green, blue, public open spaces	Neighbourhood (73)
Iyer et al., 2020 [130]	USA: Pennsylvania (urban & rural)	Income, education, and housing	Greenness index	Individual/household (128,568)
Jamalishahni et al., 2023 [131]	Australia: Brisbane (urban)	SEP index	Green, blue, public open spaces	Individual/household (400, 800, and 1,600 m buffer around home) (3778)
James et al., 2016 [132]	USA: California, Connecticut, Florida, Maryland, Massachusetts, Michigan, New Jersey, New York, Ohio, Pennsylvania, Texas (urban & rural)	Income, education, and housing	Greenness index	Individual/household (250 m buffer around home) (108,603)
Jenerette et al., 2007 [133]	USA: Phoenix (urban)	Income	Greenness index	Census tract (634)
Jepson et al., 2022 [134]	Canada: Montreal (urban)	Income	Green, blue, public open spaces	Dissemination areas and census blocks (6301)
Jimenez et al., 2020 [135]	USA: Massachusetts (urban)	Income and education	Greenness index	Individual/household (90 m buffer around home) (460)
Jimenez et al., 2022 [136]	USA: Whole country (urban & rural)	Income, education, occupation, and housing	Greenness index	Individual/household (270 m buffer around home) (13,594)
Jones et al., 2015 [137]	USA: Los Angeles, Chicago, Baltimore and Baltimore County, St. Paul, Forsyth County, New York City (urban)	Income	Green, blue, public open spaces, sports facilities, parks and recreational facilities	Census tract (7139)
Kamel et al., 2014 [138]	USA: Texas (urban)	Income	Green, blue, public open spaces	Census tract (112)
Kenyon et al., 2019 [139]	Scotland: Glasgow and Edinburgh (urban & rural)	SEP index	Walkability components and index	1000 m network buffer around the output-area centroid(30,066)
Khomenko et al., 2020 [140]	Austria: Vienna (urban)	SEP index	Green, blue, public open spaces	Subdistrict (250)
Ki et al., 2021 [141]	South Korea: Seoul (urban)	Income	Greenness index	Individual/household (500 m buffer around home) (2350)
Ki et al., 2023 [142]	USA: Los Angeles (urban)	Income, education, and vehicle ownership	Walkability index	Block group (2430)
Kiani et al., 2023 [143]	Canada: Montreal (urban)	SEP index	Greenness index	Census tract (689)
Kim et al., 2020 [144]	USA: Austin, Texas (urban)	Income	Green, blue, public open spaces and Greenness index	Census block (457)
Kim et al., 2022 [145]	South Korea: Seoul (urban)	Income	Green, blue, public open spaces	Dong district (424)
Kim et al., 2023a [146]	USA: Birmingham, Chicago, Minneapolis, Oakland (urban)	Income	Green, blue, public open spaces and Greenness index	Individual/household (5,000 m buffer around home) (924)
Kim et al., 2023b [147]	USA: Austin (urban)	Income	Green, blue, public open spaces and Greenness index	Census tract (210)
Kim et al., 2023c [148]	USA: Phoenix (urban)	Income, education, and vehicle ownership	Green, blue, public open spaces	Census block group (2095)
Knight et al., 2018 [149]	USA: Buffalo (urban)	Income and occupation	Walkability index	Census Sect. (284)
Koohsari et al., 2011 [150]	Australia: Melbourne (urban)	SEP index	Green, blue, public open spaces	Census collection district
Koohsari et al., 2020 [151]	Japan: Entire Japan (urban & rural)	SEP index	Walkability index	Municipality (1880)
Koschinsky et al., 2017 [152]	USA: Washington D.C. (urban)	Income	Walkability index	Neighbourhood (115)
Kruize et al., 2007a [153]	The Netherlands: The Rijnmond region (urban)	Income	Green, blue, public open spaces	Individual/household (500 m from home) (445,559)
Kruize et al., 2007b [154]	The Netherlands: Region within a radius of 25 km around Amsterdam (urban)	Income	Green, blue, public open spaces	Individual/household (500 m from home) (1,075,037)
Lafary et al., 2008 [155]	USA: Evansville (urban & rural)	Income and housing	Greenness index	Block groups (159)
Lakes et al., 2014 [156]	Germany: Berlin (urban)	SEP index	Greenness index	Planning units (434)
Lamb et al., 2010 [157]	Scotland: Glasgow, Edinburgh, Dundee, Aberdeen (urban & rural)	Income	Sports facilities	Datazone (6505)
Lane et al., 2022 [158]	USA: Alabama (urban & rural)	Income and education	Physical activity deserts	Census tract (1179)
Lang et al., 2022 [159]	USA: USA wide (urban & rural)	Income, education, and SEP index	Walkability index	Individual/household (12,846)
Langford et al., 2012 [160]	UK: Cardiff (urban)	SEP index	Public transport	Output area (992)
Lee et al., 2007 [161]	Japan: Metropolitan Tokyo and a city from northeastern Japan (urban & rural)	Occupation	Walkability	Individual/household (432)
Lee et al., 2016 [162]	USA: Southeastern Metropolitan Area (urban)	Income, occupation, housing, and SEP index	Walkability index	Individual/household (500)
Lee et al., 2022 [163]	South Korea: Seongman and Daegu (urban)	Income	Green, blue, public open spaces	District (4)
Lee et al., 2023 [164]	Singapore: Singapore (urban)	Education	Greenness index	Individual/household (1,000 m buffer around home) (268)
Leung et al., 2010 [165]	USA: California	SEP index	Walkability	Individual/household (215)
Li et al., 2009a [166]	USA: Portland (urban)	Income and education	Walkability index	Individual/household (1145) and census block groups (120)
Li et al., 2009b [167]	USA: Portland (urban)	Income	Walkability index	Census block groups (120)
Li et al., 2015 [168]	USA: Hartford, Connecticut (urban)	Income, education, and housing	Greenness index	Census block group (87)
Li et al., 2016 [169]	USA: Hartford, Connecticut (urban)	Income, education, and housing	Green, blue, public open spaces	Census block group (90)
Liu et al., 2021 [170]	USA: Chicago: Illinois (urban)	Income	Green, blue, public open spaces	Census tract (801)
Lohmus et al., 2021 [171]	Sweden: Stockholm County (urban & rural)	Income and education	Greenness index	Individual/household (50 m buffer around home) (2060)
Majekodunmi et al., 2020 [172]	Schotland: Glasgow (urban)	SEP index	Green, blue, public open spaces, walkability, bikeability, sports facilities, and playgrounds	Hectare (17,645)
Malecki et al., 2014 [173]	USA: Wisconsin (urban & rural)	Income, education, and SEP index	Green, blue, public open spaces, walkability, bikeability	Individual/household (400 m buffer around home) (890)and census block (939)
Marek et al., 2021 [174]	New Zealand: Whole country (excluding oceanic islands) (urban & rural)	SEP index	Green, blue, public open spaces and sports facilities	Meshblock administrative units (52,923)
Martinuzzi et al., 2018 [175]	USA: San Juan (Puerto Rico) (urban)	Housing and income	Greenness index	Census block groups (789)
Martori et al., 2020 [176]	Spain: Barcelona (urban)	Income	Playgrounds	Census Sect. (1061)
Mavoa et al., 2015 [177]	Australia: Melbourne (urban)	SEP index	Green, blue, public open spaces and sports facilities	Statistical Area level 1 (8910)
McCarthy et al., 2017 [178]	USA: County in southeastern USA (urban & rural)	Income	Playgrounds	Individual/household (0.5 mile/800 m buffer around home) (13,469)
McMorris et al., 2015 [179]	Canada: Entire urban Canada	Income	Greenness index	Individual/household (500 m buffer around home) (69,910)
Mears et al., 2019 [180]	UK: Sheffield (urban)	SEP index	Green, blue, public open spaces	Individual/household (25,264)
Miller et al., 2019 [181]	USA: Tallahassee (urban)	Income	Green, blue, public open spaces	Block group (38)
Moccia et al., 2023 [182]	Italy: Turin (urban & rural)	Income	Walkability, blue spaces, green spaces, public transport	Individual/household (300 m buffer around home)(1989)
Molaodi et al., 2012 [183]	UK: UK wide (urban & rural)	Income	Sports facilities	Lower Super Output Area (32,482)
Moore et al., 2008 [184]	USA: Baltimore city and county, Forsyth County, Manhattan and the Bronx (urban)	Income	Green, blue, public open spaces and sports facilities	Census tract (685)
Mora et al., 2021 [185]	Chile: Santiago de Chile (urban)	SEP index	Bikeability	Household
Motoc et al., 2023 [186]	The Netherlands: Amsterdam, Oss, and Zwolle (urban & rural)	Income, housing, and SEP index	Green, blue, public open spaces	Individual/household (1512)
Mouratidis et al., 2020 [187]	Norway: Oslo (urban)	SEP index	Green, blue, public open spaces and public transport	Neighbourhood (34)
Mustafa et al., 2023 [188]	USA: New York City (urban)	Income	Green, blue, public open spaces	Block group and postal code area
Nayha et al., 2013 [189]	Finland: Oulu, Lapland, and Helsinki metropolitan area (urban & rural)	Education	Walkability	Individual/household (5363)
Neckerman et al., 2009 [190]	USA: New York City (urban)	Income	Bikeability and public transport	Census tract (2172)
Nesbitt et al., 2016 [191]	USA: Portland-Vancouver metro area (urban)	Income and education	Green, blue, public open spaces and Greenness index	Block group
Nesbitt et al., 2019 [192]	USA: Chicago, Houston, Indianapolis, Jacksonville, Los Angeles, New York, Phoenix, Portland, Seattle, and St. Louis (urban)	Income	Green, blue, public open spaces and Greenness index	Block group (5196, 2135, 701, 499, 7574, 12,985, 2012, 830, 1999, 1134, 5195, 2135, 701, 499, 7574, 12,985, 2012, 830, 1999, and 1134) and census tracts (1773, 802, 263, 189, 2680, 2536, 763, 292, 599, 362, 1773, 802, 263, 189, 2680, 2536, 763, 292, 599, and 362)
Nieuwenhuijsen et al., 2018 [193]	Spain: Barcelona (urban)	SEP index	Green, blue, public open spaces and Greenness index	Individual/household (792,649)
Odijk et al., 2023 [194]	The Netherlands: Rotterdam—The Hague metropolitan region (urban)	Income	Bikeability	Postal code
Olsen et al., 2022 [195]	Scotland: Entire Scotland (urban & rural)	Income and SEP index	Green, blue, public open spaces, sports facilities, and public transport	Postal code (146,190)
Olsen et al., 2023 [196]	Scotland: Entire country (urban & rural)	SEP index	Green, blue, public open spaces, walkability, sports facilities, and public transport	Individual/household (800 m buffer around home) (687)
O'Regan et al., 2021 [197]	Ireland: Dublin, Cork, and Galway (urban)	Income, education, and occupation	Greenness index and greenness index	Small Area (3335)
Padilla et al., 2016 [198]	France: Nice metropolitan area (urban & rural)	SEP index	Green, blue, public open spaces	Census block (236)
Panter et al., 2008 [199]	UK: Norwich (urban)	Income and SEP index	Sports facilities	Individual/household (401)
Park et al., 2020 [200]	USA: Metro Columbus and Metro Atlanta (urban)	SEP index	Green, blue, public open spaces, Greenness index, and sports facilities	Block groups (1348 and 2591)
Parker et al., 2021 [201]	USA: Davenport, Bettendorf, Rock Island, and Moline (urban)	Income, education, and occupation	Bikeability	Census block groups (241 and 246)
Pascual et al., 2020 [202]	USA: Rhode Island (urban & rural)	Income	Green, blue, public open spaces	Census tract
Pearce et al., 2010 [203]	UK: Entire UK (urban & rural)	Income	Green, blue, public open spaces	Census Area Statistics Wards (10,654)
Pearsall et al., 2012 [204]	USA: Philadelphia (urban)	Income	Greenness index	Census tract (370)
Pereira et al., 2023 [205]	Portugal: Lisbon Metropolitan Area (urban)	SEP index	Green, blue, public open spaces, walkability, and public transport	Census tract (25,885)
Pham et al., 2011 [206]	Canada: Montreal (urban)	Income	Greenness index	Dissemination Areas (1773)
Pinault et al., 2021 [207]	Canada: Entire Canada (urban)	Income, education, and occupation	Greenness index	Individual/household (500 m buffer around home) (5,306,800)
Plans et al., 2019 [208]	Spain: Madrid (urban)	SEP index	Green, blue, public open spaces	Individual/household (500 m buffer around participant’s census section centroid)(1625)
Pratt et al., 2023 [209]	USA: Franklin county	Income and occupation	Green, blue, public open spaces and sports facilities	Individual/household (0.25 mile/400 m and 0.5 mile/800 m buffer around home)(772)
Pun et al., 2018 [210]	USA: Entire USA (urban & rural)	Income and education	Greenness index	Individual/household (1,000 m buffer around home) (4118)
Quinton et al., 2022 [211]	Canada: Victoria, Burnaby, Coquitlam, Richmond, Surrey, Vancouver, Calgary, Edmonton, Regina, Saskatoon, Winnipeg, Sudbury, Windsor, London, Brampton, Markham, Mississauga, Oakville, Richmond Hill, Toronto, Vaughan, Ottawa, Gatineau, Laval, Longuel, Montreal, Terrebonne, Quebec, Sherbrooke, Halifax, and St. John's (urban)	Income and education	Greenness index	Dissemination Areas
Rachele et al., 2017 [212]	Australia: Brisbane (urban)	SEP index	Walkability, and public transport	Statistical Areas Level 1 (2460)
Ranchod et al., 2014 [213]	USA: Baltimore, Maryland; Chicago, Illinois; Forsyth County, North Carolina; Los Angeles, California; New York, New York; St. Paul, Minnesota (urban & rural)	Income, education, and occupation	Sports facilities	Individual/household (1mle/1,600 m buffer around home)(6168)
Regidor et al., 2008 [214]	Spain: Entire Spain (urban & rural)	Income and education	Sports facilities	Individual/household (17,917)
Richardson et al., 2010 [215]	New Zealand: Whole country (urban)	SEP index	Green, blue, public open spaces	Census area units (1009)
Rigolon et al., 2014 [216]	USA: Denver (Colorado) (urban)	Income	Green, blue, public open spaces	Census blocks (1481)
Rigolon et al., 2017 [217]	USA: Denver (Colorado) (urban)	Income	Green, blue, public open spaces	Census block group (473)
Rigolon et al., 2018 [218]	USA: 99 cities with the largest population in the USA (urban)	Income	Green, blue, public open spaces	City/town (99)
Rivera et al., 2023 [219]	USA: Santa Clara county (California) (urban)	SEP index	Greenness index	Census tract (372)
Robinson et al., 2018 [220]	Norway: Oslo; Lithuania: Kaunas; UK: Bradford; France: Nancy and Poiters; Spain: Gipuzkoa, Sabadell, Valencia, Greece: Heraklion (urban)	Income, education, occupation, and SEP index	Greenness index and green, blue, public open spaces	Individual/household (300 m buffer araound home) (10,559, 3625, 10,008, 669, 574, 594, 575, 695, and 746)
Robinson et al., 2022 [221]	UK: 68 urban centres (urban)	SEP index	Greenness index	Urban centre boundary (68)
Rodgers et al., 2012 [222]	UK: Swansea City (urban)	SEP index	Parks, playgrounds	Individual/household (500 m buffer around home) (103,450)
Rodriguez-Loureiro et al., 2022 [223]	Belgium: Antwerp, Ghent, Brussels, Charleroi, and Liège (urban)	SEP index	Greenness index	Individual/household (500 m buffer around home) (2,309,236)
Rundle et al., 2007 [224]	USA: New York City (urban)	Income	Walkability and public transport	Census tract (1989)
Saelens et al., 2003 [225]	USA: San Diego (California) (urban)	Education	Walkability index	Individual/household (107)
Sallis et al., 2018 [226]	USA: Baltimore (Maryland)-Washington DC, and Seattle-King County (Washington) metropolitan areas (urban)	Income	Walkability index	Census block groups (447)
Saporito et al., 2015 [227]	USA: USA cities with more than 25.000 people, Meadow Woods, and Franklin Town (urban)	Income	Greenness index	Census block group
Schaeffer et al., 2019 [228]	France: Grenoble-Alpes Metropole (urban)	Income	Greenness index	Individual/household (184,485)
Scheurer et al., 2017 [229]	Australia: Melbourne, Sydney, Adelaide, and Brisbane (urban)	SEP index	Public transport	Statistical Area 1
Schinasi et al., 2023 [230]	USA: Philadephia (urban)	Income	Greenness index	Census tract (376)
Schneider et al., 2015 [231]	Germany: Cologne (urban)	Income and occupation	Sports facilities	Social areas (18)
Schneider et al., 2019 [232]	Germany: Mannheim (urban)	Income and occupation	Playgrounds	Social areas (44)
Schüle et al., 2017 [233]	Germany: Munich (urban)	SEP index	Green, blue, public open spaces	Sub districts (108)
Sharifi et al., 2021 [234]	Australia: Melbourne (urban)	Income	Greenness index	Statistical Areas Level 2 (306)
Shih et al., 2022 [235]	Taiwan: Taipei city (urban)	Income and education	Green, blue, public open spaces and Greenness index	Neighbourhood (991)
Shin et al., 2023 [236]	South Korea: Seoul (urban)	SEP index	Walkability, bikeability	Haengjeongdon (smallest administrative unit within Seoul) (422)
Slater et al., 2022 [237]	USA: Alabama, Florida, Georgia, Kentucky, Louisiana, Maryland, Missouri, North Carolina, South Carolina, Tennessee, Texas (urban & rural)	Income	Walkability, bikeability	Street segment (4363)
Spotswood et al., 2021 [238]	USA: 17 states (urban)	Income	Green, blue, public open spaces and Greenness index	Census block group (142,325)
Stucki et al., 2023 [239]	Sweden: Uppsala and Västmanland (urban)	Income	Greenness index	Individual/household (500 m buffer around home) (20,244)
Suárez et al., 2020 [240]	Norway: Oslo Metropolitan Area (urban)	Income	Green, blue, public open spaces	Census tract
Subiza-Perez et al., 2023 [241]	Spain: Asturias, Gipuzkoa, Sabadell, and Valencia (urban & rural)	SEP index and education	Green, blue, public open spaces and Greenness index	Individual/household (100, 300, and 500 m buffer around home) (1738)
Sugiyama et al., 2015 [242]	Australia: Metropolitan Adelaide, South Australia (urban)	SEP index	Walkability	Individual/household (1,000 m buffer around home) (1500)
Suminski et al., 2011 [243]	USA: Large Midwestern metropolitan area (urban)	Income	Green, blue, public open spaces	Neighbourhood (16)
Sun et al., 2021 [244]	USA: Los Angeles County (urban & rural)	Income, education, housing, and SEP index	Green, blue, public open spaces and Greenness index	Census tract (2343)
Svastisalee et al., 2012 [245]	Denmark: Copenhagen (urban)	Income, education, and SEP index	Green, blue, public open spaces, walkability, bikeability, and sports facilities	Rodes (389)
Tan et al., 2017 [246]	Singapore: Main island of Singapore (urban)	Income	Green, blue, public open spaces	Region (323), planning area (55), and subzone (5)
Tayyebi et al., 2016 [247]	USA: Metropolitan Los Angeles (South California) (urban)	Income	Greenness index	Census block
Thornton et al., 2016 [248]	USA: San Diego (urban)	Income	Walkability	Block group (111)
Timperio et al., 2007 [249]	Australia: Melbourne (urban)	SEP index	Green, blue, public open spaces and sports facilities	Postal code (177)
Tiznado-Aitken et al., 2022 [250]	Chile: Santiago (urban)	SEP index	Bikeability	Individual/household (6,075,760)
Vallee et al., 2020 [251]	Canada: Montreal Metropolitan Area (urban)	Education	Green, blue, public open spaces, bikeability, and sports facilities	Individual/household (0.5 mile/800 m buffer around home) (1101)
van Diepen et al., 2023 [252]	The Netherlands: Northern Netherlands (i.e.: Groningen, Friesland, Drenthe) (urban & rural)	SEP index	Sports facilities	Individual/household (1,000 m buffer around home) (146,629)
Van Velzen et al., 2023 [253]	The Netherlands: Entire Netherlands (urban & rural)	Income, education, and occupation	Green, blue, public open spaces	School (5773)
Vaughan et al., 2013 [254]	USA: Kansas City (urban & rural)	Income	Green, blue, public open spaces	Census tract (170)
Venter et al., 2023 [255]	Norway: Oslo (urban)	Income	Green, blue, public open spaces and Greenness index	Sub-district (delbydel) (99)
Villanueva et al., 2016 [256]	Spain: Madrid (urban)	Occupation and housing	Sports facilities	Individual/household (727)
Wang et al., 2019 [257]	USA: Miami-Dade county (urban)	Income	Greenness index	Postal code (249,405)
Wang et al., 2022 [258]	Taiwan: Taichung City (urban)	Income and education	Green, blue, public open spaces	Census block group (108)
Wen et al., 2013 [259]	USA: Entire USA (urban & rural)	Income	Green, blue, public open spaces	Census tract (11,079, 17,067, 12,654, 15,857, 3648, and 11,458)
Wende et al., 2022 [260]	USA: Northeast, Midwest, South, and West USA (urban & rural)	Income	Physical Activity Environment index	County (217, 1055 1422, 448, 1166, 1335, and 641)
Willis et al., 2023 [261]	USA and Canada: Entire USA and Canada (urban & rural)	Income and education	Greenness index	Individual/household (50 m buffer around home) (8563)
Witten et al., 2011 [262]	New Zealand: North Shore, Waitakere, Wellington, and Christchurch (urban)	SEP index	Neighbourhood destination index	Meshblock (1577, 1338, 1807, 2880)
Wolch et al., 2005 [263]	USA: Los Angeles (urban)	Income	Green, blue, public open spaces	Census tract (720)
Wüstemann et al., 2017 [264]	Germany: 53 major cities (urban)	Income and education	Green, blue, public open spaces	Individual/household (500 m buffer around home) (4404)
Yang et al., 2019 [265]	Canada: Edmonton (urban)	Income, education, and housing	Sports facilities	School (281)
Yasumoto et al., 2021 [266]	Japan: Osaka (urban)	Occupation	Green, blue, public open spaces	Small census areas 'Chocho-aza'
Yeager et al., 2018 [267]	USA: Louisville (urban)	Income and SEP index	Greenness index	Individual/household (250 m buffer around home) (408)
Yeager et al., 2023 [268]	USA: Louisville, Kentucky (urban)	Income, education, and occupation	Greenness index	Individual/household (100 m buffer around home) (636)
Younan et al., 2016 [269]	USA: Los Angeles county and surrounding areas (urban)	SEP index	Greenness index	Individual/household (1,000 m buffer around home) (1287)
Yu et al., 2014 [270]	USA: Austin (urban)	Income	Walkability, bikeability	Census tract (162)
Zandieh et al., 2017 [271]	UK: Birmingham (urban)	SEP index	Green, blue, public open spaces, walkability	Neighbourhood (173)
Zandieh et al., 2019 [272]	UK: Birmingham (urban)	SEP index	Green, blue, public open spaces	Individual/household (173)
Zhang et al., 2021 [273]	Hong Kong (urban)	SEP index	Green, blue, public open spaces	Tertiary planning units (209)
Zhang et al., 2022 [274]	USA: Fayetteville, Winston-Salem, Charlotte, Raleigh (urban)	SEP index and income	Green, blue, public open spaces	Census tract and school (42, 36, 91, and 97)
Zhang et al., 2023a [273]	USA: Hartford (urban)	Income, education, and housing	Green, blue, public open spaces	Census block group
Zhang et al., 2023b [275]	Hong Kong (urban)	Income, education, and occupation	Green, blue, public open spaces and Greenness index	Individual/household (1977)
Zhang et al., 2024 [276]	New Zealand: Auckland (urban)	SEP index	Green, blue, public open spaces	Individual/household (3813)
Zhou et al., 2023 [277]	Ireland: Belfast (urban)	Income, education, and occupation	Green, blue, public open spaces	Community
Zhu et al., 2008 [278]	USA: Austin (urban)	Income	Walkability	School's attendance area (73)

For some studies, the number of units analysed was unclear. Therefore, not for all units an n is reported

*SEP* Socioeconomic position

### Quality assessment

More than half of the studies (57.6%) were of fair quality. Over a third (36%) were of good quality, and smallest number of studies (6.4%) were of poor quality. The quality assessments can be found in Supplementary File 7.

### Analysis of socioeconomic inequalities in exposure to neighbourhood physical activity environments

**Fig. 2:**
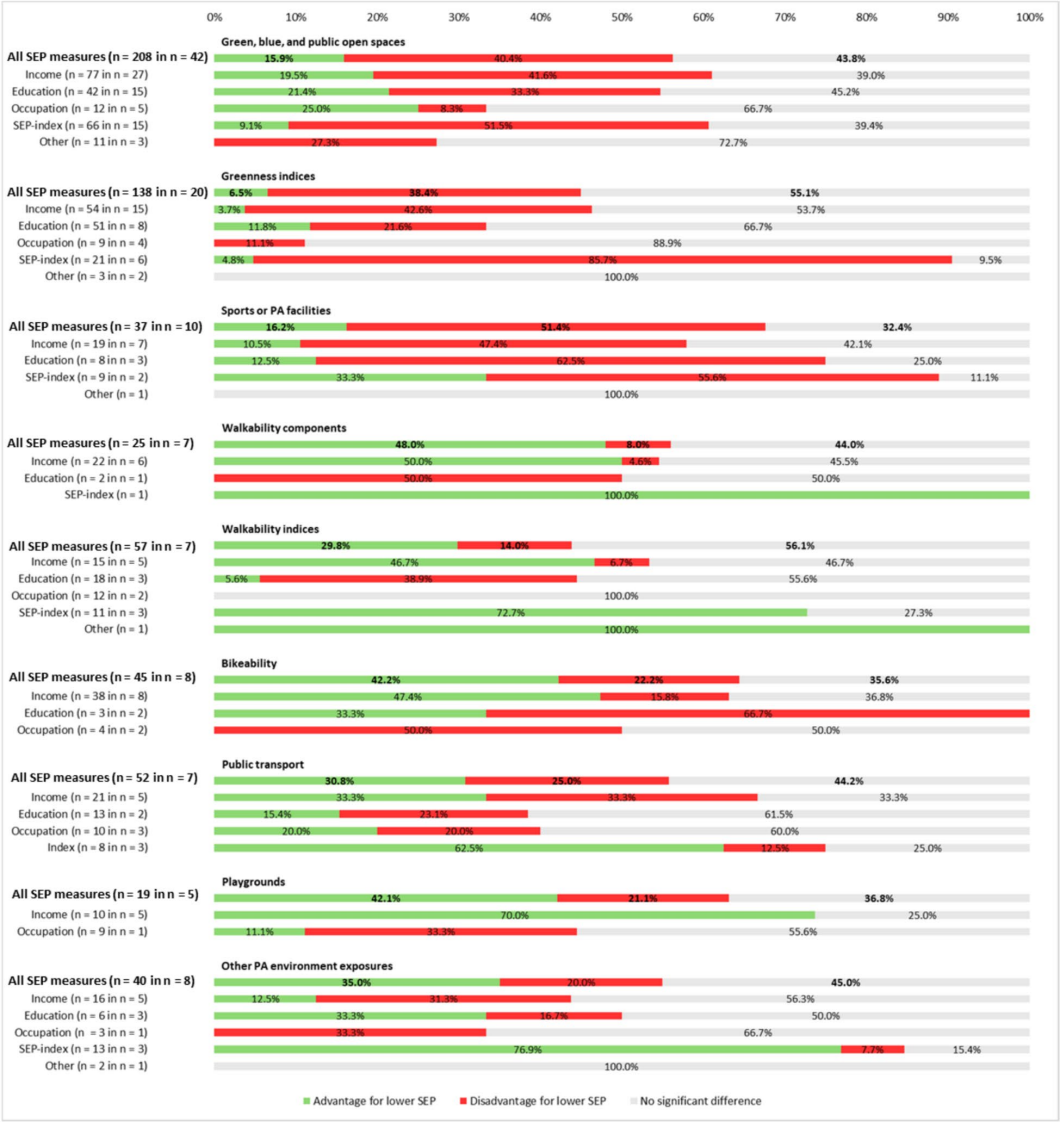
Regression results for SEP and PA environment exposure, stratified by PA resource and SEP proxy. SEP: Socioeconomic position; ‘notation ‘n = … in n = …’ indicates the number of associations (first n) and the number of studies from which these associations were derived (second n)

**Fig. 3:**
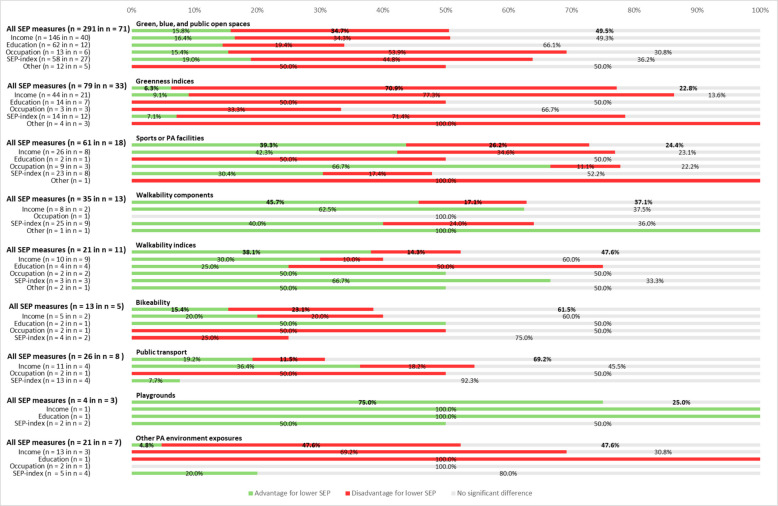
Non-regression statistical results for SEP and PA environment exposure, by PA resource and SEP proxy. SEP: Socioeconomic position; notation ‘n = … in n = …’ indicates the number of associations (first n) and the number of studies from which these associations were derived (second n)

Figures 2 and 3 present the proportions of associations indicating either an advantage, a disadvantage, or no statistically significant difference in exposure to neighbourhood PA resources. Throughout the Results, ‘advantage’ refers to advantageous (i.e., higher) exposure among populations with lower SEP, and ‘disadvantage’ refers to disadvantageous (i.e., lower) exposure among groups with lower SEP. The findings are shown separately for regression-based analyses and other statistical methods. The results are structured by type of PA resource and by how SEP was operationalised. Results on descriptive statistics are not included in the main review but are available in Supplementary File 8. Additional analyses that compare studies conducted in the USA versus other countries, adults/older adults versus children, and individual-level versus ecological studies can also be found in Supplementary File 8.

### Green, blue, and public open spaces

Exposure to green, blue, and public open spaces was measured in 117 studies, and operationalised in diverse ways. Common measures included distance to the nearest park or green/blue area (e.g., [255, 264]), vegetation cover within defined buffers (e.g., [50, 104]) and indicators of green space availability such as the proportion of land designated as green space (e.g., [119]) or green space per inhabitant (e.g., [31]). A full overview of all operationalisations is provided in the extended descriptive table (Supplementary File 6).

Regression analyses using income or a SEP index generally indicated disadvantage for individuals or areas with lower SEP. When SEP was measured by education, occupation, or housing, most associations were not statistically significant. In non-regression analyses, non-statistically significant findings also predominated overall. Stratifying the non-regression analyses by SEP indicator revealed some variation: results on income and education were mostly non-significant; occupation and SEP indices more often indicated a disadvantage; and housing-related results were evenly split between disadvantage and non-significance. Across stratifications, most associations (63.7%) came from non-USA studies, the majority were conducted at the ecological level (85.3%), and over half (56.3%) focused on adults or older adults. Patterns were consistent across subgroups: the largest share of associations was non-statistically significant, around one-third indicated disadvantage for groups with lower SEP, and the smallest proportion showed an advantage.

### Greenness indices

There were 69 studies that assessed greenness exposure measured with indices. Most studies in this category used NDVI to quantify greenness exposure. NDVI was typically operationalised as a continuous measure within buffers of varying sizes around participants’ homes or postal codes (e.g., [70, 95]), categorised into tertiles or other quantiles (e.g,. [267]), or summarised as the mean NDVI value for a given area (e.g., [238]). Regression analyses using greenness indices (e.g., Normalised Difference Vegetation Index (NDVI)) were mostly non-significant, except when SEP was measured by an index, where disadvantage for lower SEP groups was more common. Non-regression analyses showed a disadvantageous pattern across all SEP indicators, except for occupation. Most associations (61.8%) came from USA-based studies. In non-USA studies, over half (56.7%) were non-significant, whereas in USA studies, 75.9% indicated disadvantage. Advantage for lower SEP groups was least common in both contexts (2.4% USA, 9.0% non-USA). Regarding level of analysis, two-thirds (66.4%) of associations were ecological; in both ecological and individual-level studies, results were split roughly evenly between disadvantage and non-significance, with advantage occurring the least common (3.4% ecological, 12.9% individual). Stratified by age, most associations (81.1%) focused on adults or older adults, with 51.7% non-significant and one-third disadvantageous. For children, 71.4% indicated disadvantage for communities with lower SEP.

### Sports facilities

Exposure to sports facilities was examined in 38 studies. The types of facilities assessed varied across studies and included, for example, gyms [105, 108, 199], swimming pools [105, 108, 120], golf courses [105, 108, 200], and tennis clubs [105]. Some studies assessed the total number of sports facilities or the number of facilities per population unit within the study area (e.g., [45, 54, 71, 120, 174, 214, 256]). A complete overview of all sports facility types is provided in the extended descriptive table (Supplementary Table 6). Regression analyses generally showed lower exposure to sports facilities among lower SEP populations. Non-regression analyses showed more mixed patterns: income- and occupation-based analyses more often indicated advantage, education-based analyses were evenly split between disadvantage and non-significance, and most SEP index-based results were non-significant. Most associations (92.4%) came from non-USA studies, where results were evenly distributed between disadvantage, advantage, and non-significance. In USA studies, 68.2% indicated disadvantage and 31.8% were non-significant. Most associations (83.7%) were ecological, showing patterns similar to individual-level analyses, with an even distribution across outcome directions. Stratified by age, 60% of associations with a defined age group focused on children. Here, half were non-significant, 29.2% indicated disadvantage, and the rest advantage. Among adults and older adults, half showed advantage, 37.5% were non-significant, and the remainder disadvantage.

### Walkability (individual components)

Walkability components were measured in 27 studies. Commonly assessed were land use mix (e.g., [46, 242, 278]), intersection density (e.g., [55, 66, 245, 278]), destination or amenity availability (e.g., [51, 110]), and sidewalk characteristics such as presence (e.g., [237, 248]) completeness (e.g., [270]), and density (e.g., [173, 236]).

Regression analyses using income generally indicated advantage for lower SEP groups; the single SEP index-based association also showed advantage. Of two education-based associations, one was disadvantageous and one non-significant. Non-regression analyses using income, SEP indices, or housing most often showed an advantage, whereas occupation-based results were generally non-significant. Associations were split about evenly between USA and non-USA studies. In the USA, advantage was slightly more common (50%), followed by non-significance (43.8%). In non-USA studies, 40% were non-significant and 36.7% indicated advantage. Most associations (59.7%) were ecological. Both for ecological- and individual-level studies, advantageous results were most common. By age group, most associations (64%) focused on children; in both age groups, non-significance predominated, followed by advantage in over one-third of associations.

### Walkability indices

A total of 30 studies explored neighbourhood walkability using walkability indices. Walk Score was the index most frequently used to quantify walkability. It was commonly operationalised as a continuous score within varying buffer sizes around people’s homes or neighbourhoods (e.g., [55, 75, 149]), dichotomisation of lower versus higher walkability (e.g., [43, 79]), as a neighbourhood level mean score (e.g., [68]), or categorised into quantiles such as quintiles of the continuous score (e.g., [159]). As Walk Score was originally developed for the USA and is most widely applied in this region, studies conducted in other regions often used alternative walkability indices, typically combining components such as land-use mix, intersection density, and destination availability (e.g., [75, 79, 151]).

Regression analyses using income were mostly split between advantage and non-significant (both 46.7%). For education- and occupation-based studies, results were mostly non-significant. Analyses using SEP indices or vehicle ownership more often indicated advantage for lower SEP groups. In non-regression analyses, income-based results were mainly non-significant, while education-based results more often indicated disadvantage, and SEP index-based results indicated advantage. Occupation- and housing-based results were evenly split between advantage and non-significance. Most associations (60%) came from USA-based studies; in both USA and non-USA contexts, over half were non-significant, with just over 30% indicating advantage. This same pattern emerged across levels of analysis, with slightly more than half (52.6%) of associations on the individual level. The same pattern also held for adults and older adults. No associations specifically on children were reported.

### Bikeability

Bikeability of neighbourhoods was assessed in 14 studies, using a wide range of operationalisations. Measures included distance to nearest cycling network (e.g., [127]), dichotomous indicators of having access to the cycling network within a defined buffer (e.g., [127, 201, 237]) and bike-lane completeness (e.g., [270]). A full overview of all bikeability operationalisations is provided in the extended descriptive table (Supplementary File 6). Regression analyses using income mainly indicated advantage for lower SEP groups. For education, two-thirds of associations were disadvantageous, while occupation-based results were evenly split between disadvantage and non-significance. Non-regression analyses using income or SEP indices were mostly non-significant, while education-based results were split between advantage and non-significance. For the two non-regression associations using occupation to indicate SEP, one was disadvantageous and one was non-significant. Most associations (63.8%) came from non-USA studies, where non-significance (42.9%) was slightly more common than disadvantage (38.1%). In USA studies, advantage was most common (46%), followed by non-significance (40.5%). The majority of associations (91.4%) were at the ecological level. Here, a slight majority (41.5%) was non-significant, and 35.9% showed an advantage. Among individual-level associations, both advantageous and non-significant findings comprised 40% of the results. For adults and older adults, half of associations showed advantage and half non-significance. The single association on children indicated disadvantage.

### Public transport

Public transport exposure was measured in 18 studies, and was operationalised in various ways. Measures used were for example number of transport stops (e.g., [71, 195]), distance to public transport (e.g., [102, 113]), and proportion of the population with access to public transport within a defined buffer (e.g., [190, 195]). Regression analyses showed mixed results: for income, associations were evenly split between disadvantage, advantage, and non-significance; for education and occupation, most were non-significant; SEP index-based analyses generally indicated advantage. Non-regression analyses predominantly showed non-significant differences. Most associations came from non-USA studies (66.7%), were ecological (59%), and focused on adults or older adults (91.2%). In nearly all subgroups, around half of associations were non-significant, about 30% indicated advantage, and roughly 20% disadvantage. For children, two-thirds of associations were non-significant and one-third indicated advantage.

### Playgrounds

Nine studies examined playground exposure, mostly operationalised as playground availability in a certain buffer area [41, 74, 172, 232] or distance to nearest playground [73, 176, 178, 232]. Regression analyses using income mostly indicated advantage for lower SEP groups, while occupation-based results were generally non-significant. Among the four non-regression analyses, most indicated advantage. Most associations (82.6%) came from non-USA studies, where non-significance (42.1%) was slightly more common than advantage (36.8%). All four USA-based associations indicated advantage. Most associations (91.3%) were ecological; here, advantage (45.5%) was slightly more common than non-significance (36.4%). Both individual-level associations indicated advantage. All nine age-specific associations focused on children, showing an even split between advantage (44.4%) and disadvantage (44.4%).

### Other environmental PA resources

This category included 15 studies that used exposures not fitting into the above mentioned PA resource types. Examples are combined walking/cycling paths [173, 245], combined measures of parks/playgrounds [222], or general PA environment indices (e.g., [260]). Regression analyses were mostly non-statistically significant, except SEP index-based results, which predominantly indicated advantage. Non-regression analyses showed disadvantage for lower SEP groups when SEP was measured by income or education, but non-significance when using occupation or SEP indices. Most associations (68.9%) came from USA studies; of these, over half (54.8%) were non-significant and 38.1% indicated disadvantage. In non-USA studies, advantage was most common (63.2%), followed by non-significance (26.3%). Most associations (78.7%) were ecological, with non-significance most common (54.2%), followed by disadvantage (35.4%). At the individual level, advantage predominated (76.9%). For children, 78.6% of associations indicated advantage; no associations were specific to adults or older adults.

## Discussion

This systematic review summarised 1,174 statistical associations and 254 summary statistics within 250 studies, to assess socioeconomic inequalities in exposure to neighbourhood PA environments in high-income countries. In general, regression-based results indicated that populations with relatively lower SEP were more exposed to walkable neighbourhood features, bikeable infrastructure, and playgrounds in and around their neighbourhood, and less exposed to sports facilities. Advantages and disadvantages for populations with lower SEP vary by PA resource and SEP indicator, and also by country, age group, and unit of analysis. Throughout both the main analyses and stratified analyses, null results remain common. Overall, these findings imply that socioeconomic inequalities in exposure to PA environments are nuanced rather than uniform.

Given the inconsistent exposure inequalities observed, our findings indicate that differences in exposure to neighbourhood PA environments are unlikely to fully account for the well-documented socioeconomic inequalities in PA behaviours and obesity outcomes. Regarding the impact of the built environment on health inequalities, it should be acknowledged that people are typically exposed to a combination of neighbourhood characteristics rather than to a single environmental feature. Besides the neighbourhood PA environment, food environments may also be associated with patterns of health inequalities. For instance, access to green space and active transport infrastructure may encourage PA, but their potential benefits could be offset in areas where people have limited access to affordable healthy foods (e.g., fruit- and vegetable stores) or are heavily exposed to unhealthy foods (e.g., fast-food outlets). Considering combined environmental exposures could help to better understand the broader environmental contexts in which socioeconomic health inequalities arise.

Our findings partly align with those of Jacobs et al. [23], who also found that walkability tended to (marginally) favour populations with lower SEP. In contrast, whereas Jacobs et al. and Høyer-Kruse et al. [22] found a slight disadvantage for lower SEP regarding bikeability, we observed an advantage. This difference may be explained by our inclusion of 31 associations published after Jacobs et al.’s 2018 search, more than half of which indicated an advantage. Although the review by Høyer-Kruse et al. is more recent, its scoping-review design and broader inclusion criteria (e.g., including reviews and citizen-science studies) yielded fewer and partly different bikeability results. Furthermore, while Jacobs et al. identified a slight advantage for people with lower SEP in exposure to recreational facilities (categorised as ‘sports facilities’ in our review), our review includes 23 post-2018 associations, which showed predominantly disadvantageous results for lower SEP. Both Jacobs et al. and our review, however, consistently highlight the prevalence of mixed and null results across most PA resource types.

The overall regression-based results for walkability components and walkability indices did not fully align. This may be attributable to the unequal weighing of income as a SEP indicator on the results: With regard to walkability components, 22 of the 25 associations (88%) used income as the SEP indicator, compared with 15 of the 57 associations (26%) for walkability indices. In both cases, income was predominantly associated with advantageous walkability for people with lower SEP. However, as income represented a larger proportion of the walkability component results, the overall proportion of advantageous findings was considerably higher for components than for indices.

A different type of discrepancy was observed in the results for green space exposure. While both categories of green space exposure (green, blue, and public open spaces and greenness indices) yielded predominantly null results, the proportion of advantageous associations for lower SEP was higher for green, blue, and public open spaces (15.9%) than for greenness indices (6.5%). This may reflect differences in measurement: most measures in the first category captured exposure to parks (e.g. mean park area per city block or number of parks per census tract), whereas greenness indices primarily used Normalised Difference Vegetation Index (NDVI). NDVI is a satellite-based measure of overall greenness that does not distinguish between public and private vegetation [279]. It may therefore also capture vegetation that is not accessible for PA. Measures reflecting actual access to usable green spaces may better capture socioeconomic disparities relevant to PA.

This review has several strengths. Firstly, the review had a broad scope, while we also aimed to capture nuances. We searched four major databases and included studies from all high-income countries over a 25-year period. Secondly, the analysis was detailed and inclusive, incorporating all statistical outcomes and a wide range of SEP indicators and environmental PA resources. Results were stratified by analytical approach, SEP indicator, geographical context and level of analysis. These features could make this review a valuable resource for researchers, urban planners, and policymakers working in diverse settings.

Some limitations should be acknowledged. A methodological limitation of this review is that the proportions of (dis)advantage/null results were based on the number of reported associations per PA resource. This approach was chosen to ensure a comprehensive and consistent synthesis across a heterogeneous body of literature, but it may have led to studies reporting multiple associations per PA resource having a disproportionate influence on the aggregated findings. To assess the extent of this influence, we conducted robustness checks in which, for each PA-resource category, we excluded the study that contributed the largest number of associations. Full results of these robustness checks are provided in Supplementary File 9. Several methodological limitations were evident across the studies included in this review. Neighbourhoods were frequently defined using administrative boundaries that may not correspond to individuals’ lived environments. Additionally, inconsistency in findings across studies likely reflects methodological differences, including variations in how SEP is defined, how exposure to PA environments is measured, the geographical contexts examined, and the analytical approaches employed. This heterogeneity made it difficult to draw generalisable conclusions or to conduct a meta-analysis. Greater harmonisation in how exposure to PA resources and SEP are defined and measured would facilitate comparisons across studies and strengthen future evidence syntheses.

The results of this review should be interpreted with caution, as they reflect relative inequalities. For example, while people with lower SEP may appear to have greater exposure to walkable infrastructure, this does not reveal whether overall exposure is adequate or insufficient across all groups. Such relative comparisons can obscure absolute exposure levels, making it harder to identify which PA resources require policy attention. Additionally, our synthesis was based on reported statistical significance, with all non-significant findings classified as ‘null’. From an equity perspective, however, the absence of a statistically significant difference does not guarantee fairness. Populations with a lower SEP more often suffer from overweight or obesity and other health risks, and have a shorter (healthy) life expectancy [280–284]. Thus, even when exposure to PA-promoting environments appears equal, this may not necessarily translate into equal health outcomes.

Importantly, advantageous exposure to PA-promoting environments does not automatically translate into higher levels of PA. As Jacobs et al. [23] also noted, individual and social factors play a critical role in shaping PA behaviours. A systematic review of qualitative studies on PA determinants found that, beyond perceptions of the urban environment, factors such as financial constraints, work-life balance, community engagement, social support, and psychosocial stressors significantly influence PA participation among populations with lower SEP [285]. Moreover, previous research has shown that limited availability of PA resources was more strongly associated with obesity and type 2 diabetes among populations living in areas with lower SEP [53, 54], suggesting that susceptibility to PA environments may also follow a social gradient. Socioeconomic inequalities in PA behaviours additionally differ by PA domain: people with a lower SEP engage more often in occupational PA, whereas leisure-time PA is more prevalent in people with a higher SEP [7]. This latter domain has been more consistently associated with health benefits [286, 287]. Together, these findings highlight that inequalities in health behaviours and outcomes likely arise from a complex interaction of structural, environmental, social, material, and biological factors [288]. Equity-focused policy responses are needed to ensure that all population groups not only have access to, but are also able to make use of, health-promoting PA environments.

Building on the findings of this review, future research should aim to further improve our understanding of socioeconomic inequalities in exposure to environments that promote PA. Because this review focused specifically on quantitative exposure measures (e.g., density, distance, availability), it did not capture qualitative aspects of neighbourhood PA environments. Yet, the quality and safety of neighbourhood PA environments, such as the variety of playground features, park quality, and perceived fear of crime, have been linked to PA behaviour and health outcomes [279, 289–292]. Combining evidence on SEP inequalities in these neighbourhood characteristics with our results of quantitative, objective measures of exposure could provide a more comprehensive understanding of SEP differences in exposure to obesogenic environments and help identify where interventions are most needed.

Given the inconsistent evidence in our review, policymakers should be cautious about assuming uniform SEP inequalities in PA environments across settings, and should instead focus on the local context. Rather than assuming uniform (dis)advantages for people with a lower or higher SEP, decisions on investing in parks, playgrounds, and active transport infrastructure that promote equitable exposure to the PA environment should be made in cooperation with local communities. Furthermore, it is essential to address the other barriers (e.g. financial constraints and work-life balance) that hinder groups with a lower SEP from achieving sufficient PA. Communities should be actively involved in formulating concrete solutions that reflect their actual needs and priorities.

## Conclusions

Individuals and neighbourhoods with a lower SEP were more exposed to certain PA environments (walkability features, bikeability, and playgrounds), but less exposed to sports facilities. Across most PA resources, the majority of results indicated no statistically significant SEP differences in exposure. To assess and promote equity in PA environments, attention must be paid at a local level, and community perspectives must be incorporated to ensure that interventions address local needs.

## Supplementary Information

Supplementary Material 1.

Supplementary Material 2.

Supplementary Material 3.

Supplementary Material 4.

Supplementary Material 5.

Supplementary Material 6.

Supplementary Material 7.

Supplementary Material 8.

Supplementary Material 9.

## Data Availability

The datasets used and analysed during the current study are available from the corresponding author on reasonable request.

## Abbreviations

SEP: Socioeconomic position
PA: Physical activity
USA: United States of America
UK: United Kingdom
