# Supplementary material for: Socioeconomic inequalities in exposure to neighbourhood environments for physical activity: a systematic review
Source: Int J Behav Nutr Phys Act. 2026 Apr 9;23:58. doi: 10.1186/s12966-026-01912-1 (PMC13231669; doi:10.1186/s12966-026-01912-1)
Supplement: Supplementary file 2 — Supplementary Material 2. [file 12966_2026_1912_MOESM2_ESM.pdf]

Search histories and results February 26, 2024

Search History Medline (Ovid) February 26, 2024

Set	Medline (Ovid) Query	Results
#1	Exp Social Class/ OR exp Socioeconomic Factors/ OR exp Poverty/ OR exp Poverty Areas/ OR Income/ OR Educational Status/ OR Residence Characteristics/ OR exp Social Determinants of Health/ OR (socioeconomic* OR socio-economic* OR "social class*" OR "social status" OR "social determinants of health" OR poverty OR deprivation OR deprived OR income* OR education* OR occupation* OR "residence characteristic*" OR SEP OR SES OR wealth*).ti,ab,kf	1,667,299
#2	Exp Health Inequities/ OR (inequalit* OR equalit* OR difference* OR inequit* OR equit* OR disparit* OR discrepanc* OR contrast* OR dissimilarit* OR disadvant* OR advant* OR injustic* OR justic*).ti,ab,kf	4,906,227
#3	Exp Built Environment/ OR exp Exposome/ OR exp Access to Healthy Foods/ OR exp Food Deserts/ OR exp Environment Design/ OR exp Parks, Recreational/ OR Food Services/ OR Food Supply/ OR exp Restaurants/ OR exp Sports and Recreational Facilities/ OR Diet/ec OR exp City Planning/ OR (obesogenic* OR "city planning" OR "urban planning" OR "environmental planning" OR (neigh* ADJ6 planning) OR ((urban OR neigh* OR environment*) ADJ6 design*) OR "neighbourhood characteristic*" OR "neighborhood characteristic*" OR "urban form" OR sprawl OR zoning OR "residential location*" OR (environment* ADJ6 ("built" OR food* OR "physical activit*" OR "physical inactiv*" OR "PA" OR walking OR cycling OR "green" OR "workplace" OR "natural" OR "injustice" OR "design*")) OR "objective environment*" OR "measured environment*" OR "exposome" OR "parks" OR "green space*" OR "green infrastructure*" OR "natural space*" OR "natural infrastructure*" OR "food desert*" OR "food services" OR restaurant* OR supermarket* OR (facilit* ADJ6 (sport* OR "physical activity" OR "PA" OR "recreational" OR exercise)) OR ((outlet* OR supply) ADJ6 (food OR fastfood OR takeaway* OR "take-away*")) OR "food deliver*" OR "convenience store*" OR "takeaway food" OR "take-away food" OR "food store*" OR "food desert*" OR "nutrient densit*" OR "walkability" OR "walkable" OR "driveability" OR "driveable" OR "job densit*" OR "population densit*" OR "intersection" OR "land use mix" OR "land-use mix" OR "public transport*" OR sidewalk* OR "green space*" OR playground* OR play-ground* OR bikeab* OR cycleab* OR pedestrian OR "cycle path*" OR cyclepath*).ti,ab,kf	183,044
4	Exp Spatial Analysis/ OR exp Access to healthy foods/ OR exp Geographic Information Systems/ OR (exposure* OR proximit* OR densit* OR access* OR distance* OR availabilit* OR geospatial OR spatial OR "geographic* information system*" OR "GIS").ti,ab,kf	3,524,442
5	1 AND 2 AND 3 AND 4	5716

Search History Embase.com February 26, 2024

Set	Embase.com Query	Results
#1	'social status'/exp OR 'socioeconomics'/exp OR 'residence characteristics'/exp OR 'social determinants of health'/exp OR (socioeconomic* OR socio-economic* OR 'social class*' OR 'social status' OR 'social determinants of health' OR poverty OR deprivation OR deprived OR income* OR education* OR occupation* OR 'residence characteristic*' OR SEP OR SES OR wealth*):ti,ab,kw	2,985,412
#2	'health disparity'/exp OR (inequalit* OR equalit* OR difference* OR inequit* OR equit* OR disparit* OR discrepanc* OR contrast* OR dissimilarit* OR disadvant* OR advant* OR injustic* OR justic*):ti,ab,kw	6,454,436

#3	'built environment'/exp OR 'environmental planning'/exp OR 'exposome'/exp OR 'healthy food access'/exp OR 'food desert'/exp OR 'environmental planning'/exp OR 'recreational park'/exp OR 'catering service'/exp OR 'restaurant'/exp OR 'sport facility'/exp OR 'city planning'/exp OR (obesogenic* OR 'city planning' OR 'urban planning' OR 'environmental planning' OR (neigh* NEAR/6 planning) OR ((urban OR neigh* OR environment*) NEAR/6 design*) OR "neighbourhood characteristic*" OR "neighborhood characteristic*" OR 'urban form' OR 'sprawl' OR 'zoning' OR 'residential location*' OR (environment* NEAR/6 ('built' OR food* OR 'physical activit*' OR 'physical inactiv*' OR 'PA' OR 'walking' OR 'cycling' OR 'workplace' OR 'green' OR 'natural' OR 'injustice' OR 'design*')) OR 'objective environment*' OR 'measured environment*' OR 'exposome' OR 'parks' OR 'green space*' OR 'green infrastructure*' OR 'natural space*' OR 'natural infrastructure*' OR 'food desert*' OR 'food services' OR restaurant* OR supermarket* OR (facilit* NEAR/6 (sport* OR 'physical activity' OR 'PA' OR 'recreational' OR 'exercise')) OR ((outlet* OR 'supply') NEAR/6 ('food' OR 'fastfood' OR takeaway* OR 'take-away*')) OR 'food deliver*' OR 'convenience store*' OR 'takeaway food' OR 'take-away food' OR 'food store*' OR 'nutrient densit*' OR 'walkability' OR 'walkable' OR 'driveability' OR 'driveable' OR 'job densit*' OR 'population densit*' OR 'intersection' OR 'land use mix' OR 'land-use mix' OR 'public transport*' OR sidewalk* OR 'green space*' OR playground* OR play-ground* OR bikeab* OR cycleab* OR 'pedestrian' OR 'cycle path*' OR cyclepath*):ti,ab,kw	244,849
#4	'spatial analysis'/exp OR 'healthy food access'/exp OR 'geographic information system'/exp OR (exposure* OR proximit* OR densit* OR access* OR distance* OR availabilit* OR geospatial OR 'spatial' OR 'geographic* information system*' OR 'GIS'):ti,ab,kw	4,289,392
#5	#1 AND #2 AND #3 AND #4	9,425
#6	#5 NOT ('conference abstract'/it OR 'conference review'/it)	7,802

Search History Web of Science Core Collection - February 26, 2024

Set	**Web of Science Core Collection Query**	**Results**
#1	TS = (socioeconomic* OR socio-economic* OR "social class*" OR "social status" OR "social determinants of health" OR poverty OR deprivation OR deprived OR income* OR education* OR occupation* OR "residence characteristic*" OR SEP OR SES OR wealth*)	2,621,333
#2	TS = (inequalit* OR equalit* OR difference* OR inequit* OR equit* OR disparit* OR discrepanc* OR contrast* OR dissimilarit* OR disadvant* OR advant* OR injustic* OR justic*)	7,898,122
#3	TS = (obesogenic* OR "city planning" OR "urban planning" OR "environmental planning" OR (neigh* NEAR/6 planning) OR ((urban OR neigh* OR environment*) NEAR/6 design*) OR "neighbourhood characteristic*" OR "neighborhood characteristic*" OR "urban form" OR sprawl OR zoning OR "residential location*" OR (environment* NEAR/6 ("built" OR food* OR "physical activit*" OR "physical inactiv*" OR "PA" OR "walking" OR "cycling" OR "green" OR "workplace" OR "natural" OR "injustice" OR "design*")) OR "objective environment*" OR "measured environment*" OR "exposome" OR "parks" OR "green space*" OR "green infrastructure*" OR "natural space*" OR "natural infrastructure*" OR "food desert*" OR "food services" OR restaurant* OR supermarket* OR (facilit* NEAR/6 (sport* OR "physical activity" OR "PA" OR "recreational" OR exercise)) OR ((outlet* OR "supply") NEAR/6 (food OR fastfood OR takeaway* OR "take-away*")) OR "food deliver*" OR "convenience store*" OR "takeaway food" OR "take-away food" OR "food store*" OR "food desert*" OR "nutrient densit*" OR "walkability" OR "walkable" OR "driveability" OR "driveable" OR "job densit*" OR	1,407,890

	"population densit*" OR "intersection" OR "land use mix" OR "land-use mix" OR "public transport*" OR sidewalk* OR "green space*" OR playground* OR play-ground* OR bikeab* OR cycleab* OR "pedestrian" OR "cycle path*" OR cyclepath*)	
4	TS = (exposure* OR proximit* OR densit* OR access* OR distance* OR availabilit* OR "geospatial" OR "spatial" OR "geographic* information system*" OR "GIS")	7,690,069
5	#1 AND #2 AND #3 AND #4	13,414

Search History Scopus February 26, 2024

Set	**Scopus Query**	**Results**
#1	TITLE-ABS (socioeconomic* OR socio-economic* OR "social class*" OR {social status} OR {social determinants of health} OR {poverty} OR {deprivation} OR {deprived} OR income* OR education* OR occupation* OR "residence characteristic*" OR {SEP} OR {SES} OR wealth*) OR AUTHKEY (socioeconomic* OR socio-economic* OR "social class*" OR {social status} OR {social determinants of health} OR {poverty} OR {deprivation} OR {deprived} OR income* OR education* OR occupation* OR "residence characteristic*" OR {SEP} OR {SES} OR wealth*)	3,538,094
#2	TITLE-ABS (inequalit* OR equalit* OR difference* OR inequit* OR equit* OR disparit* OR discrepanc* OR contrast* OR dissimilarit* OR disadvant* OR advant* OR injustic* OR justic*) OR AUTHKEY (inequalit* OR equalit* OR difference* OR inequit* OR equit* OR disparit* OR discrepanc* OR contrast* OR dissimilarit* OR disadvant* OR advant* OR injustic* OR justic*)	11,224,941
#3	TITLE-ABS (obesogenic* OR "city planning" OR "urban planning" OR "environmental planning" OR (neigh* W/6 planning) OR ((urban OR neigh* OR environment*) W/6 design*) OR "neighbourhood characteristic*" OR "neighborhood characteristic*" OR "urban form" OR sprawl OR zoning OR "residential location*" OR (environment* W/6 ("built" OR food* OR "physical activit*" OR "physical inactiv*" OR "PA" OR "walking" OR "cycling" OR "green" OR "workplace" OR "natural" OR "injustice" OR "design*")) OR "objective environment*" OR "measured environment*" OR "exposome" OR "parks" OR "green space*" OR "green infrastructure*" OR "natural space*" OR "natural infrastructure*" OR "food desert*" OR "food services" OR restaurant* OR supermarket* OR (facilit* W/6 (sport* OR "physical activity" OR "PA" OR "recreational" OR exercise)) OR ((outlet* OR "supply") W/6 (food OR fastfood OR takeaway* OR "take-away*")) OR "food deliver*" OR "convenience store*" OR "takeaway food" OR "take-away food" OR "food store*" OR "food desert*" OR "nutrient densit*" OR "walkability" OR "walkable" OR "driveability" OR "driveable" OR "job densit*" OR "population densit*" OR {intersection} OR "land use mix" OR "land-use mix" OR "public transport*" OR sidewalk* OR "green space*" OR playground* OR play-ground* OR bikeab* OR cycleab* OR {pedestrian} OR "cycle path*" OR cyclepath*) OR AUTHKEY (obesogenic* OR "city planning" OR "urban planning" OR "environmental planning" OR (neigh* W/6 planning) OR ((urban OR neigh* OR environment*) W/6 design*) OR "neighbourhood characteristic*" OR "neighborhood characteristic*" OR "urban form" OR sprawl OR zoning OR "residential location*" OR (environment* W/6 ("built" OR food* OR "physical activit*" OR "physical inactiv*" OR "PA" OR "walking" OR "cycling" OR "green" OR "workplace" OR "natural" OR "injustice" OR "design*")) OR "objective environment*" OR "measured environment*" OR "exposome" OR "parks" OR "green space*" OR "green infrastructure*" OR "natural space*" OR "natural infrastructure*" OR "food desert*" OR "food services" OR restaurant* OR supermarket* OR (facilit* W/6 (sport* OR "physical activity" OR "PA" OR "recreational" OR exercise)) OR	1,081,762

	((outlet* OR "supply") W/6 (food OR fastfood OR takeaway* OR "take-away*")) OR "food deliver*" OR "convenience store*" OR "takeaway food" OR "take-away food" OR "food store*" OR "food desert*" OR "nutrient densit*" OR "walkability" OR "walkable" OR "driveability" OR "driveable" OR "job densit*" OR "population densit*" OR {intersection} OR "land use mix" OR "land-use mix" OR "public transport*" OR sidewalk* OR "green space*" OR playground* OR play-ground* OR bikeab* OR cycleab* OR {pedestrian} OR "cycle path*" OR cyclepath*)	
4	TITLE-ABS (exposure* OR proximit* OR densit* OR access* OR distance* OR availabilit* OR {geospatial} OR {spatial} OR "geographic* information system*" OR {GIS}) OR AUTHKEY (exposure* OR proximit* OR densit* OR access* OR distance* OR availabilit* OR {geospatial} OR {spatial} OR "geographic* information system*" OR {GIS})	10,384,879
5	#1 AND #2 AND #3 AND #4	12,246
