# Supplementary material for: Socioeconomic inequalities in exposure to neighbourhood environments for physical activity: a systematic review
Source: Int J Behav Nutr Phys Act. 2026 Apr 9;23:58. doi: 10.1186/s12966-026-01912-1 (PMC13231669; doi:10.1186/s12966-026-01912-1)
Supplement: Supplementary file 3 — Supplementary Material 3. [file 12966_2026_1912_MOESM3_ESM.pdf]

Information included in data extraction form:

Study ID
Country of data collection
Region(s) of data collection (e.g. which states/cities)
Study setting (urban/rural)
Unit of analysis
Number of units
Age category
Year(s) of data collection SEP variable(s)
Income operationalisation
Education operationalisation
Occupation/employment operationalisation
Other SEP-indicator operationalisation
If index used: name
Income included in index (yes/no)
Education included in index (yes/no)
Occupation included in index (yes/no)
Other indicators included in index (yes/no)
Index scale
Index interpretation
Year(s) of data collection environmental exposures
Operationalisation PA environment type
Analysis method
If regression: specify X and Y
Conclusion
Quality
Notes
