# Supplementary material for: Socioeconomic inequalities in exposure to neighbourhood environments for physical activity: a systematic review
Source: Int J Behav Nutr Phys Act. 2026 Apr 9;23:58. doi: 10.1186/s12966-026-01912-1 (PMC13231669; doi:10.1186/s12966-026-01912-1)
Supplement: Supplementary file 4 — Supplementary Material 4. [file 12966_2026_1912_MOESM4_ESM.pdf]

1. Introduction: Were the aims/objectives of the study clear?
2. Methods: Was the sample frame taken from an appropriate population or ecological (e.g. census tracts) base so that it closely represented the target/reference population or ecological context under investigation?
3. Methods: Was SEP measured with more than 1 single indicator?
4. Methods: Were SEP-variables adequately described or do the authors refer to other paper(s) where the variables are adequately described? Note: Indicate 'partly' if more SEP-indicators were measured, of which some are adequately described/referred to description, and some were not
5. Methods: Were environmental variables adequately described or do the authors refer to other paper(s) where the variables are adequately described? Note: Indicate 'partly' if more environmental indicators were measured, of which some are adequately described/referred to description, and some were not
6. Methods: Were the SEP- and environmental exposures measured within the same year (or one year before/after)? Note: Indicate 'partly' if more SEP-indicators were measured, of which some were measured within the same year or one year before/after, and some were not
7. Methods: Was SEP analysed at individual level? Note: Indicate 'partly' if more SEP-indicators were measured, from which some were measured on individual level and some on a larger level
8. Methods: Was missing data handling adequately described? E.g. Did the authors indicate that missing data was removed, or missing data was imputated, or something else?
9. Methods: Were the methods (including statistical methods) sufficiently described to enable them to be repeated?
10. Results: Were the basic data adequately described? E.g. Is their descriptives table understandable, is basic information on participants described, e.t.c.
11. Results: If appropriate, was information about non-responders described? Note: For ecological studies, this can be answered with ‘not relevant’. Papers that use secondary data should also provide information about non-responders. Indicate 'no' if no information on non-response was provided.
12. Discussion: Were the limitations of the study discussed?
13. Other: Were funding sources and conflicts of interest disclosed? Note: Indicate 'partly' if either only the conflicts of interest or the funding source is disclosed.
14. Other: Was ethical approval or consent of participants attained?

Question	Answer	Points	Relevant q	Overall quality		Options
1. Introduction: Were the aims/objectives of the study clear?						yes
2. Methods: Was the sample frame taken from an appropriate population or ecological (e.g. census tracts) base so that it closely represented the target/reference population or						partly
3. Methods: Was SEP measured with more than 1 single indicator?						no/notstated
4. Methods: Were SEP-variables adequately described or do the authors refer to other paper(s) where the variables are adequately described?						not relevant
5. Methods: Were environmental variables adequately described or do the authors refer to other paper(s) where the variables are adequately described?						
6. Methods: Were the SEP- and environmental exposures measured within the same year (or one year before/after)?						
7. Methods: Was SEP analysed at individual level?						
8. Methods: Was missing data handling adequately described?						
9. Methods: Were the methods (including statistical methods) sufficiently described to enable them to be repeated?						
10. Results: Were the basic data adequately described?						
11. Results: If appropriate, was information about non-responders described?						
12. Discussion: Were the limitations of the study discussed?						
13. Other: Were funding sources and conflicts of interest disclosed?						
14. Other: Was ethical approval or consent of participants attained?						
		0	0	#DIV/0!		
