# Supplementary figures and images for: Socioeconomic inequalities in exposure to neighbourhood environments for physical activity: a systematic review
Source: Int J Behav Nutr Phys Act. 2026 Apr 9;23:58. doi: 10.1186/s12966-026-01912-1 (PMC13231669; doi:10.1186/s12966-026-01912-1)

Figure S1

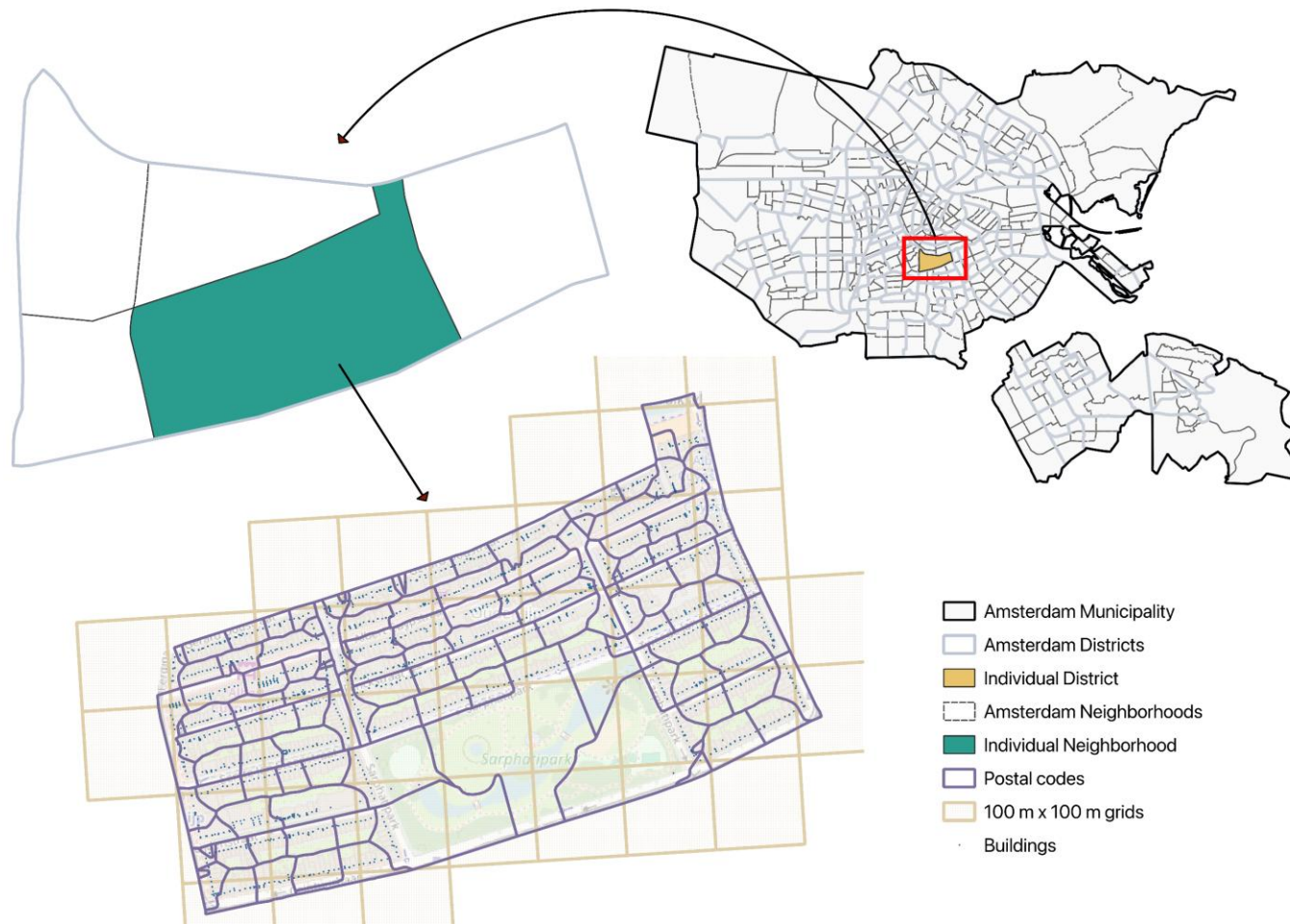

Visual illustration of commonly used units and buffer sizes

Supplement: Supplementary file 5 — Supplementary Material 5. [file 12966_2026_1912_MOESM5_ESM.pdf]
