# Supplementary material for: Socioeconomic inequalities in exposure to neighbourhood environments for physical activity: a systematic review
Source: Int J Behav Nutr Phys Act. 2026 Apr 9;23:58. doi: 10.1186/s12966-026-01912-1 (PMC13231669; doi:10.1186/s12966-026-01912-1)
Supplement: Supplementary file 6 — Supplementary Material 6. [file 12966_2026_1912_MOESM6_ESM.pdf]

Table S1: Extended descriptive information on included studies

Author, year of publication	Country: region(s) (setting)	Operationalisation SEP indicator(s)	Operationalisation environmental PA resource(s) studied	Geographic unit(s) of analysis (n)	Analysis method(s)	Quality
Aamodt et al., 2023	Finland: Espoo; Norway: Stavanger; Sweden: Täby (urban)	Proportion of the population that holds at least a bachelor's degree<br><br>Median disposable household income (in Euros) available to households in a municipality	Distance to green space defined as the dissolved area of parks, forests, and cemeteries<br><br>Proportion of green space<br><br>Area of green space per inhabitant	Neighbourhood (7, 9, 38)	Bivariate correlation	Good
Abercrombie et al., 2008	USA: Baltimore-Washington DC (urban)	Median block group income, recoded into equal tertiles: low ($0 –$41,763), middle ($41,764 –$63,850), and high ($63,851 and above)	Count of public parks in block group, recoded into five categories: 0, 1, 2, 3, or 4 or more parks in the block group<br><br>Park size in block group, divided into four categories: (1) up to 4.99 acres, (2) 5 to 29.99 acres, (3) 30 to 49.99 acres, and (4) 50 acres or more.<br><br>Count of private recreation facilities, recoded into five categories: 0, 1, 2, 3, or 4 or more facilities in the block group	Block group (833) and census tract (833)	ANCOVA	Fair
Agay-Shay et al., 2019	Israel: Tel Aviv (urban)	SEP-index<br><br>3 categories of maternal education	NDVI	Individual/household (300m buffer around home) (73221)	Summary statistics (e.g. means/medians/proportions)	Good
Akaraci et al., 2021	Australia: Sydney (urban)	SEIFA-IRSAD	Ratio of total land surface which is identified as green space in the SA2 of mother's residential address at the time of birth. Green space was categorised into 3 categories: 0%-20%, 20%-40%, and >=40%	Statistical Area Level 2 (approx. 200)	Summary statistics (e.g. means/medians/proportions)	Good
Alderton et al., 2022	Australia: Adelaide (urban)	Maternal highest level of schooling and highest post-school qualification by caregiver<br><br>Quintiles of SEIFA-IRSD	% people living within 400m/800m from 'child friendly' public open space with both a playground and a toilet nearby	Individual/household (400 and 800m buffer around home) (199200)	Summary statistics (e.g. means/medians/proportions)	Good

Apparicio et al., 2016	Canada: Island of Montreal (urban)	% population with low income before tax per city block	Mean vegetation within city block, within 250 m of city block, and within 500 m of city block	City blocks (10210)	Independent t-test	Fair
Astell-Burt et al., 2014	Australia: Sydney, Melbourne, Brisbane, Perth, Adelaide (urban)	5 categories of % of SA1 population living on a low income	Mean area of green space in m2, And odds of having ≥10%, ≥20%, and ≥40% green space in neighbourhood	Statistical Area Level 1 (28626)	Multilevel logistic regression	Fair
Aznarez et al., 2023	Spain: Vitoria-Gasteiz (urban)	% of residents with high educational attainment by neighbourhood over the total population	% vegetation cover per neighbourhood	Neighbourhood (28)	Simple linear regression	Fair
Badland et al., 2010	New Zealand: Waitakere City (urban)	2006 NZDep	Sum score of sports activities available in public open spaces in or proximal to neighbourhoods	Clusters of 5 contiguous mesh-blocks (geographic units of approximately 100 households) (69)	ANOVA	Fair
Banzhaf et al., 2017	Chile: Cerro Navia, La Florida, and Vitacura (urban)	3 categories of income level (1 municipality per level)	m² green spaces per inhabitant	Municipality (3)	Summary statistics (e.g. means/medians/proportions)	Poor
Bao et al., 2023	UK: Greater London, Northeast England, Northwest England, Yorkshire and the Humber, West Midlands, East Midlands, East England, Southwest England, and Southeast England (urban)	Housing barriers: The physical and financial accessibility of housing and local services<br><br>low achievement and skill levels within the local population	Proximity in km to nearest green space using urban zip codes	Lower Layer Super Output Areas (32844)	multi-scale geographically weighted regression	Fair
Baró et al.,, 2021	Spain: Barcelona (urban)	Gross household disposable income index	Number of public green spaces/playgrounds in 300m around school main entrance	School (324)	Bivariate correlation	Good

		% of population over 16 years old with primary education or no studies.				
Barton et al., 2017	USA: New York City (urban)	Neighbourhood median household income per census tract<br><br>Change in neighbourhood median household income over time	Bus stops and subway stops per square mile in 2000<br><br>Bus stops and subway stops per square mile in 2000<br><br>Change in bus stops and change in subway stops per square mile over time	Census tract (2073)	Multilevel linear regression<br><br>Cross-sectional spatial lag regression	Fair
Bereitschaft et al., 2017	USA: Charlotte, Pittsburgh, Portland (urban)	% of households below the poverty level per block group<br><br>% of residents (aged 25+ years) with no college education per block group<br><br>% of workers employed in service occupations (i.e. healthcare support, protective services, food preparation and serving, building and grounds cleaning, and personal care and service per block group	Walk Score	Block groups (463)	Simple logistic regression	Fair
Bezold et al., 2018	USA (urban and rural)	Household income (individual level), dichotomized into >=$75,000 and < $75,000.<br><br>Census tract median household income<br><br>Census tract % paternal college educated	NDVI	Individual/household (1,250m buffer around home) (9385) and census tract	Summary statistics (e.g. means/medians/proportions)	Good
Billaudeau et al., 2011	France: Paris and surroundings (urban)	Area median income within 500, 1000, 2500, and 5000m	Total number of sports facilities within 500m straight line of the census blocks	Census blocks (2608)	Poisson regression based on GEE	Fair
Botticello et al., 2015	USA, New Jersey (urban)	Home value (participant's census tract mean housing value)	Sum of proportions of all natural developed and developed land cover types, dichotomized at the 75th percentile - low (under 75th) and high (over 75th) open space)<br><br>Residential density (proportion of residential land use, 0 to 1)	Community(5 mile/8 km buffer around home)(503)	Chi-square test<br><br>ANOVA	Fair

			Land use mix (weighted index of the proportions of residential, commercial, industrial, recreational and mixed urban use, 0 to 1),<br><br>Destination density (tertile scores of the aggregate count of religious, entertainment, landmark and retail locations, low, moderate and high destination density)			
Brown et al., 2018	USA: Miami-Dade County (urban)	3 levels of neighbourhood median household income	NDVI	Census block (36563)	ANOVA	Fair
Buckland et al., 2022	UK: Birmingham; Belgium: Brussels; Italy: Milan; Czech Republic: Prague; Sweden: Stockholm (urban)	Average annual income by district	% of residences that had access to at least one green space within a 300-m threshold along the footpath network	Districts	Simple linear regression	Fair
Buszkiewicz et al., 2022	USA: Seattle (urban)	Education attainment divided in three levels	Residential density at 800m<br><br>Road intersection density at 800m	Individual/household (800m buffer around home) (819)	Summary statistics (e.g. means/medians/proportions)	Fair
Carrier et al., 2016	Canada: Island of Montreal (urban)	Quintiles of % low-income population per city block	Mean park area in hectares<br><br>Mean % vegetation cover	City blocks (10290)	Independent t-test<br>Spatial lag regression	Fair
Carroll et al., 2023	Australia: Sydney, Melbourne, Brisbane, Adelaide, Perth, Hobart, Darwin, Canberra (urban & rural)	IRSD	Count of amenities within a1000m road-network buffer centred on each participant's place of residence (libraries, petrol stations, hospitality, retail (excluding supermarkets), and entertainment venues)	Individual/household (1,000m buffer around home) (1675, 1735, 1383, 1067, 1173, 533, 718, 1050)	ANOVA	Good
Casey et al., 2017	USA: Entire USA (urban)	Modified version of Index of Concentration at the Extremes for income<br><br>% renter-occupied housing units	NDVI<br><br>Change in NDVI 2001 - 2011	Census tract (59483)	Bivariate correlation<br><br>Spatial error linear regression	Good

Cereijo et al., 2022	Spain: Madrid (urban)	Neighbourhood SEP-index	Tertiles of number of exercise facilities within 1,000m street network buffer around each portal	Individual/household (1,000m buffer around home) (186071, 237381)	Summary statistics (e.g. means/medians/proportions)	Good
Cereijo et al., 2023	Spain: Madrid (urban)	Neighbourhood SEP-index	The count of exercise facilities within a 1000 m street network buffer from each residential building entrance	Individual/household (1,000m buffer around home) (1214281)	Summary statistics (e.g. means/medians/proportions)	Good
Chandrabose et al., 2022	Australia: All states (urban & rural)	Tertiles of IRSD	Intersection density<br><br>Residential density<br><br>Destination density<br><br>Neighbourhood walkability index, containing residential density, intersection density, and destination density	Individual/household (1,000m buffer around home) (3590)	Summary statistics (e.g. means/medians/proportions)	Good
Chaney et al., 2019	USA: Utah (urban)	Median household income<br><br>Poverty rate<br><br>Educational attainment<br><br>Housing value	Trail density<br><br>Trailhead access within municipality's activity space	Municipality (33)	Simple linear regression	Fair
Chaparro et al., 2018	England: Wales, Scotland, London, rest of the regions are not stated (urban & rural)	The Carstairs index	Exposure to green space, defined as the percent of (public) green space in the Census Area Statistic ward of residence	Census area statistic ward (4929)	Simple linear regression	Good
Cheruvalath et al., 2022	USA: Milwaukee County, Wisconsin (urban & rural)	Area deprivation index	Proximity to public parks<br><br>NDVI	Census block group (5870)	Bivariate correlation	Good
Choi et al., 2020	USA: Seattle, Salt Lake City, Fresno: Minneapolos, Kansas City, Grand Rapids,	Median household income on block group level	Density of street greenery in a block group<br><br>Acreage of S–L parks (<20 acres) within 0.5 mile network distance	Block groups (14285)	Multilevel linear regression	Fair

	Houston, Raleigh, Brandenton, Boston, Providence, Buffalo (urban)	% of people with ≥ bachelor's degree on block group level	Acreage of XL parks (≥20 acres) within 0.5 mile network distance			
Choi et al., 2022	Canada: Toronto (urban)	Tertiles of neighbourhood income level, depending on the share of after-tax low-income residents	% of land covered by open green space<br><br>Walk Score	Neighbourhood (140)	Summary statistics (e.g. means/medians/proportions)	Fair
Christie et al., 2023	Canada: 31 cities (urban)	Median neighbourhood home values	The 2016 Canadian Active Living Environments index (walkability)	Dissemination areas (33026)	Multilevel linear regression	Good
Chuang et al., 2023	New Zealand: Auckland (urban)	Dichotomisation of top (with) and bottom (without) public housing neighbourhoods	Mean and median Euclidean distance from home location cluster centroid to nearest park centroid<br><br>Network mean and median distance from home location cluster centroid to nearest park<br><br>Mean Euclidean distance from 300mx300m grid centroid to nearest park centroid<br><br>Network mean and median distance from 300mx300m grid centroid to nearest park	Individual/household (1000) and 300x300m grid centroids (3070)	Mann-Whitney U/Kruskall Wallis	Fair
Clennin et al., 2019	USA: South Carolina (urban)	Quartiles of neighbourhood socioeconomic deprivation	Physical Activity Resources Assessment	Census tract (42)	ANOVA	Good
Cohen et al., 2013	USA: Philadelphia, Columbus, Chapel Hill/Durham, Albuquerque (urban)	Dichotomisation of low/high income neighbourhoods	Average park acres per income category	Neighbourhood's parks (24)	Independent t-test	Fair
Cohen-Cline et al., 2015	USA: Washington (urban)	Singh Index<br><br>Dichotomisation of income	NDVI	Individual/household (1,000m buffer around home)(4338)	Summary statistics (e.g. means/medians/proportions)	Fair
Collyer et al., 2022	Australia: Perth and Peel metropolitan regions of Western Australia (urban)	SEIFA-IRSD	Total park count<br><br>Count of pocket-small and medium-large parks	Statistical Area Level 1 (260)	Summary statistics (e.g. means/media	Good

			Distance to closest park (100m)<br><br>High traffic roads in SA1<br><br>Street connectivity (Mean count of ≥3 way intersections per SA1s)<br><br>Cul-de-sacs (one-way nodes) per increase of 10<br><br>Mean distance to closest standard transport stop (km)<br><br>Mean count of school bus stops and standard within 1.6 km radius<br><br>Mean count of rail stations within 5 km radius		ns/proportions)	
Comer et al., 2008	USA: Oklahoma City (urban)	Per capita income on block group level	Minimum distance to park<br><br>Gravity potential for park availability	Block groups (620)	Bivariate correlation	Fair
Conderino et al., 2021	USA: Midwest, Northeast, South, and West (urban)	Median household income for the geographic area, categorized in tertiles as low, middle, and high	2019 Walk Score	City/town (500) and census tract (28130)	Summary statistics (e.g. means/medians/proportions)<br><br>Simple linear regression<br><br>Bivariate linear model	Fair
Cote-Lussier et al., 2015	Canada: Quebec (urban)	% low-income households in neighbourhood	NDVI	Individual/household (500m buffer around home) (2120)	Bivariate correlation	Good
Cottagiri et al., 2022	Canada: Victoria, Vancouver, Surrey, Calgary, Winnipeg, Ottawa, Hamilton, Montreal,	3 levels of individual household income	NDVI	500m buffer around postal codes(26811)	Summary statistics (e.g. means/medians/proportions)	Good

	Sherbrooke, Halifax:, St. John's (urban)					
Coutinho et al., 2023	Norway: Oslo (urban)	Neighbourhood deprivation composite score	Mean number of green spaces per neighbourhood deprivation tertile<br><br>Total area of green spaces<br><br>Mean number of indoor recreational facilities per neighbourhood deprivation tertile<br><br>Number of recreational facilities<br><br>Mean number of small and large outdoor recreational facilities per neighbourhood deprivation tertile<br><br>Mean number of public transport stops per neighbourhood deprivation tertile	sub-district level defined by administrative boundaries (97)	MANCOVA<br><br>Bivariate correlation<br><br>Partial correlation<br><br>ANCOVA	Good
Cowie et al., 2016	Australia: Sydney (urban)	IRSD	Walkability index	Census Collection District (5858)	Prevalence rates	Good
Cradock et al., 2005	USA: Boston (urban)	% people aged <18 living in poverty per block group<br><br>% of adults aged >25 years without a high school degree per block group	Straight-line distance in m from the centroid of each block group to nearest playground	Census block groups (591)	GEE linear regression<br><br>Bivariate linear model	Fair
Crawford et al., 2008	Australia: Melbourne (urban)	SEIFA-IRSAD	Presence of trees providing shade in neighbourhood (%)<br><br>Presence of water feature (river, creek) in neighbourhood (%)<br><br>Presence of walking paths in neighbourhood (%)<br><br>Presence of cycling paths in neighbourhood (%)<br><br>Presence of lighting along path in neighbourhood (%)<br><br>Mean number of playgrounds	Public open space locations (1497)	ANOVA<br><br>Chi-square test	Poor

Creatore et al., 2016	Canada: London, Ottawa, Hamilton, Toronto, and surrounding communities (urban & rural)	% of population living below the low-income cutoff<br><br>Population aged ≥20 y with high school education or less, %<br><br>Unemployment, %<br><br>% of households with household income (adjusted for household size) falling below a fixed threshold.<br><br>Population age 25-64 years with high school education or less, %	Walkability index	Dissemination areas composed of several adjacent city blocks (8777)	Summary statistics (e.g. means/medians/proportions)	Good
Csomós et al., 2020	Hungary: Debrecen (urban)	Neighbourhood mean income per year<br><br>Neighbourhood % of college graduates	5 categories of distance to green space	Neighbourhood	Summary statistics (e.g. means/medians/proportions)	Poor
Csomós et al., 2021	Hungary: Debrecen, Kecskemet, and Szeged (urban)	Average income (% of the average income of the total population)<br><br>Share of people having higher education degree	Demand–supply composite index for greenness	100 x 100m grid cells (3093, 2151, and 2540)	Summary statistics (e.g. means/medians/proportions)	Fair
Curtis et al., 2024	USA: Utah (urban)	Neighbourhood SES composite	Amount of green and natural amenities in each tract in 1990<br><br>Time*nSES interaction for green and natural amenities in each tract<br><br>Time trend in amount of green and natural amenities<br><br>Walkability index<br><br>Time*nSES interaction for walkability<br><br>Time trend in amount of walkability	Census tract (436)	Multilevel linear regression	Good

Cutts et al., 2009	USA: Phoenix (urban)	% of the block group population below the poverty level<br><br>% of the census block group population with no education beyond high school	proximity to parks using a 0.25 mi (0.4 km) buffer and a symmetrically mapped census block group data.<br><br>Walkability index	Census block groups (1046)	Independent t-test	Fair
Dahmann et al., 2010	USA: Los Angeles (urban & rural)	Median Household Income	Number of recreational courses offered per thousand in municipality	Municipality (71)	Simple linear regression	Poor
Darcy et al., 2022	Australia: Queensland (urban & rural)	SEIFA	SPACES audit tool	Suburb (25)	Bivariate correlation	Fair
Davis et al., 2012	USA: Chicago (urban)	Census tract level median household income	Mean distance to public open space from the centroid of each census block within census tracts	Census tract (822)	Bivariate correlation	Fair
DelaBarerra et al., 2019	Chile: Santiago de Chile (urban)	Household income on census tract level	NDVI	Neighbourhood (7)	Bivariate correlation	Poor
DelaBarrera et al., 2016	Chile: Santiago de Chile (urban)	Municipality level household income	% vegetation cover per municipality<br><br>Amount of green space per inhabitant per m2	Municipality (3)	Summary statistics (e.g. means/medians/proportions)	Poor
Dobbs et al., 2023	Chile: Metropolitan Region of Santiago (urban & rural)	Municipality category of social priority, indicating social status	Number of green spaces<br><br>Mean size green spaces (ha)<br><br>Mean distance among green spaces (m)<br><br>Square meter of green space per inhabitant<br><br>Quality of green space	Municipality (52)	MANOVA	Fair
Doiron et al., 2020	Canada: Toronto, Montreal, Vancouver (urban)	Material and Social Deprivation Index<br><br>Canadian Active Living Environments index	NDVI	Postal codes (41200)	Summary statistics (e.g. means/medians/proportions)	Good
Duncan et al., 2012	USA: Boston (urban)	% of families living in poverty per census tract	Walk Score	Census tract (167)	Bivariate correlation<br><br>Simple linear regression	Good

Edwards et al., 2011	USA: North Carolina (urban & rural)	County poverty rate<br><br>County level median household income<br><br>% of residents over 25 not completing high school per county<br><br>Socioeconomic disadvantage composite measure	Public Recreation Index	County (100)	Independent t-test	Fair
Eime et al., 2017	Australia: Victoria (urban & rural)	SEIFA IRSAD	Mean number of sports fields/courts per 1,000 residents	Local government areas (79)	Bivariate correlation	Fair
Evans et al., 2012	Italy: Forlì; Lithuania: Vilnius; Portugal: Ferreira do Alentejo; Germany: Bonn; Switzerland: Geneva; France: Angers; Slovakia: Bratislava; Hungary: Budapest (urban & rural)	After tax income reported in 6 category ranges (e.g., for EU countries 1 = <500 ER monthly income, 6 => 2500 monthly income).	Open green space - 4 item composite consisting of observer ratings (1 = no green space, 2 = green space) for access to 4 types of greenery in the immediate neighbourhood (streets, public grounds, accessible private grounds, vegetation on and around buildings)	Individual/household (1184)	Zero order correlation matrix	Fair
Farkas et al., 2022	Hungary: Budapest (urban)	Average income per capita in Euros<br><br>Proportion of people having higher education degree (% of the total population)	Demand–supply composite index for greenness	100 x 100m Grid cells (23459)	Summary statistics (e.g. means/medians/proportions)	Good
Fazli et al., 2019	Canada: Southern Ontario (urban & rural)	Area-level income (operationalized as quintiles, adjusted for household and community size)	Walkability index (validated index based on the postal code of the participants, based on population density, residential density, street connectivity and availability of retail outlets, services, and schools within walking distance of people's homes using an 800m geographic boundary around the centre of each residential area)	Individual/household (1128181)	Summary statistics (e.g. means/medians/proportions)	Fair
Ferguson et al., 2018	UK: Bradford (urban)	Household deprivation	% of each LSOA that had sufficient access to a public greenspace based on 300m Euclidean distance	Lower Layer Super Output Areas (218)	Bivariate correlation<br><br>Partial correlation	Fair

			Dichotomisation of high and low greenspace provision (more/less 50% of the area of the LSOA with good access)		Mann-Whitney U test	
Fernández et al., 2016	Chile: Santiago de Chile (urban)	Household Wealth Index	NDVI	Census block	Bivariate correlation	Fair
Fian et al., 2023	Austria: Forli, Vilnius, Ferreira do Alentejo, Bonn, Geneva, Angers, Bratislava, Budapest (urban & rural)	Household income<br><br>Education<br><br>Work status	River present within 1 km of participant's home yes/no<br><br>Lake present within 1 km of participant's home yes/no<br><br>NDVI	Individual/household (1,000m buffer around home) (2258)	Simple logistic regression<br><br>Simple linear regression	Good
Field et al., 2004	New Zealand: Auckland (urban)	New Zealand Index of Deprivation	% of urban green space service area within the area of SA1.<br><br>Green space accessibility taking into account distance decay	Statistical Area Level 1 (8770)	Bivariate correlation<br><br>Simple linear regression<br><br>Spatial lag regression	Good
Field et al., 2024	USA: Ohio, New York, Indiana, Illinois, California, Utah, Pennsylvania (urban)	Household income and size relative to US poverty level, categorised in 3 categories: <130%, 130%-350%, and >350%<br><br>Education level divided into 4 categories: High school or less, some college, college graduate, graduate degree	National Walkability Index	Individual/household (7500 and 9148)	Summary statistics (e.g. means/medians/proportions)	Good
Flacke et al., 2016	Germany: Dortmund (urban & rural)	Sum of inhabitants receiving either unemployment benefits or social welfare aids as a % of the total population	share of green areas (parks and forests, > 1ha, including areas in 400 m buffer surrounding neighbourhood), as a % of total area of the neighbourhood	Neighbourhood (170)	Bivariate correlation	Good
Franzini et al., 2010	USA: Birmingham (AL): Houston (TX): Los Angeles (CA) (urban)	Neighbourhood poverty rate categorized as low (<10%), medium (10-20%) and high (>20%)	Residential vs commercial density (primarily residential vs commercial/industrial)<br><br>High residential density (prevalence of residential units that were not stand-alone houses or duplexes, categorized in high vs low density)	Block-faces (632)	Simple logistic regression<br><br>Simple linear regression	Fair

			Destinations (list of 70 types of commercial and institutional establishments and public transportation)<br><br>Recreational facilities (presence of recreational facilities such as parks, playgrounds, or playing fields (one item, binary))			
Fraser et al., 2024	USA: Boston (urban)	Median Household Income	Median distance to parks (green spaces + squares + fountains + playgrounds)	Buildings (85592)	Simple linear regression	Fair
Frey et al., 2017	USA: Washington D.C. (urban)	% of owner-occupied housing	% of census tract classified as park	Census tract (175, 46, and 129)	Simple linear regression<br><br>Spatial error model	Fair
Fuller et al., 2013	Canada: Montreal (urban)	Quartiles of household incomes	Access to bicycle share program<br><br>Distance to subway	Individual/household (6495)	Multilevel negative binomial regression	Good
Fuller et al., 2017	Canada: Calgary, Halifax, Moncton, Montreal, Saskatoon, Toronto, Vancouver, and Victoria (urban)	Quintiles of individual median income	Bike score and bike lane score	Census tract (1282)	Simple linear regression	Fair
Garrison et al., 2019	USA: New York City (urban)	% of households in poverty	% of surface area covered by parks	Census block group (6211)	Simple linear regression	Fair
Giles-Corti et al., 2002	Australia: Perth (urban)	Social advantage index	Top quartile of spatial access to attractive public open space/river/sport and recreations centres/gyms/swimming pools/tennis courts/golf courses	Individual/household (1803)	Simple logistic regression	Fair
Gilliland et al., 2006	Canada: London, Ontario (urban)	Social distress index	Number of recreational opportunities per square kilometre.	Dissemination area (22)	Bivariate correlation	Fair
Gonzales-Inca et al., 2022	Finland: Entire urban Finland	Neighbourhood Socioeconomic Disadvantage Index<br><br>3 categories of education level<br><br>Dichotomisation of employment status	Sum of the % of built-up areas mixed with vegetation, agricultural areas, forest areas, shrub and grass areas, and wetlands within 100m radius around individuals' home<br><br>NDVI	Individual/household (100m buffer around home) (14424, 14271, 14375)	Summary statistics (e.g. means/medians/proportions)	Good

Gordon-Larsen et al., 2006	USA: USA wide	% of population with college degree or higher	≥1 public facilities (public beach, pools, tennis courts, recreation centres)/ ≥ 1 outdoor facilities (sporting and recreational camps, swimming pools)/public fee facilities (physical fitness facilities, bicycle rental, public golf courses)/dance studio, basketball instruction, martial arts/athletic club, gymnasium, tennis club, basketball club in block group	Block group (42857)	Simple logistic regression	Fair
Gullón et al., 2017	Spain: Madrid (urban)	Composite SES index	Walkability index	Census section (2415)	Multilevel linear regression	Fair
Gullon et al., 2021	USA: North Carolina, South Carolina, Georgia, Alabama, Mississippi, Tennessee, Arkansas, and Louisiana (urban & rural)	Household income: low socioeconomic position defined as low annual household income (<$35,000)<br><br>Education level: low socioeconomic position defined as low education (less than high school)	Green land cover (km2) derived from satellite imagery or aerial photography<br><br>Walking destinations (include both potential destinations for daily living and those judged as amenities adding to pedestrian interest and overall walkability)<br>Physical activity facilities: included a variety of venues for physical activity or fitness, such as indoor gyms, that have been previously associated with both gym membership and objective physical activity	Individual/household (1,000 m buffer around home) (20808)	Summary statistics (e.g. means/medians/proportions)	Good
Gullón et al., 2023	Spain: Madrid (urban)	Socioeconomic deprivation index	NDVI	Individual/household (200, 300, 500, and 1,000m buffer around home) (437513)	Summary statistics (e.g. means/medians/proportions)	Good
Gunn et al., 2022	Australia: Melbourne (urban)	Index of Relative Socioeconomic Disadvantage	Street connectivity (Number of intersections of three or more streets within 1600 m)<br><br>Dwelling density (The total number of dwellings divided by the area in hectares)<br><br>Daily living score (For each residential address, one point was assigned for having any supermarket, any public transit stops and/or any convenience store, petrol station or newsagent within 1600 m)	Suburb	Summary statistics (e.g. means/medians/proportions)	Good

			Distance to frequent transit stops (Distance to closet bus stop and distance to any public transit stop with a service frequency of at least 30 min between the hours of 7 am and 7pm)<br><br>Walkability index (Composite of z-scores of street connectivity, dwelling density, and daily living score)			
Guo et al., 2017	Hong Kong: Hong Kong (urban)	Neighbourhood poverty (% of the local population living below the official poverty line defined by the government as 50% of the median household income by household size)	Number of gardens/parks per 1000 of the population (container measure)<br><br>Distance to gardens/parks (median road-network distance measure)<br><br>Demand-supply buffer for gardens/parks per 1000 of the population (2SFCA measure)<br><br>Number of indoor games/halls/recreation centres/sports centres per 1000 of the population (container measure)<br><br>Distance to indoor games/halls/recreation centres/sports centres (median road-network distance measure)<br><br>Demand-supply buffer for indoor games/halls/recreation centres/sports centres per 1000 of the population (2SFCA measure)<br><br>Number of sports grounds per 1000 of the population (container measure)<br><br>Distance to sports grounds (median road-network distance measure)<br><br>Demand-supply buffer for sports grounds per 1000 of the population (2SFCA measure)<br><br>Number of public transport stations/stops per 1000 of the population (container measure)	Large street blocks (1629)	Bivariate correlation	Good

			Distance to public transport stations/stops (median road-network distance measure)<br><br>Demand-supply buffer for public transport stations/stops per 1000 of the population (2SFCA measure)			
Hannon et al., 2006	USA: Boston (urban)	Neighbourhood median household income ($)	Recreational facility access (ratio of youths per recreational facility)	Neighbourhood (12)	Summary statistics (e.g. means/medians/proportions)	Fair
Harris et al., 2015	USA: Entire USA (urban & rural)	4 categories of median annual household income per block group	Dichotomisation of living in block group with/without a park within one-half mile<br><br>Three levels of access to parks (based on average number of parks within one-half mile of the blocks in the block group)	Block groups (216013)	Summary statistics (e.g. means/medians/proportions)<br><br>Simple logistic regression	Fair
Hart et al., 2022	USA: Kentucky (urban)	Individual income (categorized in 4 levels - less than $20.000, $20.000-$64.999, $65.000-$124.000, over $125.000)<br><br>Individual level of education (categorized in 3 levels - high school or less, 2-4 year degree, Master or Doctorate)	NDVI	Individual/household (300m buffer around home) (175)	Summary statistics (e.g. means/medians/proportions)	Fair
Heo et al., 2023	USA: New Haven, Baltimore, Durham (urban)	% of block group population with household income ≤ twice the federal poverty level<br><br>% of block group ≥25years with less than high school education	% of land covered by vegetation or greenspace (trees, lawns, gardens, crop land, forests, wetlands)<br><br>population-weighted % of population living ≤500 m from a park entrance<br><br>Enhanced Vegetation Index	Census block group (2285)	Simple linear regression	Fair
Herrera et al., 2018	Germany: Munich and Dresden (urban)	Highest educational status reported (categorized in 2 levels - elementary education and secondary/advanced technical/higher education)	NDVI	Individual/household (500m buffer around home) (1632)	Summary statistics (e.g. means/medians/proportions)	Good

		Occupational status reported (categorized in six levels - employee, university student, vocational trainee, unemployed, self-employed and other)<br><br>Job type reported (classified following International Standard classification of Occupations ISCO-88, categorized in 5 job groups: clerks, professionals and technicians, healthcare professionals, plant machine operators and elementary occupations)				
Hill et al., 2012	USA: Danville (Rural)	Tertiles of median family income by block group	% of block group devoted to parks<br><br>Mean number of physical activity outlets by block group income<br><br>Walkability index	Block group (39)	Univariate spatial autocorrelation analysis<br><br>MANOVA	Fair
Hillsdon et al., 2007	UK: Entire UK (urban & rural)	Index of Multiple Deprivation 2004	Mean number of exercise facilities in Super Output Area<br><br>Mean number of public/private exercise facilities in Super Output Area<br><br>Mean number of public/private pools in Super Output Area	Super Output Area (32482)	ANOVA	Good
Hirsch et al., 2013	USA: Baltimore, Chicago, Forsyth County, Los Angeles, New York, St. Paul (urban)	Total combined family income<br><br>3 categories of education level<br><br>Dichotomisation of working at least parttime or not	Walk Score	Individual/household (4552)	ANOVA<br><br>Chi-square test	Good
Hirsch et al., 2016	USA: Los Angeles, Chicago, Baltimore, St. Paul, Hinds County, Forsyth County, New York (urban)	Median household income	Number of physical activity destinations in 2000<br><br>Change in number of physical activity destinations between 2000 and 2010	Census tract (8383)	Independent t-test	Fair

Hirsch et al., 2017	USA: Birmingham, Chicago, Minneapolis, Oakland (urban)	Median household income<br><br>% below the poverty line<br><br>% of labour force unemployed	Dichotomisation of high increase and low increase or decreases in m bicycle lane length<br><br>Mean bicycle lane length at baseline<br><br>Change in bicycle lane length over time<br><br>Dichotomisation of high increase and low increase or decreases in % total street length which is served by one or more transit bus routes<br><br>Mean off-road trail length at baseline<br><br>Change % total street length which is served by one or more transit bus routes over time<br><br>Dichotomisation of high increase and low increase or decreases in m off-road trails<br>Mean off-road trail length at baseline<br><br>Change in off-road trail length over time	Neighbourhood (387)	Independent t-test<br><br>Multilevel linear regression	Fair
Hobbs et al., 2017	England: Yorkshire (urban)	Index of Multiple Deprivation	Park access: included as a count if a park boundary edge intersected with participant LSOA (3 categories in descriptive table - 0 = no access, 1 = access to 1 park, 2 = access to 2 or more parks, 2 categories for logistic regression - 0 = no access, 1 = access to 1 or more parks)	Individual/household (4723)	Simple logistic regression	Good
Hoekman et al., 2016	The Netherlands: Entire Netherlands (urban & rural)	Area level deprivation	Mean number of sports facilities per 10,000 inhabitants<br><br>Mean distance in m to nearest sports facility	Postal code areas	ANOVA	Fair
Hoffimann et al., 2017	Portugal: Porto (urban)	The European Deprivation Index	Dichotomisation of availability (or not) of green spaces within an 800 m road distance from the centroids of each census tract<br><br>Number of green spaces within an 800 m road distance from centroid of census tract<br><br>Mean distance (in hectometre) to the green spaces within 800 m from centroid of census tract	Census tract (2064)	Simple logistic regression	Fair

			Area (squared meters) of green space per inhabitant within an 800 m road distance from centroid of census tract			
Houde et al., 2018	Canada: Montreal (urban)	% low-income population	Distance to the nearest section of the cycling network<br><br>Distance to the nearest cyclist-only path<br><br>Dichotomisation of having access to the cycling network within 500m<br><br>Dichotomisation of access to an exclusive bike path within 500 m	Census tract	Simple linear regression<br><br>Simple logistic regression	Fair
Hughey et al., 2016	USA: Southeastern USA (urban)	Neighbourhood socioeconomic disadvantage index	Number of parks within or intersecting the boundary of the block group	Census block group (255)	Simple negative binomial regression	Fair
Iraegui et al., 2020	Spain: Barcelona (urban)	Neighbourhood average family income	Residential green space accessibility. Dichotomisation of high/low access to green space within 150m, measured as % residents that have access to a green space of at least 0.1 ha (service area)<br><br>Neighbourhood green space accessibility. Dichotomisation of high/low access to green space within 400m, measured as % residents that have access to a green space of at least 1 ha (service area)<br><br>Quarter green space accessibility. Dichotomisation of high/low access to green space within 800m, measured as % residents that have access to a green space of at least 5 ha (service area)<br><br>District green space accessibility. Dichotomisation of high/low access to green space within 1,600m, measured as % residents that have access to a green space of at least 30 ha (service area)	Neighbourhood (73)	Mann-Whitney U	Fair

			City green space accessibility. Dichotomisation of high/low access to green space within 3,200m, measured as % residents that have access to a green space of at least >60 ha (service area)			
Iyer et al., 2020	USA: Pennsylvania (urban & rural)	Census block group median income<br><br>Census block group % poverty<br><br>Census block group % adults age 25 and older with less than high school<br><br>Census block group median home value	NDVI	Individual/household (128568)	Summary statistics (e.g. means/medians/proportions)	Good
Jamalishahni et al., 2023	Australia: Brisbane (urban)	Index of Relative Disadvantage	% of green space within 400m/800m/1600m of participant's home by neighbourhood disadvantage	Individual/household (400, 800, and 1,600m buffer around home) (3778)	Chi-square test	Good
James et al., 2016	USA: California, Connecticut, Florida, Maryland, Massachusetts, Michigan, New Jersey, New York, Ohio, Pennsylvania, Texas (urban & rural)	Census tract median income<br><br>3 categories of husband's highest education<br><br>Census tract median home value	NDVI	Individual/household (250m buffer around home) (108603)	Summary statistics (e.g. means/medians/proportions)	Good
Jenerette et al., 2007	USA: Phoenix (urban)	Median household income	Soil-Adjusted Vegetation Index	Census tract (634)	Bivariate correlation	Fair
Jepson et al., 2022	Canada: Montreal (urban)	Low-income population after tax (%)	Distance to closest park (meters)<br><br>Hectares of park per 1000 inhabitants (E2SFCA)	Dissemination areas and census blocks (6301)	Simple linear regression	Fair
Jimenez et al., 2020	USA: Massachusetts (urban)	Dichotomisation of household income % >US$70,000 per year<br><br>Census tract median household income<br><br>Mother's education % >=college graduate	NDVI	Individual/household (90m buffer around home) (460)	Summary statistics (e.g. means/medians/proportions)	Good

		Father's education % ≥college graduate				
Jimenez et al., 2022	USA: Whole country (urban & rural)	Census tract median income ($)<br><br>Parental education (mother and father, low education = less than high school)<br><br>Husband's education (university studies)<br><br>Parental occupation (unskilled labourer)<br><br>Census tract median home value ($)	NDVI	Individual/household (270m buffer around home) (13594)	Summary statistics (e.g. means/medians/proportions)	Good
Jones et al., 2015	USA: Los Angeles, Chicago, Baltimore and Baltimore County, St. Paul, Forsyth County, New York City (urban)	Median household income	Dichotomisation of ≥1 park intersecting the census tract<br><br>Number of parks intersecting the census tract<br><br>Census tract park density (1 mile buffer)<br><br>Dichotomisation of ≥1 commercial recreational facility intersecting the census tract<br><br>Number of commercial recreational facilities intersecting the census tract<br><br>Census tract commercial recreational facility density (1 mile buffer)<br><br>Census tract density of parks and recreational facilities (1 mile buffer)	Census tract (7139)	Simple logistic regression<br><br>Simple negative binomial regression<br><br>Simple linear regression	Fair
Kamel et al., 2014	USA: Texas (urban)	Census tract income (categorized in 3 even tertiles - low, medium and high)	Park availability (number of parks that intersected each census tract)	Census tract (112)	Kruskall-Wallis	Good
Kenyon et al., 2019	Scotland: Glasgow and Edinburgh (urban & rural)	Scottish Index of Multiple Deprivation	Destination accessibility (based on National Destinations Accessibility Index from New Zealand, per output area)<br><br>Residential density (How compact residences are across land areas, per hectare)	1000 m network buffer around the output-area centroid(30066)	Bivariate correlation	Fair

			Intersection density (Directness and availability of alternative routes from one point to another, per km2)<br><br>Walkability index (constructed from the weighted sum of destination accessibility, residential density and intersection density)			
Khomenko et al., 2020	Austria: Vienna (urban)	SEP-index	Percentage of green space surface area per sub-district	Subdistrict (250)	Bivariate correlation	Fair
Ki et al., 2021	South Korea: Seoul (urban)	Dichotomisation of upper and lower classes median income by household size	Green View Index	Individual/household (500m buffer around home) (2350)	Mann-Whitney	Fair
Ki et al., 2023	USA: Los Angeles (urban)	Median income<br><br>% of population without a college degree<br><br>Ratio of workers without a vehicle	Walk Score	Block group (2430)	Spatial lag regression	Good
Kiani et al., 2023	Canada: Montreal (urban)	Pampalon material deprivation score	NDVI	Census tract (689)	Summary statistics (e.g. means/medians/proportions)<br><br>Poisson regression with Gaussian spatial random effects following a conditional autoregressive correlation model	Good
Kim et al., 2020	USA: Austin, Texas (urban)	Census block income (categorized as low and high income)	Percentage of park surface	Census block (457)	Independent t-test	Fair

			Percentage of water features (from satellite images)<br><br>NDVI: cloud-free image from 4 August 2009, with a resolution of 30x30m. Operationalized as the mean NDVI of each census block group.			
Kim et al., 2022	South Korea: Seoul (urban)	% of basic livelihood security recipients	Mean 1-mile kernel density of urban green space per Dong district	Dong district (424)	Simple linear regression<br><br>Geographically weighted regression	Good
Kim et al., 2023a	USA: Birmingham, Chicago, Minneapolis, Oakland (urban)	Neighbourhood deprivation score	Dichotomisation of having a park within 5 km of residence<br><br>NDVI	Individual/household (5,000m buffer around home) (924)	Chi-square test<br><br>Independent t-test	Fair
Kim et al., 2023b	USA: Austin (urban)	Dichotomisation of low/high income neighbourhoods by Austin median	% park in census tract<br><br>% water features<br><br>NDVI	Census tract (210)	Independent t-test	Fair
Kim et al., 2023c	USA: Phoenix (urban)	Per capita income on block group level<br><br>% people without a high school diploma<br><br>% housing without a vehicle	Distance to closest park in m<br><br>Number of local parks<br><br>Total acreage of local parks within walking distance	Census block group (2095)	Spatial autoregressive regression	Good
Knight et al., 2018	USA: Buffalo (urban)	Poverty level, %: Persons total family income compared to the poverty threshold appropriate for that persons family size and composition<br><br>Unemployment, %	Walk score	Census section (284)	Summary statistics (e.g. means/medians/proportions)	Fair
Koohsari et al., 2011	Australia: Melbourne (urban)	SEIFA-IRSAD	Minimum distance from centroid of parcel to centroid of full-access public open space in m2	Census collection district	ANOVA	Poor
Koohsari et al., 2020	Japan: Entire Japan (urban & rural)	Japanese national index of neighbourhood deprivation (municipality-level SES)	Neighbourhood walkability index	Municipality (1880)	Summary statistics (e.g. means/media	Fair

					ns/proportions)	
Koschinsky et al., 2017	USA: Washington D.C. (urban)	Dichotomisation of high/very low census tract median household income	Walk Score	Neighbourhood (115)	Independent t-test	Fair
Kruize et al., 2007a	The Netherlands: The Rijnmond region (urban)	Household income	Availability of m2 public green space per resident within 500 m<br><br><75 m2/>75 m2 of public green space per resident within 500 m	Individual/household (500m from home) (445559)	Kruskall-Wallis	Fair
Kruize et al., 2007b	The Netherlands: Region within a radius of 25 km around Amsterdam (urban)	Household income	Availability of m2 public green space per resident within 500 m	Individual/household (500m from home) (1075037)	Kruskall-Wallis	Poor
Lafary et al., 2008	USA: Evansville (urban & rural)	Median household income<br><br>% residents in poverty<br><br>Owner occupied housing value	NDVI	Block groups (159)	Geographically weighted regression	Fair
Lakes et al., 2014	Germany: Berlin (urban)	Indicator of social development (index)	NDVI	Planning units (434)	Bivariate correlation	Fair
Lamb et al., 2010	Scotland: Glasgow, Edinburgh, Dundee, Aberdeen (urban & rural)	Quintiles of % of individuals who are income deprived	Mean number of all physical activity facilities per 1,000 residents<br><br>Mean number of public physical activity facilities per 1,000 residents<br><br>Mean number of private physical activity facilities per 1,000 residents<br><br>Mean number of all sports facilities per 1,000 residents	Datazone (6505)	Multilevel negative binomial regression	Fair
Lane et al., 2022	USA: Alabama (urban & rural)	% of population below poverty level<br><br>% of population without bachelor's degree	% of each tract with physical activity deserts	Census tract (1179)	Simple linear regression<br><br>Geographically weighted regression	Fair

Lang et al., 2022	USA: USA wide (urban & rural)	4 categories of annual household income<br><br>4 categories of educational level<br><br>Neighbourhood socioeconomic status index	Walk Score	Individual/household (12846)	Chi-square test	Good
Langford et al., 2012	UK: Cardiff (urban)	The Townsend Deprivation Index	Network distance to nearest bus stop<br><br>Total number of bus stops within a 400 m threshold network distance<br><br>Number of unique bus stops within the threshold network distance – this excludes bus stops that simply repeat access to the same service at a greater network distance<br><br>Number of unique bus routes within the threshold network distance<br><br>Total number of weekday busses within 400m network distance<br><br>Ratio of bus to population with floating catchment area without decay<br><br>Ratio of bus to population with floating catchment area with linear decay<br><br>Ratio of bus to population with floating catchment area with Butterworth (Gaussian) decay	Output area (992)	Bivariate correlation<br><br>Summary statistics (e.g. means/medians/proportions)	Fair
Lee et al., 2007	Japan: Metropolitan Tokyo and a city from northeastern Japan (urban & rural)	Dichotomisation of currently employed yes/no	Dichotomisation of high/low walkable region	Individual/household (432)	Chi-square test	Fair
Lee et al., 2016	USA: Southeastern Metropolitan Area (urban)	Median Household Income (USD)<br><br>Unemployment rate<br><br>Median home value<br><br>% Owner occupied houses	Walk Score	Individual/household (500)	Bivariate correlation	Good

		Social vulnerability index (vulnerability to natural and man-made disasters because of population and housing characteristics (age, low income, disability, home value)				
Lee et al., 2022	South Korea: Seongman and Daegu (urban)	Low/high neighbourhood SEP dichotomisation based on district average monthly income and poverty rate	Access to parks (based on 8 items that included transit stops, separate pathways, separate bike lanes, safe crossing aids, main land uses, traffic volume, and slope)<br><br>Neighbourhood park area within urban core areas (km2)	District (4)	Mann-Whitney test<br><br>Summary statistics (e.g. means/medians/proportions)	Fair
Lee et al., 2023	Singapore: Singapore (urban)	4 categories of years of education	NDVI	Individual/household (1,000m buffer around home) (268)	Simple linear regression	Fair
Leung et al., 2010	USA: California	Neighbourhood Deprivation Index	Sum of street shoulders, curb extensions, traffic circles, street signs asking drivers to watch out for children, and playground equipment within a quarter-mile radius of the girl's residence	Individual/household (215)	Bivariate correlation	Good
Li et al., 2009a	USA: Portland (urban)	Dichotomisation of annual household income on resident level<br><br>Neighbourhood median family income<br><br>Dichotomisation of ≥12 years of education	Walkability score consisting of land-use mix, street connectivity, public transit stations, and green and open spaces	Individual/household (1145) and census block groups (120)	Summary statistics (e.g. means/medians/proportions)	Fair
Li et al., 2009b	USA: Portland (urban)	Neighbourhood median household income	Walkability score consisting of land-use mix, street connectivity, public transit stations, and green and open spaces	Census block groups (120)	Independent t-test	Fair
Li et al., 2015	USA: Hartford, Connecticut (urban)	Census block per-capita income (USD)<br><br>Low/high education (Proportion of people without/with high school degree)<br><br>Owner-occupied units (houses)	Green view index	Census block group (87)	Bivariate correlation<br><br>Simple linear regression	Good

Li et al., 2016	USA: Hartford, Connecticut (urban)	Census block per-capita income (USD)<br><br>Low/high education (Proportion of people without high school degree/ Proportion of people with bachelor's or higher degrees)<br><br>Owner-occupied units (houses)	Proximity to urban parks (proportion of residential parcels in the 400m buffer zone around each park ~service area of the park)	Census block group (90)	Bivariate correlation<br><br>Simple linear regression	Fair
Liu et al., 2021	USA: Chicago: Illinois (urban)	Census-tract income categorized as low-income (under $43,600 = Illinois threshold in 2018) and non-low-income (over $43.600)	Urban green space accessibility (area of UGS in census tract)	Census tract (801)	Simple linear regression<br><br>Palma ratio	Good
Lohmus et al., 2021	Sweden: Stockholm County (urban & rural)	Educational level (categorized in 3 levels - primary, secondary and higher education)<br><br>Annual individual income (categorized in 3 levels - <225 tSEK, 225-450 tSEK and > 450 tSEK)	NDVI	Individual/household (50m buffer around home) (2060)	Chi-square test	Good
Majekodunmi et al., 2020	Schotland: Glasgow (urban)	Scottish Index of Multiple Deprivation	% Landcover public park and garden in area (areas of enclosed land, designed, constructed managed and maintained as a public park or garden)<br><br>% Landcover amenity green spaces in area<br><br>% Landcover natural/semi-natural green space in area (areas that are undeveloped or previously developed with natural or introduced habitats or colonized by vegetation and wildlife including woodland and wetland areas)<br><br>% Landcover other functional green space in area (other types of open space as required by the local authority)<br><br>% Landcover green corridor in area (routes such as canals, river corridors and old railway lines linking different areas within a city or surrounding country sides or parks and mostly used for walking, cycling or horse riding)	Hectare (17645)	Summary statistics (e.g. means/medians/proportions)	Fair

			% Landcover sports areas (large flat areas of grassland or synthetic surface used generally for various sporting activities)<br><br>% Landcover play space for children and teenagers (safe and accessible areas for children to play)			
Malecki et al., 2014	USA: Wisconsin (urban & rural)	Family income in 4 categories: <100% of federal poverty level (FPL), 100-199% FPL, 200-399% FPL, 400%+ FPL<br><br>Education status in three categories: high school or less, some college, college or beyond<br><br>Economic hardship index	Prevalence of parks<br><br>Prevalence of walking and biking trails<br><br>Sidewalk density (total sidewalk length per total segment length within a 400m buffer)	Individual/household (400m buffer around home) (890)and census block (939)	Summary statistics (e.g. means/medians/proportions)	Good
Marek et al., 2021	New Zealand: Whole country (excluding oceanic islands) (urban & rural)	Area-level deprivation (NZdep2018)	Access to green spaces - median proximity from any place in the meshblock to each green space (Euclidean distance) based on a 50x50m grid for each meshblock<br><br>Access to blue spaces - median proximity from any place in the meshblock to each blue space (Euclidean distance) based on a 50x50m grid for each meshblock<br><br>Access to physical activity facilities - distance from population-weighted centroid of the 2018 meshblock calculated via road network	Meshblock administrative units (52923)	Chi-square test	Good
Martinuzzi et al., 2018	USA: San Juan (Puerto Rico) (urban)	Census block group average home value (USD)<br><br>Census block group Median Household Income (USD)	Non-residential NDVI<br><br>Residential NDVI	Census block groups (789)	Simple linear regression	Fair
Martori et al., 2020	Spain: Barcelona (urban)	Household income (continuous)	Playground accessibility as minimum street network distance to the nearest playground (between census tract centroid and playground)<br><br>Population group accessibility to playground (enhanced two-step floating catchment areas)	Census section (1061)	Simple linear regression<br><br>Generalized Methods of Moments (GS2SLS) - Lag model	Poor

			Playground accessibility as minimum street network distance to the nearest playground (between census tract centroid and playground)<br><br>Population group accessibility to playground (enhanced two-step floating catchment areas)			
Mavoa et al., 2015	Australia: Melbourne (urban)	IRSAD	Road network distance from SA1 population-weighted centroid to natural and semi-natural open space<br><br>Size in m2 of the nearest natural or semi-natural open space<br><br>Road network distance from SA1 population-weighted centroid to protected green area<br><br>Size in m2 of the nearest organised protected area<br><br>Road network distance from SA1 population-weighted centroid to parkland or garden<br><br>Size in m2 of the nearest parkland and garden<br><br>Road network distance from SA1 population-weighted centroid to organised recreation area<br><br>Size in m2 of the nearest organised recreation area	Statistical Area level 1 (8910)	ANOVA	Good
McCarthy et al., 2017	USA: County in southeastern USA (urban & rural)	Dichotomisation of eligibility to receive free/reduced lunch at school	Dichotomisation of having a park with a playground within 0,5 mile of home	Individual/household (0.5 mile/800m buffer around home) (13469)	Multilevel logistic regression	Fair
McMorris et al., 2015	Canada: Entire urban Canada	5 categories of yearly household income level	NDVI	Individual/household (500m buffer around home) (69910)	ANOVA	Fair
Mears et al., 2019	UK: Sheffield (urban)	Carstairs Deprivation Index	Accessibility to any green space<br><br>Provision of any green space	Individual/household (25264)	Summary statistics (e.g. means/media	Fair

			Population pressure of any green space<br><br>Accessibility to 'good' green space (based on PPG17 assessment)<br><br>Provision of 'good' green space (based on PPG17 assessment)<br><br>Population pressure of 'good' green space (based on PPG17 assessment)<br><br>Accessibility to greenspace with provision for children and young people<br><br>Provision of greenspace with provision for children and young people<br><br>Population pressure of greenspace with provision for children and young people		ns/proportion s)	
Miller et al., 2019	USA: Tallahassee (urban)	3 categories of block-group income level	Number of parks per income group<br><br>Sum of park acreage per income group<br><br>Average park acreage per income group	Block group (38)	Jonckheere-Terpstra test	Poor
Moccia et al., 2023	Italy: Turin (urban & rural)	Equivalised Household Income Indicator	building density, facility richness, connectivity density, Shannon index (a quantitative measure of land-use diversity), walkability, blue spaces, green spaces, population density, number of facilities related to unhealthy food, NDVI, length of public bus lines, and number of bus stops within 300m buffer of residential address	Individual/household (300m buffer around home)(1989)	Simple linear regression	Good
Molaodi et al., 2012	UK: UK wide (urban & rural)	Quintiles of LSOA income deprivation	Mean number of indoor physical activity facilities per 10,000 population	Lower Super Output Area (32482)	Two-level Poisson regression	Poor
Moore et al., 2008	USA: Baltimore city and county, Forsyth County, Manhattan and the Bronx (urban)	Tertiles of median household income	Dichotomisation of not having a park in a tract<br><br>Dichotomisation of not having a recreational facility in a tract	Census tract (685)	Simple logistic regression	Fair

Mora et al., 2021	Chile: Santiago de Chile (urban)	Socio-Material Territorial Index	% households that has a bike lane within 300 m of their home	Household	Summary statistics (e.g. means/medians/proportions)	Poor
Motoc et al., 2023	The Netherlands: Amsterdam, Oss, and Zwolle (urban & rural)	Neighbourhood socioeconomic position index<br><br>Mean neighbourhood income<br><br>neighbourhood-level % low-income earners<br><br>Number of persons receiving social security beneficiaries per 1,000 people in neighbourhood<br><br>Neighbourhood-level mean house price	% green space per 4-digit postal code area	Individual/household (1512)	Bivariate correlation	Good
Mouratidis et al., 2020	Norway: Oslo (urban)	Neighbourhood deprivation index	Mean % of green space within a 500 m radius from the centroid of neighbourhood<br><br>Aggregate number of departures per hour in the peak period from all public transit stops within a radius of 500 m from the centroid of neighbourhood	Neighbourhood (34)	Bivariate correlation<br><br>Simple linear regression	Fair
Mustafa et al., 2023	USA: New York City (urban)	% of block below poverty line<br><br>Median household income<br><br>Unemployment rate	% of inhabitants that have geographical access to a green space<br><br>Average area of green space per capita<br><br>Average area of green space per zip code	Block group and postal code area	Bivariate correlation	Poor
Nayha et al., 2013	Finland: Oulu, Lapland, and Helsinki metropolitan area (urban & rural)	4 categories of educational level	Population density (inhabitants/km2) in each participant's resident 1 km2 grid	Individual/household (5363)	Summary statistics (e.g. means/medians/proportions)	Good
Neckerman et al., 2009	USA: New York City (urban)	Dichotomisation of poor (≥20% poverty rate) and non-poor census tracts	% of census tracts with bicycle lanes or greenways<br><br>% of census tracts with subway stop within 0.8 km from centre of the tract	Census tract (2172)	Chi-square test	Fair

			Density of bus stops per km2		Independent t-test	
Nesbitt et al., 2016	USA: Portland-Vancouver metro area (urban)	% of block group with bachelor's degree<br><br>% of block group with master's degree<br><br>Annual income per capita	NDVI	Block group	Simple linear regression	Poor
Nesbitt et al., 2019	USA: Chicago, Houston, Indianapolis, Jacksonville, Los Angeles, New York, Phoenix, Portland, Seattle, and St. Louis (urban)	Mean per capita income in the past 12 months	Sum of area in m2 of all parks within 1,000 m of block group centroids<br><br>NDVI	Block group (5196, 2135, 701, 499, 7574, 12985, 2012, 830, 1999, 1134, 5195, 2135, 701, 499, 7574, 12985, 2012, 830, 1999, and 1134) and census tracts (1773, 802, 263, 189, 2680, 2536, 763, 292, 599, 362, 1773, 802, 263, 189, 2680, 2536, 763, 292, 599, and 362)	Bivariate correlation	Fair
Nieuwenhuijsen et al., 2018	Spain: Barcelona (urban)	Census tract deprivation status - SES (MEDEA)	Percentage green space within the census tract using land cover maps (Urban Atlas) and 300m buffer<br><br>Percentage blue space within the census tract using land cover maps (Urban Atlas) and 300m buffer<br><br>Census tract NDVI (Normalized Difference of Vegetation Index): average NDVI from the images	Individual/household (792649)	Bivariate correlation	Good

			within the greenest season (April to July) collected during the 5 year follow-up, from Landsat-8 (30m resolution)			
Odijk et al., 2023	The Netherlands: Rotterdam - The Hague metropolitan region (urban)	Average income level per km2 (for each postal code) - stratified as low, below average, average, above average and high	Job accessibility by bike (extent to which jobs can be reached by bicycle, depending on the number of jobs accessible and travel time	Postal code	Simple linear regression	Fair
Olsen et al., 2022	Scotland: Entire Scotland (urban & rural)	Quintiles of datazone income deprivation, measured with Scottish Index of Multiple Deprivation	Number of public open spaces within a 10-minute walk of residential location<br><br>% of residential locations with access to a public open space within 10-min walk<br><br>Residential dwellings with access to public open space within 10-min walk per 100,000 dwellings<br><br>Number of recreational, sports pitches, and facilities within a 10-minute walk of residential location<br><br>% of residential locations with access to a recreational, sports pitch, or facility within 10-min walk<br><br>Residential dwellings with access to recreational, sports pitch, or facility within 10-min walk per 100,000 dwellings<br><br>Number of public transport stops within a 10-minute walk of residential location<br><br>Number of high frequency public transport stops (≥5 transit movements at a stop per hour between the hours of 6am and 9pm) within a 10-minute walk of residential location<br>% of residential locations with access to a public transport stop within 10-min walk<br><br>% of residential locations with access to a high-frequency public transport stop within 10-min walk	Postal code (146190)	Summary statistics (e.g. means/medians/proportions)	Fair

			Residential dwellings with access to public transport stop within 10-min walk per 100,000 dwellings<br><br>Residential dwellings with access to high frequency public transport stop within 10-min walk per 100,000 dwellings			
Olsen et al., 2023	Scotland: Entirecountry (urban & rural)	Scottish Index of Multiple Deprivation	Natural space coverage (green space)<br><br>Open space (Number of bodies of water, landscape features, recreational features)<br><br>Municipal parks (Public parks within 800m of home)<br><br>Retail outlets (food and non-food)<br><br>Residential address count (residential density)<br><br>The presence of a gymnasium, sports hall, leisure centre and a range of specific sports facilities and pitches were used as indicators of recreational, sports pitches and facilities<br><br>Number of public transport stops within 800m of home	Individual/household (800m buffer around home) (687)	Summary statistics (e.g. means/medians/proportions)	Fair
O'Regan et al., 2021	Ireland: Dublin, Cork, and Galway (urban)	Household median gross income<br><br>% of residents aged 15 years and over with at least an ordinary bachelor's degree<br><br>Unemployment rate	NDVI<br><br>Green View Index	Small Area (3335)	Summary statistics (e.g. means/medians/proportions)	Fair
Padilla et al., 2016	France: Nice metropolitan area (urban & rural)	Deprivation index	% of the geographic area occupied by green spaces within the total area of a census block	Census block (236)	Bivariate correlation	Fair
Panter et al., 2008	UK: Norwich (urban)	Annual household income<br><br>Index of Multiple Deprivation	Road network distance from participant's homes to nearest sports facility and/or gym	Individual/household (401)	Bivariate linear model<br><br>Summary statistics (e.g.	Fair

					means/medians/proportions	
Park et al., 2020	USA: Metro Columbus and Metro Atlanta (urban)	Economic indicator (index)	Number of parks & trails within 1,800 m of census block centroid<br><br>Number of golf courses and sports fields within 1,800 m of census block centroid<br><br>NDVI	Block groups (1348 and 2591)	Simple linear regression<br><br>Geographically weighted regression	Fair
Parker et al., 2021	USA: Davenport, Bettendorf, Rock Island, and Moline (urban)	Median household income in the past 12 months<br><br>% college-educated for those of 25 years and older<br><br>% white-collar jobs<br><br>% employed of those 16 years and older	Dichotomisation of whether the centroid of a census block group is within a 400-meter (m) distance of the QCA bikeway network	Census block groups (241 and 246)	Summary statistics (e.g. means/medians/proportions)	Fair
Pascual et al., 2020	USA: Rhode Island (urban & rural)	Census tract average household income	Average household income of census tracts within two miles of a community park compared to the state average household income	Census tract	Independent t-test	Fair
Pearce et al., 2010	UK: Entire UK (urban & rural)	Quintiles of income deprivation domain from the IMD	Mean % of small area land surface classified as greenspace	Census Area Statistics Wards (10654)	Bivariate correlation	Fair
Pearsall et al., 2012	USA: Philadelphia (urban)	Census tract median household income ($)	NDVI	Census tract (370)	Simple linear regression	Fair
Pereira et al., 2023	Portugal: Lisbon Metropolitan Area (urban)	SES index (using PCA)	Percentage of area occupied by urban green areas<br><br>Total of green area (ha)<br><br>Density (dwelling density)<br><br>Diversity (variety) of activities: number of different activities, based on points of interest, classified into six types: retail; entertainment; civic and institutional; food-related; office; and recreation (0-6)	Census tract (25885)	Bivariate spatial autocorrelation	Fair

			Design was measured as the density of road intersections (nodes) with four or more links.<br><br>Slope measured as the percentage of the census block buffer area with a slope below 8% (Por)<br><br>Public transport provision was calculated as an index that considered the level of service within walking distance of each census block (including bus, train, tram, and ferryboat)			
Pham et al., 2011	Canada: Montreal (urban)	% low income people in DA	Modified Soil Adjusted Vegetation Index	Dissemination Areas (1773)	Bivariate correlation	Fair
Pinault et al., 2021	Canada: Entire Canada (urban)	Quintiles of household income<br><br>Dichotomisation of low/high income<br><br>4 categories of educational level for those 25 years or older<br><br>Dichotomisation of employment status<br><br>Economic dependency of individual's Dissemination Area	NDVI	Individual/household (500m buffer around home) (5306800)	Summary statistics (e.g. means/medians/proportions)	Good
Plans et al., 2019	Spain: Madrid (urban)	Census section SEP index	Quartiles of % green land cover within 500m of the population-weighted centroid of the participants' residence census section	Individual/household (500m buffer around participant's census section centroid)(1625)	Summary statistics (e.g. means/medians/proportions)	Good
Pratt et al., 2023	USA: Franklin county	Census tract poverty rate of participant's home address<br><br>Census tract unemployment rate of participant's home address	Area in m2 of all total open spaces within 5-minute/10-minute walk of participant's address<br><br>Number of fitness and/or recreation facilities within 5-minute/10-minute walk of participant's address	Individual/household (0.25 mile/400m and 0.5 mile/800m buffer around home)(772)	Bivariate correlation	Good

Pun et al., 2018	USA: Entire USA (urban & rural)	Median household income<br><br>Family income<br><br>3 categories of education level	NDVI	Individual/household (1,000m buffer around home) (4118)	Summary statistics (e.g. means/medians/proportions)<br><br>Chi-square test	Good
Quinton et al., 2022	Canada: Victoria, Burnaby, Coquitlam, Richmond, Surrey, Vancouver, Calgary, Edmonton, Regina, Saskatoon, Winnipeg, Sudbury, Windsor, London, Brampton, Markham, Mississauga, Oakville, Richmond Hill, Toronto, Vaughan, Ottawa, Gatineau, Laval, Longuel, Montreal, Terrebonne, Quebec, Sherbrooke, Halifax , and St. John's (urban)	Median household income<br><br>% of population 25 years or older with bachelor's degree	Greenness fraction (index)	Dissemination Areas	Spatial lag regression	Fair
Rachele et al., 2017	Australia: Brisbane (urban)	Index of Relative Socioeconomic Disadvantage	Street connectivity (400 m pedshed ratio around population weighted centroid of SA1), divided in quintiles<br><br>Number of stops within SA1	Statistical Areas Level 1 (2460)	Simple logistic regression	Fair
Ranchod et al., 2014	USA: Baltimore, Maryland; Chicago, Illinois; Forsyth County, North Carolina; Los Angeles, California; New York, New York; St. Paul, Minnesota (urban & rural)	Household income, categorized in 4 levels (<$20,000, $20,000–49,999, $50,000–74,999, or >$75,000)<br><br>Education level (less than high school, high school, some college, or college graduate/graduate school)<br><br>Neighbourhood unemployment rate (mean of the years of follow up)	Density of recreational facilities around participant's home address (1 miles buffer)	Individual/household (1mle/1,600m buffer around home)(6168)	Summary statistics (e.g. means/medians/proportions)	Good

		Neighbourhood poverty rate (mean of the years of follow up)<br><br>Neighbourhood household income (mean of the years of follow up)				
Regidor et al., 2008	Spain: Entire Spain (urban & rural)	3 categories of number of times gross domestic product per capita was below the 40th percentile in 1980, 1990, and 2000 for each province	Mean number of sports facilities per 1,000 population per province	Individual/household (17917)	Summary statistics (e.g. means/medians/proportions)	Fair
Richardson et al., 2010	New Zealand: Whole country (urban)	Area-level deprivation (NZdep2001) - reflects 8 dimensions of material and social deprivation (income, employment, communication, support, qualifications, owned home, living space, dry living conditions)	Total green space cover (% cover)<br><br>Usable green space cover (% cover of urban parkland/open space, beaches, non-commercial forestry)	Census area units (1009)	Simple linear regression	Fair
Rigolon et al., 2014	USA: Denver (Colorado) (urban)	3 levels of median household income	% of Census blocks with access to a park within a 1 mile street-network buffer	Census blocks (1481)	Kruskall-Wallis	Poor
Rigolon et al., 2017	USA: Denver (Colorado) (urban)	Census block median household income, divided by tertiles: ow-income ($7750-$44,762), medium-income ($44,763-$67,444), and high-income ($67,445-$237,785)	Park proximity (CBGs within and beyond 1/4 mile from any park) - 2 categories: access (at least 1 park in the area) and no access (no park in the area)<br><br>Park acreage (Acres of parks within 1/4 mile of a CBG per thousand-population)<br><br>Park acreage per young population (acres of parks within 1/4 mile of a CBG per thousand population under 18)<br><br>Park proximity (CBGs distance to any park; difference in income, ethnicity, and age between CBGs within and beyond 1/4 mile from any park)	Census block group (473)	Jonckheere-Terpstra test<br><br>Mood's Median Test	Fair
Rigolon et al., 2018	USA: 99 cities with the largest population in the USA (urban)	Median household income	% park surface of the city area<br><br>% of residents living within 800m of a park<br><br>% park surface of the city area<br><br>% of residents living within 800m of a park	City/town (99)	Bivariate correlation<br><br>Simple linear regression	Fair

Rivera et al., 2023	USA: Santa Clara county (California) (urban)	Modified Darden-Kamel CSI	NDVI	Census tract (372)	Summary statistics (e.g. means/medians/proportions)	Fair
Robinson et al., 2018	Norway: Oslo; Lithuania: Kaunas; UK: Bradford; France: Nancy and Poiters; Spain: Gipuzkoa, Sabadell, Valencia, Greece: Heraklion (urban) (urban)	Dichotomisation of high or mid/low education level<br><br>"grunnkrets" area tertiles of average personal income<br><br>% of population with tertiary (university level) education of the voting district<br><br>Index of Multiple Deprivation<br><br>French European Deprivation Index<br><br>Urban Vulnerability index<br><br>% of population with tertiary (university level) education of the aggregated lower census area<br><br>Dichotomisation of high or mid/low occupational status<br><br>Dichotomisation of high or mid/low family income	Distance from the home to nearest blue or green space with an area greater than 5,000 m2<br><br>Difference in park access between lowest and highest income group<br><br>Number of bus stops within 300 m of participant's address<br><br>NDVI<br><br>Walkability index including population density, street connectivity, facility richness index, and land use SE	Individual/household (300m buffer araound home) (10559, 3625, 10008, 669, 574, 594, 575, 695, and 746)	Simple linear regression	Goodf
Robinson et al., 2022	UK: 68 urban centres (urban)	Index of Multiple Deprivation	PCA greenness measure (combination of NDVI, tree cover, and OS publicly accessible urban greenspace)	Urban centre boundary (68)	Bivariate correlation	Good
Rodgers et al., 2012	UK: Swansea City (urban)	Welsh Index of Multiple Deprivation	Dichotomisation of access to a park or playground within 100, 200, 300, 400, 500, 600, 700, 800, 900, and 1000 m of residence	Individual/household (500m buffer around home) (103450)	Simple logistic regression	Fair
Rodriguez-Loureiro et al., 2022	Belgium: Antwerp, Ghent, Brussels, Charleroi, and Liège (urban)	Neighbourhood socioeconomic position index	NDVI	Individual/household (500m buffer	Summary statistics (e.g. means/media	Good

				around home) (2309236)	ns/proportion s)	
Rundle et al., 2007	USA: New York City (urban)	Census tract poverty rate	Land use mix<br><br>Population density (inhabitants/km2)<br><br>Intersection density<br><br>Number of bus stops per km2<br><br>Number of subway stops per km2	Census tract (1989)	Bivariate correlation	Fair
Saelens et al., 2003	USA: San Diego (California) (urban)	Dichotomisation of completed college/university (yes/no)	Walkability composite measure	Individual/household (107)	Chi-square test	Fair
Sallis et al., 2018	USA: Baltimore (Maryland)-Washington DC, and Seattle-King County (Washington) metropolitan areas (urban)	Median Household Income (USD) - operationalized as lower and higher income (median split)	Walkability index - calculated using GIS (Geographic Information Systems; King County data from 2006 and Maryland data from 2003) measures of net residential density, street connectivity, retail floor area ratio, and land use mix)	Census block groups (447)	Summary statistics (e.g. means/medians/proportions)	Fair
Saporito et al., 2015	USA: USA cities with more than 25.000 people, Meadow Woods, and Franklin Town (urban)	7 categories of income % above poverty threshold	NDVI	Census block group	Summary statistics (e.g. means/medians/proportions)<br><br>Simple linear regression	Fair
Schaeffer et al., 2019	France: Grenoble-Alpes Metropole (urban)	Proportion of poor (low income) households in a rectangular 200x200m cell - Poor (low income) households are defined as those whose income per consumption unit (before taxes and benefits) is <60% of the median of Metropolitan France distribution (i.e. 11249.40€)	NDVI	Individual/household (184485)	Monte Carlo permutation test	Fair
Scheurer et al., 2017	Australia: Melbourne, Sydney, Adelaide, and Brisbane (urban)	SEIFA-IRSAD	% of the population of each SEIFA quintile that are located either within an 800-metre radius of a rail	Statistical Area 1	Summary statistics (e.g. means/media	Fair

			station or ferry terminal, or within a 400-metre linear corridor around a tram or bus route		ns/proportion s)	
Schinasi et al., 2023	USA: Philadephia (urban)	Proportion of population living in poverty in the census tract - operationalized in three levels, tertiles (low= 0 to 0.16, moderate= >0.16 to 0.32, and high= >0.32 to 0.74)	Census tract average NDVI	Census tract (376)	Summary statistics (e.g. means/media ns/proportion s)	Good
Schneider et al., 2015	Germany: Cologne (urban)	% of population under the age of 65 receiving social welfare<br><br>% of population with children receiving social welfare<br><br>% of single parents receiving social welfare<br><br>Unemployment rate<br><br>Youth unemployment rate (<25 years old)	Total number of physical activity facilities per 1,000 inhabitants<br><br>Total number of physical activity facilities suitable for children per 1,000 children<br><br>Total number of physical activity facilities suitable for adults per 1,000 adults	Social areas (18)	Bivariate correlation	Good
Schneider et al., 2019	Germany: Mannheim (urban)	Employment rate (% employees among working-age population)<br><br>Unemployment quotient (% = number of unemployed people among all 15-64-year olds)<br><br>Long-term unemployed among all those unemployed (%)<br><br>All inhabitants who are receiving state support payments (%)	Availability of playgrounds (number of playground per sqkm)<br><br>Accessibility to playgrounds (playground distance)<br><br>Playground area per child (sqm per child)	Social areas (44)	Two-level Poisson regression	Fair
Schule et al., 2017	Germany: Munich (urban)	SEP index (unemployment, foreigners and people with migration history, population density, education and occupation	Green space cover within the neighbourhood - no buffer, 200m buffer, 400m buffer, 600m buffer, 800m buffer, 1000m buffer, 1500m buffer, 2000m buffer, 2500m buffer, and 3000m buffer<br><br>Green space cover from neighbourhood centroid - 1000m radius, 1500m radius, 2000m radius, 2500m radius, 3000m radius	Sub districts (108)	Log-gamma regression	Good

Sharifi et al., 2021	Australia: Melbourne (urban)	SA2 Low Income Proportion (proportion of households in the lowest and second equivalised disposable household income quintiles - lowest 40%)	All green spaces access index: incorporates factors of the area, network distance, and extent of congestion (population density) in determining access of different regions to green space.	Statistical Areas Level 2 (306)	Mann-Whitney test<br><br>Moran's I Spatial autocorrelation	Fair
Shih et al., 2022	Taiwan: Taipei city (urban)	Household salary (Annual average household salary in 2018 in NT$)<br><br>Education attainment (percentage of people with university degree and above)	Green space proportion: Ratio of greenspace area to a given neighbourhood<br><br>Distance to greenspaces: Average distance to nearest greenspace based on land cover (Mode value) - meters<br><br>Accessibility to recreational parks and greenspaces: Average distance to nearest recreational greenspaces (Mode value) - meters<br><br>Size of water bodies: Surface area of water bodies in a given neighbourhood (m2)<br><br>Distance to waters: Average distance to nearest water bodies (Mode value) - meters<br><br>NDVI	Neighbourhood (991)	Bivariate correlation	Good
Shin et al., 2023	South Korea: Seoul (urban)	Low-SES neighbourhood: a dummy variable indicating whether a neighbourhood's SES is low (= 1) or not (=0). Low-SES neighbourhoods must meet two criteria: (a) the percentage of neighbourhood residents with a higher education level (bachelor's degree or higher) is less than 80% of the study area's median proportion of highly-educated residents; and (b) the neighbourhood poverty rate should be higher than the median poverty rate of Seoul	Protected bicycle lane density (km/km2)<br><br>Unprotected exclusive bicycle lane density (km/km2)<br><br>Bicycle-priority lane density (km/km2)<br><br>Shared bicycle–pedestrian path density (km/km2)<br><br>Sidewalk density (km/km2)	Haengjeongdong (smallest administrative unit within Seoul) (422)	Independent t-test	Fair
Slater et al., 2022	USA: Alabama, Florida, Georgia, Kentucky, Louisiana,	Census block group median household income categorized into 3: low (0-45.000), middle (45.000-70.000), high (>70.000)	Presence of sidewalks<br><br>Presence of crosswalks	Street segment (4363)	Simple logistic regression	Fair

	Maryland, Missouri, North Carolina, South Carolina, Tennessee, Texas (urban & rural)		Presence of bike lanes<br><br>Presence of bike parking			
Spotswood et al., 2021	USA: 17 states (urban)	Quartiles of median household income	The total acres of park within 1,000m of the centroid of census blocks<br><br>NDVI	Census block group (142325)	Summary statistics (e.g. means/medians/proportions)	Fair
Stucki et al., 2023	Sweden: Uppsala and Västmanland	Area-based income (quartiles with cutoffs at baseline of 140,138, 150,842, and 161,591 SEK)	NDVI	Individual/household (500m buffer around home) (20244)	Poisson regression based on GEE	Good
Suárez et al., 2020	Norway: Oslo Metropolitan Area (urban)	Average household income for each census tract	Areas for daily recreation reachable in 30 min walking. Categorized in 3 distance buffers: less than 10 min walking (833m), between 10-30 min walking (833m-2.5km) and more than 30 min walking (over 2.5km)	Census tract	Bivariate correlation	Fair
Subiza-Perez et al., 2023	Spain: Asturias, Gipuzkoa, Sabadell, and Valencia (urban & rural)	Area SES (MEDEA2011)<br><br>Maternal education (primary - ref, secondary, university)	Green, blue, public open spaces and vegetation index	Individual/household (100, 300, and 500m buffer around home) (1738)	Simple logistic regression<br><br>bivariate logistic model	Good
Sugiyama et al., 2015	Australia: Metropolitan Adelaide, South Australia (urban)	Index of Relative Socioeconomic Disadvantage	Residential density (number/km2): The density of dwellings in residential area within the 1 km buffer area<br><br>Intersection density (number/km2): The density of intersections (3-way or more) within the 1 km buffer area<br><br>Land use mix: Entropy value based on the size of residential, retail, and recreational land uses<br><br>Net retail area ratio (%): The proportion of total retail floor area to total retail parcel area within the 1 km buffer area	Individual/household (1,000m buffer around home) (1500)	Independent t-test	Good

Suminski et al., 2011	USA: Large Midwestern metropolitan area (urban)	Dichotomisation of median household income	Mean number of public parks per neighbourhood	Neighbourhood (16)	Independent t-test	Fair
Sun et al., 2021	USA: Los Angeles County (urban & rural)	% living below two times the federal poverty level<br><br>% housing burdened low-income households<br><br>% of population over 25 with less than a high school education<br><br>% of the population over the age of 16 that is unemployed and eligible for the labour force<br><br>Population score	The sum of % of all types of green space in each 350-degree panoramic street view image per census tract<br><br>NDVI	Census tract (2343)	Bivariate correlation<br><br>Generalised linear mixed models with spherical spatial covariance structure	Good
Svastisalee et al., 2012	Denmark: Copenhagen (urban)	Dichotomisation of high/low % of 16- to 85-year-olds in each neighbourhood lacking a high school diploma<br><br>4 categories of income level<br><br>SEIFA-IRSAD	Ratio urban green space to rode area<br><br>Rode being in top 25% of total length of cycling and walking paths<br><br>Total length of walking/cycling paths<br><br>Rode being in top 25% of intersection density<br><br>Total intersection density<br><br>Dichotomisation of having at least 1 sports centre in the neighbourhood	Rodes (389)	Simple logistic regression<br><br>Simple linear regression	Fair
Tan et al., 2017	Singapore: Main island of Singapore (urban)	Average annual household income per housing type, calculated for region, planning zone, and subzone by: weighting of proportion of population in different housing types within region/planning zone/subzone.	Park area per person	Region (323), planning area (55), and subzone (5)	Simple linear regression	Fair
Tayyebi et al., 2016	USA: Metropolitan Los Angeles (South California) (urban)	Median annual household income from the United State Census Bureau ($)	NDVI	Census block	Structural Equation Modelling	Fair

Thornton et al., 2016	USA: San Diego (urban)	Block group median household income	Sidewalks presence and width	Block group (111, 782, 405, 332, and 138)	Multilevel linear regression	Fair
Timperio et al., 2007	Australia: Melbourne (urban)	SEIFA-IRSAD	Number of public open spaces with free access per 1,000 people<br><br>Area of free access public open space in km2 per person<br><br>Number of public open spaces with 'restricted access' imposed entry fees, regulated hours of entry or restricted access to a subset of the population per 1,000 people<br><br>Area of public open spaces with 'restricted access' imposed entry fees, regulated hours of entry or restricted access to a subset of the population in km2 per person<br><br>Number of sport/recreation open spaces per 1,000 people<br><br>Area of sports/recreation open spaces per person in km2	Postal code (177)	ANOVA	Good
Tiznado-Aitken et al., 2022	Chile: Santiago (urban)	Indice Socio Material Territorial (ISMT) - Territorial Socio Material Index	Bike lanes coverage (500m buffers)	Individual/household (6075760)	Summary statistics (e.g. means/medians/proportions)	Poor
Vallee et al., 2020	Canada: Montreal Metropolitan Area (urban)	Maternal educational level, categorized in 4 levels (very low, low, intermediate, high)	Proportion of respondents with at least one large park (more than 20.000 m2) intersecting with the half-mile circular or road network buffer from residential address<br><br>Proportion of population with at least one bike lane intersecting with the half-mile circular buffer from residential address<br><br>Proportion of respondents with at least one recreational sport centres (physical fitness	Individual/household (0.5 mile/800m buffer around home) (1101)	Summary statistics (e.g. means/medians/proportions)<br>Bivariate logistic model	Fair

			facilities, membership sports and recreation clubs, bowling centres) available in the half-mile circular or road network buffer from residential address			
vanDiepen et al., 2023	The Netherlands: Northern Netherlands (i.e.: Groningen, Friesland, Drenthe) (urban & rural)	NSES: composite score based on 1) percentage of persons aged 15 to 65 years not receiving social assistance benefits, 2) aver-age household income, 3) average value of a house in the neighbourhood, and 4) percentage owner-occupied houses. These indicators reflect the general financial (1 and 2), occupational (2), and housing (3 and 4) status of a neighbourhood	Availability of pay-for-use physical activity facilities as the number of each of these outlets within a straight-line distance of 1 km from the participants' residential address. Divided into having no, one or at least one facility within 1 km, to reflect availability vs no availability.	Individual/household (1,000m buffer around home) (146629)	Summary statistics (e.g. means/medians/proportions)	Good
VanVelzen et al., 2023	The Netherlands: Entire Netherlands (urban & rural)	% low-income households in school neighbourhood<br><br>% lower-educated people in school neighbourhood<br><br>% residents in neighbourhood aged between 15 and 75 years participating in the labor force	Sum of the size of the available grassland (in m2), the length of hedges and bushes (in m), and the number of trees across land use type within 50 m buffer of school	School (5773)	Simple linear regression<br><br>Spatial lag regression	Good
Vaughan et al., 2013	USA: Kansas City (urban & rural)	Census tract median household income	Mean number of parks whose boundaries intersected the boundary of each census tract<br><br>Mean amount of park space (acres) of all parks that intersect the census tract	Census tract (170)	MANCOVA	Fair
Venter et al., 2023	Norway: Oslo (urban)	Sub-district mean income over 2008 to 2019<br><br>Income trend from 2008 to 2019	Euclidean distance between residential areas and the closest water body (lake, river or fjord) >1 hectare<br><br>NDVI	Sub-district (delbydel) (99)	Simple linear regression	Fair
Villanueva et al., 2016	Spain: Madrid (urban)	Quintiles of neighbourhood unemployment rate<br><br>Quintiles of neighbourhood mean habitable home surface area	The number of sports facilities per 1,000 population per neighbourhood	Individual/household (727)	Chi-square test	Fair
Wang et al., 2019	USA: Miami-Dade county (urban)	Census block median household income	NDVI	Postal code (249405)	ANOVA	Fair

Wang et al., 2022	Taiwan: Taichung City (urban)	Census block group median household income<br><br>Years of education	Spatial clustering in number of parks per census block group<br><br>Spatial clustering in total park space (cumulative area of all the parks throughout the census block groups)	Census block group (108)	ANOVA	Fair
Wen et al., 2013	USA: Entire USA (urban & rural)	Census tract % residents living under federal poverty level	Population-weighted distance to the closest seven parks<br><br>% vegetated land within a census tract	Census tract (11079, 17067, 12654, 15857, 3648, and 11458)	Multilevel linear regression	Fair
Wende et al., 2022	USA: Northeast , Midwest, South, and West USA (urban & rural)	Three categories of county-level median household income	Physical Activity Environment Index	County (217, 1055 1422, 448, 1166, 1335, and 641)	ANOVA	Fair
Willis et al., 2023	USA and Canada: Entire USA and Canada (urban & rural)	4 categories of household income<br><br>Highest level of education completed or currently undertaking<br><br>4 categories of census tract % with less than high school diploma	NDVI	Individual/household (50m buffer around home) (8563)	Summary statistics (e.g. means/medians/proportions)	Good
Witten et al., 2011	New Zealand: North Shore, Waitakere, Wellington, and Christchurch (urban)	New Zealand Deprivation Index	Neighbourhood destination index	Meshblock (1577, 1338, 1807, 2880)	Summary statistics (e.g. means/medians/proportions)	Fair
Wolch et al., 2005	USA: Los Angeles (urban)	4 categories of census tract median household income<br><br>4 categories of census tract poverty rate	% residents living within one-quarter mile of a park edge<br><br>Park acres per 1,000 population<br><br>Park acres per 1,000 population within one-quarter mile buffer of park edge<br><br>Park acres per 1,000 population under 18 years within one-quarter mile buffer of park edge	Census tract (720)	Summary statistics (e.g. means/medians/proportions)	Fair

Wüstemann et al., 2017	Germany: 53 major cities (urban)	5 categories of monthly household income<br><br>5 categories of education level	Amount of urban green space in 500m-buffer around the household in square meter<br><br>Euclidian Distance between the household and the nearest urban green space in m	Individual/household (500m buffer around home) (4404)	Simple linear regression	Good
Yang et al., 2019	Canada: Edmonton (urban)	Neighbourhood median income<br><br>% of residents without high school diploma in neighbourhood<br><br>% renters in neighbourhood	Counts of outdoor recreational facilities within the 800-m coverage of each school	School (281)	Poisson regression	Fair
Yasumoto et al., 2021	Japan: Osaka (urban)	Quintiles of proportions professional and managerial workers in census area	Total number of parks within 800m road network distance from centroid of census area<br><br>Total park area in ha within 800m road network distance from centroid of census area<br><br>Park area per capita in m2 within 800m road network distance from centroid of census area	Small census areas 'Chocho-aza'	Bivariate correlation	Fair
Yeager et al., 2018	USA: Louisville (urban)	Block group median household income<br><br>Area deprivation index	NDVI	Individual/household (250m buffer around home) (408)	ANOVA	Good
Yeager et al., 2023	USA: Louisville, Kentucky (urban)	Current household income (US$) - categorized in 3 levels (<$20.000, 20,000-$65,0000, >$65.000)<br><br>Education attainment - categorized in 3 levels (High School Diploma or less, Some college, 4-year degree or higher)<br><br>Maternal education attainment - categorized in 3 levels (High School Diploma or less, Some college, 4-year degree or higher)<br><br>Employment status - 2 categories (employed and not employed)	NDVI	Individual/household (100m buffer around home) (636)	ANOVA<br><br>Chi-square test<br><br>Simple linear regression	Good

Younan et al., 2016	USA: Los Angeles county and surrounding areas (urban)	Household socioeconomic status index	NDVI	Individual/household (1,000m buffer around home) (1287)	ANOVA	Good
Yu et al., 2014	USA: Austin (urban)	Census tract poverty rate	Residential density<br><br>Road density (total miles of streets per total area in acres)<br><br>Sidewalk completeness (total miles of sidewalks per total miles of streets x2)<br><br>Bike lane completeness (total miles of bike lanes per total miles of street x2)<br><br>Street intersection density (number of street intersections per total area in acres)<br><br>Land use mix<br><br>Transit stop density (number of transit stops per total area in acres)	Census tract (162)	Simple linear regression	Fair
Zandieh et al., 2017	UK: Birmingham (urban)	Index of Multiple Deprivation	% of green space<br><br>Street connectivity: number of junctions in a home-based neighbourhood/the area (hectare) of the home-based neighbourhood	Neighbourhood (173)	Independent t-test	Fair
Zandieh et al., 2019	UK: Birmingham (urban)	Index of Multiple Deprivation	Pedestrian network distance in m from participant's home to closest and to largest gate of each green space within 2 km radius around participant's home	Individual/household (173)	Independent t-test	Fair
Zhang et al., 2021	Hong Kong (urban)	Socioeconomic deprivation index	Number of parks per tertiary planning unit	Tertiary planning units (209)	Simple negative binomial regression	Good
Zhang et al., 2022	USA: Fayetteville, Winston-Salem, Charlotte, Raleigh (urban)	Social Vulnerability Index<br><br>% of students eligible for free and reduced lunch	Amount of green infrastructure (a merge of trees, shrubs, grass/lawn and other herbaceous vegetation) per neighbourhood and per schoolyard	Census tract and school (42, 36, 91, and 97)	Bivariate correlation	Good

					Simple linear regression<br><br>Spatial lag regression	
Zhang et al., 2023a	USA: Hartford (urban)	Census block group poverty rate<br><br>Census block group median household income<br><br>% of population 25 years old and over who are not high school graduates<br><br>% of population 25+ with bachelor's degree or higher<br><br>Block group median house value for the block group<br><br>% of owner-occupied housing units	% of population with access to parks within a half mile	Census block group	Simple linear regression<br><br>Spatial lag regression	Fair
Zhang et al., 2023b	Hong Kong (urban)	Dichotomisation of low income and not low income<br><br>Dichotomisation of higher education and under college<br><br>3 categories of occupation type	Park density (point density of parks within the neighbourhood buffers around the respondents' residences)<br><br>NDVI	Individual/household (1977)	Mann-Whitney	Good
Zhang et al., 2024	New Zealand: Auckland (urban)	New Zealand Index of Deprivation	Green space accessibility taking into account distance decay	Individual/household (3813)	Bivariate correlation	Good
Zhou et al., 2023	Ireland: Belfast (urban)	% of population living in households whose income is below 60% of the Northern Ireland median income.<br><br>% of population with no or low qualification<br><br>% of population at working age that are employment deprived	Greenness coverage (ratio of cells with NDVI values >0.25 of the total number of cells)<br><br>Distance to nearest greenspace in m<br><br>Distance to nearest water features in m	Community	Bivariate correlation<br><br>Spatial autoregressive regression<br><br>Spatial error model	Fair

Zhu et al., 2008	USA: Austin (urban)	Dichotomisation of school poverty rate (defined by % of students eligible for free or reduced-price lunch)	Sidewalk completeness (total miles of sidewalks per total miles of streets x2)<br><br>Street density (total footage of streets per total acres of the area<br><br>Street intersection density (number of street intersections per total area in acres)<br><br>Population density (total population per total acres of the area)<br><br>Land use mix	School's attendance area (73)	ANOVA<br><br>Simple linear regression	Fair

SEP: Socioeconomic position; nSES: Neighbourhood socioeconomic position; ANCOVA: Analysis of Covariance; NDVI: Normalised Difference Vegetation Index; SEIFA-IRSAD: Socio-Economic Indexes for Areas - Index of Relative Socio-economic Advantage and Disadvantage; SA: Statistical Area; NZDep: New Zealand Index of Deprivation; ANOVA: Analysis of Variance; IRSD: Index of Relative Socio-economic Disadvantage; IMD: Index of Multiple Deprivation. Note: For some studies, the number of units analysed was unclear. Therefore, not for all units an n is reported.
