# Supplementary material for: Socioeconomic inequalities in exposure to neighbourhood environments for physical activity: a systematic review
Source: Int J Behav Nutr Phys Act. 2026 Apr 9;23:58. doi: 10.1186/s12966-026-01912-1 (PMC13231669; doi:10.1186/s12966-026-01912-1)
Supplement: Supplementary file 7 — Supplementary Material 7. [file 12966_2026_1912_MOESM7_ESM.pdf]

Questions + notes

1. Introduction: Were the aims/objectives of the study clear?
2. Methods: Was the sample frame taken from an appropriate population or ecological (e.g. census tracts) base so that it closely represented the target/reference population or ecological context under investigation?
3. Methods: Was SEP measured with more than 1 single indicator?
4. Methods: Were SEP-variables adequately described or do the authors refer to other paper(s) where the variables are adequately described? Note: Indicate 'partly' if more SEP-indicators were measured, of which some are adequately described/referred to description, and some were not
5. Methods: Were environmental variables adequately described or do the authors refer to other paper(s) where the variables are adequately described? Note: Indicate 'partly' if more environmental indicators were measured, of which some are adequately described/referred to description, and some were not
6. Methods: Were the SEP- and environmental exposures measured within the same year (or one year before/after)? Note: Indicate 'partly' if more SEP-indicators were measured, of which some were measured within the same year or one year before/after, and some were not
7. Methods: Was SEP analysed at individual level? Note: Indicate 'partly' if more SEP-indicators were measured, from which some were measured on individual level and some on a larger level
8. Methods: Was missing data handling adequately described? E.g. Did the authors indicate that missing data was removed, or missing data was imputated, or something else?
9. Methods: Were the methods (including statistical methods) sufficiently described to enable them to be repeated?
10. Results: Were the basic data adequately described? E.g. Is their descriptives table understandable, is basic information on participants described, e.t.c.
11. Results: If appropriate, was information about non-responders described? Note: For ecological studies, this can be answered with 'not relevant'. Papers that use secondary data should also provide information about non-responders. Indicate 'no' if no information on non-response was provided.
12. Discussion: Were the limitations of the study discussed?
13. Other: Were funding sources and conflicts of interest disclosed? Note: Indicate 'partly' if either only the conflicts of interest or the funding source is disclosed.
14. Other: Was ethical approval or consent of participants attained? Note: for ecological studies, this can be answered with 'not relevant'.

<50% of total points: poor quality
50-75% of total points: fair quality
>75% of total points: good quality

**Question**	**Answer**	**Points**	**Relevant q**	**Overall quality**		**Options**
1.	yes	1				yes
2.	yes	1				partly
3.	yes	1				no/notstated
4.	yes	1				not relevant
5.	yes	1				
6.	partly	0.5			Data collection land cover not clear	
7.	no/notstat	0				
8.	no/notstat	0				
9.	yes	1				
10.	yes	1				
11.	not relevan	0				
12.	yes	1				
13. Other:	yes	1				
14. Other:	not relevan	0			publicly available data	
		9.5	12	79.16667		

Question	Answer	Points	Relevant q	Overall quality		Options
1.	yes	1				yes
2.	yes	1				partly
3.	no/notstat	0				no/notstated
4.	yes	1				not relevant
5.	yes	1				
6.	yes	1				
7.	no/notstat	0				
8.	no/notstat	0				
9.	yes	1				
10.	yes	1				
11.	not relevar	0				
12.	yes	1				
13. Other:	partly	0.5			Funding disclosed, for conflict of interest only financial disclosure mentioned	
14. Other:	not relevar	0			Publicly available data	
		8.5	12	70.83333		

Question	Answer	Points	Relevant q	Overall quality	Options
1.	yes	1			yes
2.	yes	1			partly
3.	yes	1			no/notstated
4.	yes	1			not relevant
5.	yes	1			
6.	yes	1			
7.	yes	1			
8.	no/notstat	0			
9.	yes	1			
10.	yes	1			
11.	not relevar	0			
12.	yes	1			
13. Other:	yes	1			
14. Other:	no/notstat	0			
		11	13	84.61538	

Bezold2018

Question	Answer	Points	Relevant q	Overall quality
1.	yes	1		
2.	partly	0.5		
3.	yes	1		
4.	yes	1		
5.	yes	1		
6.	yes	1		
7.	yes	1		
8.	yes	1		
9.	yes	1		
10.	yes	1		
11.	not relevar	0		
12.	yes	1		
13. Other:	yes	1		
14. Other:	no/notstat	0		
		11.5	13	88.46154

Options
yes
partly
no/notstated
not relevant

Question	Answer	Points	Relevant q	Overall quality		Options
1.	yes	1				yes
2.	yes	1				partly
3.	yes	1				no/notstated
4.	partly	0.5				not relevant
5.	yes	1				
6.	yes	1				
7.	no/notstat	0				
8.	yes	1				
9.	yes	1				
10.	yes	1				
11.	not relevar	0				
12.	yes	1				
13. Other:	yes	1				
14. Other:	yes	1				
		11.5	13	88.46154		

Chaparro2018

Question	Answer	Points	Relevant q	Overall quality
1.	yes	1		
2.	yes	1		
3.	yes	1		
4.	yes	1		
5.	partly	0.5		
6.	yes	1		
7.	no/notstat	0		
8.	yes	1		
9.	yes	1		
10.	yes	1		
11.	not relevar	0		
12.	yes	1		
13. Other:	yes	1		
14. Other:	yes	1		
		11.5	13	88.46154

Options

yes
partly
no/notstated
not relevant

Cheruvalath2022

Question	Answer	Points	Relevant q	Overall quality		Options
1.	yes	1				yes
2.	yes	1				partly
3.	yes	1				no/notstated
4.	yes	1				not relevant
5.	yes	1				
6.	yes	1				
7.	yes	1				
8.	yes	1				
9.	yes	1				
10.	yes	1				
11.	not relevar	0				
12.	yes	1				
13. Other:	no/notstat	0				
14. Other:	no/notstat	0				
		11	13	84.61538		

Conderino2021

Question	Answer	Points	Relevant q	Overall quality		Options
1.	yes	1				yes
2.	yes	1				partly
3.	no/notstat	0				no/notstated
4.	yes	1				not relevant
5.	yes	1				
6.	yes	1				
7.	no/notstat	0				
8.	yes	1				
9.	yes	1				
10.	yes	1				
11.	not relevar	0				
12.	yes	1				
13. Other:	no/notstat	0				
14. Other:	no/notstat	0				
		9	13	69.23077		

Creatore2016

Question	Answer	Points	Relevant q	Overall quality	Options
1.	yes	1			yes
2.	yes	1			partly
3.	yes	1			no/notstated
4.	yes	1			not relevant
5.	yes	1			
6.	partly	0.5			
7.	no/notstat	0			
8.	yes	1			
9.	yes	1			
10.	yes	1			
11.	not relevar	0			
12.	yes	1			
13. Other:	yes	1			
14. Other:	yes	1			
		11.5	13	88.46154	

Csomós2021

Question	Answer	Points	Relevant q	Overall quality
1.	yes	1		
2.	yes	1		
3.	yes	1		
4.	yes	1		
5.	yes	1		
6.	yes	1		
7.	no/notstat	0		
8.	no/notstat	0		
9.	yes	1		
10.	yes	1		
11.	not relevar	0		
12.	partly	0.5		
13. Other:	yes	1		
14. Other:	no/notstat	0		
		9.5	13	73.07692

Options

yes
partly
no/notstated
not relevant

Dobbs2023

Question	Answer	Points	Relevant q	Overall quality	Options
1.	yes	1			yes
2.	yes	1			partly
3.	yes	1			no/notstated
4.	no/notstat	0			not relevant
5.	partly	0.5			
6.	no/notstat	0			
7.	no/notstat	0			
8.	no/notstat	0			
9.	partly	0.5			
10.	partly	0.5			
11.	not relevar	0			
12.	partly	0.5			
13. Other:	partly	0.5			
14. Other:	yes	1			
		6.5	13	50	

Farkas2022

Question	Answer	Points	Relevant q	Overall quality		Options
1.	yes	1				yes
2.	yes	1				partly
3.	yes	1				no/notstated
4.	yes	1				not relevant
5.	yes	1				
6.	yes	1				
7.	yes	1				
8.	no/notstat	0				
9.	yes	1				
10.	yes	1				
11.	not relevar	0				
12.	no/notstat	0				
13. Other:	yes	1				
14. Other:	no/notstat	0				
		10	13	76.92308		

Question	Answer	Points	Relevant q	Overall quality	Options
1.	yes	1			yes
2.	yes	1			partly
3.	yes	1			no/notstated
4.	yes	1			not relevant
5.	yes	1			
6.	no/notstat	0			
7.	yes	1			
8.	yes	1			
9.	yes	1			
10.	yes	1			
11.	not relevar	0			
12.	yes	1			
13. Other:	yes	1			
14. Other:	no/notstat	0			
		11	13	84.61538	

Collyer2022

Question	Answer	Points	Relevant q	Overall quality	Options
1.	yes	1			yes
2.	yes	1			partly
3.	yes	1			no/notstated
4.	yes	1			not relevant
5.	yes	1			
6.	yes	1			
7.	no/notstat	0			
8.	yes	1			
9.	yes	1			
10.	yes	1			
11.	not relevar	0			
12.	yes	1			
13. Other:	yes	1			
14. Other:	yes	1			
		12	13	92.30769	

Crawford2008

Question	Answer	Points	Relevant q	Overall quality		Options
1.	partly	0.5				yes
2.	yes	1				partly
3.	yes	1				no/notstated
4.	yes	1				not relevant
5.	no/notstat	0				
6.	yes	1				
7.	no/notstat	0				
8.	no/notstat	0				
9.	no/notstat	0				
10.	partly	0.5				
11.	not relevar	0				
12.	partly	0.5				
13. Other:	no/notstat	0				
14. Other:	no/notstat	0				
		5.5	13	42.30769		

Knight2018

Question	Answer	Points	Relevant q	Overall quality
1.	yes	1		
2.	yes	1		
3.	yes	1		
4.	no/notstat	0		
5.	yes	1		
6.	partly	0.5		
7.	yes	1		
8.	no/notstat	0		
9.	partly	0.5		
10.	yes	1		
11.	not relevar	0		
12.	no/notstat	0		
13. Other:	no/notstat	0		
14. Other:	no/notstat	0		
		7	13	53.84615

Options

yes
partly
no/notstated
not relevant

Koohsari2011

Question	Answer	Points	Relevant q	Overall quality
1.	yes	1		
2.	yes	1		
3.	yes	1		
4.	yes	1		
5.	yes	1		
6.	no/notstat	0		
7.	no/notstat	0		
8.	no/notstat	0		
9.	no/notstat	0		
10.	no/notstat	0		
11.	not relevar	0		
12.	yes	1		
13. Other:	no/notstat	0		
14. Other:	no/notstat	0		
		6	13	46.15385

Options

yes
partly
no/notstated
not relevant

Koohsari2020

Question	Answer	Points	Relevant q	Overall quality
1.	partly	0.5		
2.	yes	1		
3.	yes	1		
4.	yes	1		
5.	partly	0.5		
6.	yes	1		
7.	no/notstat	0		
8.	no/notstat	0		
9.	partly	0.5		
10.	yes	1		
11.	not relevar	0		
12.	yes	1		
13. Other:	yes	1		
14. Other:	no/notstat	0		
		8.5	13	65.38462

Options

yes
partly
no/notstated
not relevant

Mears2019

Question	Answer	Points	Relevant q	Overall quality		Options
1.	yes	1				yes
2.	yes	1				partly
3.	yes	1				no/notstated
4.	partly	0.5				not relevant
5.	yes	1				
6.	no/notstat	0				
7.	yes	1				
8.	no/notstat	0				
9.	yes	1				
10.	partly	0.5				
11.	no/notstat	0				
12.	yes	1				
13. Other:	yes	1				
14. Other:	no/notstat	0				
		9	14	64.28571		

Majekodunmi2020

Question	Answer	Points	Relevant q	Overall quality
1.	partly	0.5		
2.	partly	0.5		
3.	yes	1		
4.	yes	1		
5.	partly	0.5		
6.	no/notstat	0		
7.	no/notstat	0		
8.	no/notstat	0		
9.	partly	0.5		
10.	partly	0.5		
11.	not relevan	0		
12.	yes	1		
13. Other:	yes	1		
14. Other:	no/notstat	0		
		6.5	13	50

Options

yes
partly
no/notstated
not relevant

Malecki2014

Question	Answer	Points	Relevant q	Overall quality
1.	yes	1		
2.	yes	1		
3.	yes	1		
4.	yes	1		
5.	yes	1		
6.	yes	1		
7.	partly	0.5		
8.	yes	1		
9.	yes	1		
10.	yes	1		
11.	not relevar	0		
12.	yes	1		
13. Other:	yes	1		
14. Other:	no/notstat	0		
		11.5	13	88.46154

Options

yes
partly
no/notstated
not relevant

Slater2022

Question	Answer	Points	Relevant q	Overall quality	Options
1.	yes	1			yes
2.	yes	1			partly
3.	no/notstat	0			no/notstated
4.	yes	1			not relevant
5.	yes	1			
6.	no/notstat	0			
7.	no/notstat	0			
8.	yes	1			
9.	yes	1			
10.	yes	1			
11.	not relevar	0			
12.	yes	1			
13. Other:	yes	1			
14. Other:	no/notstat	0			
		9	13	69.23077	

Question	Answer	Points	Relevant q	Overall quality		Options
1.	yes	1				yes
2.	yes	1				partly
3.	no/notstat	0				no/notstated
4.	yes	1				not relevant
5.	yes	1				
6.	yes	1				
7.	no/notstat	0				
8.	no/notstat	0				
9.	yes	1				
10.	partly	0.5				
11.	not relevar	0				
12.	partly	0.5				
13. Other:	yes	1				
14. Other:	no/notstat	0				
		8	13	61.53846		

Zandieh2017

Question	Answer	Points	Relevant q	Overall quality
1.	yes	1		
2.	yes	1		
3.	yes	1		
4.	yes	1		
5.	yes	1		
6.	no/notstat	0		
7.	partly	0.5		
8.	yes	1		
9.	partly	0.5		
10.	yes	1		
11.	not relevar	0		
12.	yes	1		
13. Other:	no/notstat	0		
14. Other:	no/notstat	0		
		9	13	69.23077

Options

yes
partly
no/notstated
not relevant

Question	Answer	Points	Relevant q	Overall quality	Options	Comments
1.	yes	1			yes	
2.	yes	1			partly	
3.	yes	1			no/notstated	
4.	yes	1			not relevant	
5.	yes	1				
6.	yes	1				
7.	no/notstated	0				
8.	not relevant	0				
9.	partly	0.5				
10.	yes	1				
11.	not relevant					
12.	yes	1				
13. Other:	partly	0.5				funding-no, conflict-yes
14. Other:	not relevant	0				
		9	11	81.81818		

Question	Answer	Points	Relevant q	Overall quality	Options	Comments
1.	yes	1			yes	
2.	yes	1			partly	
3.	yes	1			no/notstated	
4.	yes	1			not relevant	
5.	yes	1				
6.	no/notstated	0				
7.	yes	1				
8.	yes	1				
9.	partly	0.5				
10.	yes	1				
11.	yes					
12.	yes	1				
13. Other:	yes	1				
14. Other:	yes	1				
		11.5	14	82.14286		

Question	Answer	Points	Relevant q	Overall quality	Options	Comments
1.	yes	1			yes	
2.	yes	1			partly	
3.	no/notstated	0			no/notstated	
4.	yes	1			not relevant	
5.	yes	1				
6.	yes	1				
7.	no/notstated	0				
8.	yes	1				
9.	partly	0.5				
10.	yes	1				
11.	yes					
12.	partly	0.5				
13. Other:	no/notstated	0				
14. Other:	not relevant	0				
		8	13	61.53846		

Question	Answer	Points	Relevant q	Overall quality	Options	Comments
1.	yes	1			yes	
2.	yes	1			partly	
3.	yes	1			no/notstated	however, they found high collinearity with education, so they dropped the education variable in the
4.	yes	1			not relevant	
5.	yes	1				
6.	no/notstated	0				
7.	no/notstated	0				
8.	no/notstated	0				
9.	partly	0.5				
10.	yes	1				
11.	not relevant					
12.	yes	1				
13. Other:	partly	0.5				
14. Other:	not relevant	0				
		8	12	66.66667		

Question	Answer	Points	Relevant q	Overall quality	Options	Comments
1.	yes	1			yes	
2.	yes	1			partly	
3.	partly	0.5			no/notstated	
4.	yes	1			not relevant	
5.	yes	1				
6.	yes	1				
7.	no/notstated	0				
8.	yes	1				
9.	yes	1				
10.	yes	1				
11.	not relevant					
12.	yes	1				
13. Other:	yes	1				
14. Other:	not relevant	0				
		10.5	12	87.5		

Question	Answer	Points	Relevant q	Overall quality	Options	Comments
1.	yes	1			yes	
2.	partly	0.5			partly	selection of range of SEP levels
3.	yes	1			no/notstated	
4.	yes	1			not relevant	
5.	yes	1				
6.	no/notstat	0				data collection year not mentioned
7.	no/notstat	0				suburb level
8.	no/notstat	0				
9.	yes	1				
10.	yes	1				
11.	not relevant					
12.	yes	1				
13. Other:	partly	0.5				Funding not mentioned
14. Other:	not relevar	0				publicly available data
		8	12	66.66667		

Dahmann2010

Question	Answer	Points	Relevant q	Overall quality	Options
1.	yes	1			yes
2.	yes	1			partly
3.	no/notstated	0			no/notstated
4.	partly	0.5			not relevant
5.	yes	1			
6.	no/notstated	0			
7.	no/notstated	0			
8.	no/notstated	0			
9.	partly	0.5			
10.	yes	1			
11.	not relevant				
12.	no/notstated	0			
13. Other:	no/notstated	0			
14. Other:	not relevant	0			
		5	12	41.66667	

Cohen-Cline2015

Question	Answer	Points	Relevant q	Overall quality		Options
1.	yes	1				yes
2.	yes	1				partly
3.	yes	1				no/notstated
4.	yes	1				not relevant
5.	partly	0.5				
6.	no/notstat	0				
7.	yes	1				
8.	no/notstat	0				
9.	partly	0.5				
10.	yes	1				
11.	no/notstat	0				
12.	yes	1				
13. Other:	yes	1				
14. Other:	yes	1				
		10	14	71.42857		

Question	Answer	Points	Relevant q	Overall quality		Options
1. Introduction: Were the	yes	1				yes
2. Methods: Was the sample	no/notstat	0				partly
3. Methods: Was SEP	no/notstat	0				no/notstated
4. Methods: Were SEP-	yes	1				not relevant
5. Methods: Were	yes	1				
6. Methods: Were the SEP-	yes	1				
7. Methods: Was SEP	yes	1				
8. Methods: Was missing data	yes	1				
9. Methods: Were the	yes	1				
10. Results: Were the basic	partly	0.5				
11. Results: If appropriate,	no/notstat	0				
12. Discussion: Were the	yes	1				
13. Other: Were funding	yes	1				
14. Other: Was ethical	yes	1				
		10.5	14	75		

Question	Answer	Points	Relevant q	Overall quality	Options
1. Introduction:	partly	0.5			yes
2. Methods: Was	yes	1			partly
3. Methods: Was	no/notstat	0			no/notstated
4. Methods: Were	yes	1			not relevant
5. Methods: Were	yes	1			
6. Methods: Were	yes	1			
7. Methods: Was	yes	1			
8. Methods: Was	yes	1			
9. Methods: Were	yes	1			
10. Results: Were	no/notstat	0			
11. Results: If	no/notstat	0			
12. Discussion:	yes	1			
13. Other: Were	partly	0.5			
14. Other: Was	yes	1			
		10	14	71.42857	

Question	Answer	Points	Relevant q	Overall quality	Options
1. Introduction: Were the	yes	1			yes
2. Methods: Was the sample	yes	1			partly
3. Methods: Was SEP	no/notstat	0			no/notstated
4. Methods: Were SEP-	yes	1			not relevant
5. Methods: Were	yes	1			
6. Methods: Were the SEP-	no/notstat	0			
7. Methods: Was SEP analysed	no/notstat	0			
8. Methods: Was missing data	not relevar	0			
9. Methods: Were the	yes	1			
10. Results: Were the basic	partly	0.5			
11. Results: If appropriate,	not relevar	0			
12. Discussion: Were the	yes	1			
13. Other: Were funding	yes	1			
14. Other: Was ethical	yes	1			
		8.5	12	70.83333	

Question	Answer	Points	Relevant q	Overall quality		Options
1. Introduction: Were the	yes	1				yes
2. Methods: Was the	yes	1				partly
3. Methods: Was SEP	no/notstat	0				no/notstated
4. Methods: Were SEP-	yes	1				not relevant
5. Methods: Were	partly	0.5				
6. Methods: Were the SEP-	yes	1				
7. Methods: Was SEP	partly	0.5				
8. Methods: Was missing	yes	1				
9. Methods: Were the	yes	1				
10. Results: Were the basic	yes	1				
11. Results: If appropriate,	partly	0.5				
12. Discussion: Were the	yes	1				
13. Other: Were funding	partly	0.5				
14. Other: Was ethical	yes	1				
		11	14	78.57143		

Question	Answer	Points	Relevant q	Overall quality	Options
1. Introduction:	yes	1			yes
2. Methods: Was	yes	1			partly
3. Methods: Was	yes	1			no/notstated
4. Methods: Were	yes	1			not relevant
5. Methods: Were	yes	1			
6. Methods: Were	yes	1			
7. Methods: Was	yes	1			
8. Methods: Was	yes	1			
9. Methods: Were	yes	1			
10. Results: Were	yes	1			
11. Results: If	no/notstat	0			
12. Discussion:	yes	1			
13. Other: Were	yes	1			
14. Other: Was	yes	1			
		13	14	92.85714	

Hobbs2017

Question	Answer	Points	Relevant q	Overall quality		Options
1. Introduction:	yes	1				yes
2. Methods: Was	partly	0.5				partly
3. Methods: Was	yes	1				no/notstated
4. Methods: Were	yes	1				not relevant
5. Methods: Were	yes	1				
6. Methods: Were	yes	1				
7. Methods: Was	partly	0.5				
8. Methods: Was	no/notstat	0				
9. Methods: Were	yes	1				
10. Results: Were	yes	1				
11. Results: If	no/notstat	0				
12. Discussion:	yes	1				
13. Other: Were	yes	1				
14. Other: Was	yes	1				
		11	14	78.57143		

Question	Answer	Points	Relevant q	Overall quality		Options
1. Introduction: Were the	yes	1				yes
2. Methods: Was the sample	yes	1				partly
3. Methods: Was SEP	yes	1				no/notstated
4. Methods: Were SEP-	yes	1				not relevant
5. Methods: Were	yes	1				
6. Methods: Were the SEP-	no/notstat	0				
7. Methods: Was SEP	no/notstat	0				
8. Methods: Was missing	not relevar	0				
9. Methods: Were the	partly	0.5				
10. Results: Were the basic	yes	1				
11. Results: If appropriate,	not relevar	0				
12. Discussion: Were the	yes	1				
13. Other: Were funding	yes	1				
14. Other: Was ethical	not relevar	0				
		8.5	11	77.27273		

Guo2017

Question	Answer	Points	Relevant q	Overall quality	Options
1. Introduction:	yes	1			yes
2. Methods: Was	yes	1			partly
3. Methods: Was	no/notstat	0			no/notstated
4. Methods: Were	yes	1			not relevant
5. Methods: Were	yes	1			
6. Methods: Were	partly	0.5			
7. Methods: Was	not relevar	0			
8. Methods: Was	not relevar	0			
9. Methods: Were	yes	1			
10. Results: Were	yes	1			
11. Results: If	not relevar	0			
12. Discussion:	yes	1			
13. Other: Were	partly	0.5			
14. Other: Was	not relevar	0			
		8	10	80	

Question	Answer	Points	Relevant q	Overall quality	Options
1. Introduction: Were the	partly	0.5			yes
2. Methods: Was the	yes	1			partly
3. Methods: Was SEP	no/notstat	0			no/notstated
4. Methods: Were SEP-	yes	1			not relevant
5. Methods: Were	yes	1			
6. Methods: Were the SEP-	yes	1			
7. Methods: Was SEP	no/notstat	0			
8. Methods: Was missing	not relevar	0			
9. Methods: Were the	yes	1			
10. Results: Were the basic	yes	1			
11. Results: If appropriate,	not relevar	0			
12. Discussion: Were the	no/notstat	0			
13. Other: Were funding	partly	0.5			
14. Other: Was ethical	not relevar	0			
		7	11	63.63636	

Hart2022

Question	Answer	Points	Relevant q	Overall quality		Options
1. Introduction: Were the	yes	1				yes
2. Methods: Was the sample	no/notstat	0				partly
3. Methods: Was SEP	yes	1				no/notstated
4. Methods: Were SEP-	partly	0.5				not relevant
5. Methods: Were	yes	1				
6. Methods: Were the SEP-	yes	1				
7. Methods: Was SEP analysed	yes	1				
8. Methods: Was missing data	partly	0.5				
9. Methods: Were the	partly	0.5				
10. Results: Were the basic	yes	1				
11. Results: If appropriate,	no/notstat	0				
12. Discussion: Were the	yes	1				
13. Other: Were funding	yes	1				
14. Other: Was ethical	yes	1				
		10.5	14	75		

Question	Answer	Points	Relevant q	Overall quality	Options
1. Introduction: Were the	yes	1			yes
2. Methods: Was the	partly	0.5			partly
3. Methods: Was SEP	yes	1			no/notstated
4. Methods: Were SEP-	yes	1			not relevant
5. Methods: Were	yes	1			
6. Methods: Were the SEP-	yes	1			
7. Methods: Was SEP	yes	1			
8. Methods: Was missing	yes	1			
9. Methods: Were the	yes	1			
10. Results: Were the basic	yes	1			
11. Results: If appropriate,	partly	0.5			
12. Discussion: Were the	yes	1			
13. Other: Were funding	yes	1			
14. Other: Was ethical	yes	1			
		13	14	92.85714	

Question	Answer	Points	Relevant q	Overall quality	Options
1. Introduction: Were the	yes	1			yes
2. Methods: Was the	yes	1			partly
3. Methods: Was SEP	no/notstat	0			no/notstated
4. Methods: Were SEP-	yes	1			not relevant
5. Methods: Were	yes	1			
6. Methods: Were the SEP-	yes	1			
7. Methods: Was SEP	no/notstat	0			
8. Methods: Was missing	no/notstat	0			
9. Methods: Were the	yes	1			
10. Results: Were the basic	partly	0.5			
11. Results: If appropriate,	not relevar	0			
12. Discussion: Were the	no/notstat	0			
13. Other: Were funding	yes	1			
14. Other: Was ethical	not relevar	0			
		7.5	12	62.5	

Question	Answer	Points	Relevant q	Overall quality
1. Introduction: Were the	yes	1		
2. Methods: Was the	yes	1		
3. Methods: Was SEP	yes	1		
4. Methods: Were SEP-	yes	1		
5. Methods: Were	yes	1		
6. Methods: Were the SEP-	partly	0.5		
7. Methods: Was SEP	partly	0.5		
8. Methods: Was missing	no/notstat	0		
9. Methods: Were the	partly	0.5		
10. Results: Were the basic	yes	1		
11. Results: If appropriate,	no/notstat	0		
12. Discussion: Were the	yes	1		
13. Other: Were funding	yes	1		
14. Other: Was ethical	no/notstat	0		
		9.5	14	67.85714

Options
yes
partly
no/notstated
not relevant

Question	Answer	Points	Relevant q	Overall quality	Options
1. Introduction: Were	yes	1			yes
2. Methods: Was the	yes	1			partly
3. Methods: Was SEP	no/notstat	0			no/notstated
4. Methods: Were SEP-	yes	1			not relevant
5. Methods: Were	yes	1			
6. Methods: Were the	yes	1			
7. Methods: Was SEP	no/notstat	0			
8. Methods: Was missing	not relevar	0			
9. Methods: Were the	yes	1			
10. Results: Were the	yes	1			
11. Results: If	not relevar	0			
12. Discussion: Were the	yes	1			
13. Other: Were funding	partly	0.5			
14. Other: Was ethical	not relevar	0			
		8.5	11	77.27273	

Question	Answer	Points	Relevant q	Overall quality	Options
1. Introduction: Were the	yes	1			yes
2. Methods: Was the sample	yes	1			partly
3. Methods: Was SEP	yes	1			no/notstated
4. Methods: Were SEP-	partly	0.5			not relevant
5. Methods: Were	yes	1			
6. Methods: Were the SEP-	no/notstat	0			
7. Methods: Was SEP	no/notstat	0			
8. Methods: Was missing	not relevar	0			
9. Methods: Were the	partly	0.5			
10. Results: Were the basic	yes	1			
11. Results: If appropriate,	not relevar	0			
12. Discussion: Were the	partly	0.5			
13. Other: Were funding	yes	1			
14. Other: Was ethical	yes	1			
		8.5	12	70.83333	

Khomenko2020

Question	Answer	Points	Relevant q	Overall quality		Options
1. Introduction: Were the	yes	1				yes
2. Methods: Was the	yes	1				partly
3. Methods: Was SEP	yes	1				no/notstated
4. Methods: Were SEP-	yes	1				not relevant
5. Methods: Were	yes	1				
6. Methods: Were the SEP-	no/notstat	0				
7. Methods: Was SEP	no/notstat	0				
8. Methods: Was missing	no/notstat	0				
9. Methods: Were the	yes	1				
10. Results: Were the basic	partly	0.5				
11. Results: If appropriate,	not relevar	0				
12. Discussion: Were the	yes	1				
13. Other: Were funding	partly	0.5				
14. Other: Was ethical	no/notstat	0				
		8	13	61.53846		

Question	Answer	Points	Relevant q	Overall quality	Options
1. Introduction: Were the	yes	1			yes
2. Methods: Was the	yes	1			partly
3. Methods: Was SEP	yes	1			no/notstated
4. Methods: Were SEP-	partly	0.5			not relevant
5. Methods: Were	partly	0.5			
6. Methods: Were the SEP-	yes	1			
7. Methods: Was SEP	no/notstat	0			
8. Methods: Was missing	partly	0.5			
9. Methods: Were the	no/notstat	0			
10. Results: Were the basic	partly	0.5			
11. Results: If appropriate,	not relevar	0			
12. Discussion: Were the	yes	1			
13. Other: Were funding	yes	1			
14. Other: Was ethical	not relevar	0			
		8	12	66.66667	

Question	Answer	Points	Relevant q	Overall quality	Options
1. Introduction: Were the	yes	1			yes
2. Methods: Was the	no/notstat	0			partly
3. Methods: Was SEP	yes	1			no/notstated
4. Methods: Were SEP-	partly	0.5			not relevant
5. Methods: Were	yes	1			
6. Methods: Were the SEP-	yes	1			
7. Methods: Was SEP	no/notstat	0			
8. Methods: Was missing	not relevan	0			
9. Methods: Were the	yes	1			
10. Results: Were the	yes	1			
11. Results: If appropriate,	not relevan	0			
12. Discussion: Were the	yes	1			
13. Other: Were funding	partly	0.5			
14. Other: Was ethical	not relevan	0			
		8	11	72.72727	

Question	Answer	Points	Relevant q	Overall quality	Options
1. Introduction:	yes	1			yes
2. Methods: Was	yes	1			partly
3. Methods: Was	yes	1			no/notstated
4. Methods: Were	yes	1			not relevant
5. Methods: Were	yes	1			
6. Methods: Were	yes	1			
7. Methods: Was	no/notstat	0			
8. Methods: Was	no/notstat	0			
9. Methods: Were	yes	1			
10. Results: Were	partly	0.5			
11. Results: If	not relevar	0			
12. Discussion:	yes	1			
13. Other: Were	yes	1			
14. Other: Was	not relevar	0			
		9.5	12	79.16667	

Question	Answer	Points	Relevant q	Overall quality	Options
1. Introduction: Were	yes	1			yes
2. Methods: Was the	yes	1			partly
3. Methods: Was SEP	no/notstat	0			no/notstated
4. Methods: Were SEP-	yes	1			not relevant
5. Methods: Were	partly	0.5			
6. Methods: Were the	yes	1			
7. Methods: Was SEP	no/notstat	0			
8. Methods: Was	not relevar	0			
9. Methods: Were the	yes	1			
10. Results: Were the	yes	1			
11. Results: If	not relevar	0			
12. Discussion: Were	yes	1			
13. Other: Were	yes	1			
14. Other: Was ethical	not relevar	0			
		8.5	11	77.27273	

Question	Answer	Points	Relevant q	Overall quality	Options
1. Introduction: Were the	no/notstat	0			yes
2. Methods: Was the	yes	1			partly
3. Methods: Was SEP	yes	1			no/notstated
4. Methods: Were SEP-	yes	1			not relevant
5. Methods: Were	yes	1			
6. Methods: Were the SEP-	yes	1			
7. Methods: Was SEP	no/notstat	0			
8. Methods: Was missing	partly	0.5			
9. Methods: Were the	yes	1			
10. Results: Were the	partly	0.5			
11. Results: If appropriate,	not relevar	0			
12. Discussion: Were the	yes	1			
13. Other: Were funding	no/notstat	0			
14. Other: Was ethical	not relevar	0			
		8	12	66.66667	

Lohmus2021

Question	Answer	Points	Relevant q	Overall quality		Options
1. Introduction:	partly	0.5				yes
2. Methods: Was	yes	1				partly
3. Methods: Was	yes	1				no/notstated
4. Methods: Were	yes	1				not relevant
5. Methods: Were	yes	1				
6. Methods: Were	partly	0.5				
7. Methods: Was	yes	1				
8. Methods: Was	no/notstat	0				
9. Methods: Were	yes	1				
10. Results: Were	yes	1				
11. Results: If	no/notstat	0				
12. Discussion:	yes	1				
13. Other: Were	yes	1				
14. Other: Was	yes	1				
		11	14	78.57143		

Question	Answer	Points	Relevant q	Overall quality	Options
1. Introduction: Were the	yes	1			yes
2. Methods: Was the	yes	1			partly
3. Methods: Was SEP	yes	1			no/notstated
4. Methods: Were SEP-	yes	1			not relevant
5. Methods: Were	yes	1			
6. Methods: Were the SEP-	yes	1			
7. Methods: Was SEP	no/notstat	0			
8. Methods: Was missing	partly	0.5			
9. Methods: Were the	yes	1			
10. Results: Were the	yes	1			
11. Results: If appropriate,	not relevar	0			
12. Discussion: Were the	yes	1			
13. Other: Were funding	no/notstat	0			
14. Other: Was ethical	not relevar	0			
		9.5	12	79.16667	

Question	Answer	Points	Relevant q	Overall quality	Options
1. Introduction: Were the	yes	1			yes
2. Methods: Was the sample	yes	1			partly
3. Methods: Was SEP	yes	1			no/notstated
4. Methods: Were SEP-	yes	1			not relevant
5. Methods: Were	yes	1			
6. Methods: Were the SEP-	yes	1			
7. Methods: Was SEP	no/notstat	0			
8. Methods: Was missing	not relevar	0			
9. Methods: Were the	yes	1			
10. Results: Were the basic	yes	1			
11. Results: If appropriate,	partly	0.5			
12. Discussion: Were the	partly	0.5			
13. Other: Were funding	yes	1			
14. Other: Was ethical	not relevar	0			
		10	12	83.33333	

Martinuzzi2018

Question	Answer	Points	Relevant q	Overall quality	Options
1. Introduction: Were the	yes	1			yes
2. Methods: Was the sample frame	yes	1			partly
3. Methods: Was SEP measured with	yes	1			no/notstated
4. Methods: Were SEP-variables	partly	0.5			not relevant
5. Methods: Were environmental	yes	1			
6. Methods: Were the SEP- and	yes	1			
7. Methods: Was SEP analysed at	no/notstat	0			
8. Methods: Was missing data	no/notstat	0			
9. Methods: Were the methods	yes	1			
10. Results: Were the basic data	partly	0.5			
11. Results: If appropriate, was	not relevan	0			
12. Discussion: Were the limitations of	yes	1			
13. Other: Were funding sources and	partly	0.5			
14. Other: Was ethical approval or	not relevan	0			
		8.5	12	70.83333	

Question	Answer	Points	Relevant q	Overall quality	Options
1. Introduction: Were the	yes	1			yes
2. Methods: Was the sample	yes	1			partly
3. Methods: Was SEP	yes	1			no/notstated
4. Methods: Were SEP-	partly	0.5			not relevant
5. Methods: Were	yes	1			
6. Methods: Were the SEP-	partly	0.5			
7. Methods: Was SEP analysed	partly	0.5			
8. Methods: Was missing data	yes	1			
9. Methods: Were the	yes	1			
10. Results: Were the basic	yes	1			
11. Results: If appropriate,	not relevar	0			
12. Discussion: Were the	yes	1			
13. Other: Were funding	yes	1			
14. Other: Was ethical	yes	1			
		11.5	13	88.46154	

Question	Answer	Points	Relevant q	Overall quality		Options
1. Introduction: Were the	yes	1				yes
2. Methods: Was the	partly	0.5				partly
3. Methods: Was SEP	yes	1				no/notstated
4. Methods: Were SEP-	yes	1				not relevant
5. Methods: Were	yes	1				
6. Methods: Were the SEP-	no/notstat	0				
7. Methods: Was SEP	no/notstat	0				
8. Methods: Was missing	yes	1				
9. Methods: Were the	yes	1				
10. Results: Were the basic	yes	1				
11. Results: If appropriate,	no/notstat	0				
12. Discussion: Were the	yes	1				
13. Other: Were funding	yes	1				
14. Other: Was ethical	yes	1				
		10.5	14	75		

Odijk2023

Question	Answer	Points	Relevant q	Overall quality	Options
1. Introduction: Were the	yes	1			yes
2. Methods: Was the	yes	1			partly
3. Methods: Was SEP	no/notstat	0			no/notstated
4. Methods: Were SEP-	yes	1			not relevant
5. Methods: Were	yes	1			
6. Methods: Were the SEP-	no/notstat	0			
7. Methods: Was SEP	no/notstat	0			
8. Methods: Was missing	not relevar	0			
9. Methods: Were the	yes	1			
10. Results: Were the basic	partly	0.5			
11. Results: If appropriate,	not relevar	0			
12. Discussion: Were the	yes	1			
13. Other: Were funding	partly	0.5			
14. Other: Was ethical	not relevar	0			
		7	11	63.63636	

Question	Answer	Points	Relevant q	Overall quality	Options
1. Introduction: Were	yes	1			yes
2. Methods: Was the	yes	1			partly
3. Methods: Was SEP	yes	1			no/notstated
4. Methods: Were SEP-	yes	1			not relevant
5. Methods: Were	yes	1			
6. Methods: Were the	partly	0.5			
7. Methods: Was SEP	no/notstat	0			
8. Methods: Was	no/notstat	0			
9. Methods: Were the	yes	1			
10. Results: Were the	yes	1			
11. Results: If	no/notstat	0			
12. Discussion: Were	yes	1			
13. Other: Were funding	yes	1			
14. Other: Was ethical	yes	1			
		10.5	14	75	

Question	Answer	Points	Relevant q	Overall quality	Options
1. Introduction: Were the	yes	1			yes
2. Methods: Was the sample	yes	1			partly
3. Methods: Was SEP	no/notstat	0			no/notstated
4. Methods: Were SEP-	partly	0.5			not relevant
5. Methods: Were	partly	0.5			
6. Methods: Were the SEP-	yes	1			
7. Methods: Was SEP	no/notstat	0			
8. Methods: Was missing	yes	1			
9. Methods: Were the	yes	1			
10. Results: Were the basic	partly	0.5			
11. Results: If appropriate,	not relevar	0			
12. Discussion: Were the	no/notstat	0			
13. Other: Were funding	no/notstat	0			
14. Other: Was ethical	not relevar	0			
		6.5	12	54.16667	

Pereira2023

Question	Answer	Points	Relevant q	Overall quality		Options
1. Introduction: Were the	yes	1				yes
2. Methods: Was the sample	yes	1				partly
3. Methods: Was SEP	yes	1				no/notstated
4. Methods: Were SEP-	yes	1				not relevant
5. Methods: Were	yes	1				
6. Methods: Were the SEP-	no/notstat	0				
7. Methods: Was SEP	no/notstat	0				
8. Methods: Was missing data	not relevar	0				
9. Methods: Were the	yes	1				
10. Results: Were the basic	no/notstat	0				
11. Results: If appropriate,	not relevar	0				
12. Discussion: Were the	yes	1				
13. Other: Were funding	partly	0.5				
14. Other: Was ethical	not relevar	0				
		7.5	11	68.18182		

Question	Answer	Points	Relevant q	Overall quality		Options
1. Introduction: Were the	yes	1				yes
2. Methods: Was the	yes	1				partly
3. Methods: Was SEP	yes	1				no/notstated
4. Methods: Were SEP-	yes	1				not relevant
5. Methods: Were	partly	0.5				
6. Methods: Were the SEP-	no/notstat	0				
7. Methods: Was SEP	no/notstat	0				
8. Methods: Was missing	not relevan	0				
9. Methods: Were the	yes	1				
10. Results: Were the basic	no/notstat	0				
11. Results: If appropriate,	not relevan	0				
12. Discussion: Were the	yes	1				
13. Other: Were funding	no/notstat	0				
14. Other: Was ethical	not relevan	0				
		6.5	11	59.09091		

Question	Answer	Points	Relevant q	Overall quality	Options
1. Introduction: Were the	yes	1			yes
2. Methods: Was the sample	yes	1			partly
3. Methods: Was SEP	yes	1			no/notstated
4. Methods: Were SEP-	yes	1			not relevant
5. Methods: Were	yes	1			
6. Methods: Were the SEP-	yes	1			
7. Methods: Was SEP analysed	yes	1			
8. Methods: Was missing data	yes	1			
9. Methods: Were the	yes	1			
10. Results: Were the basic	no/notstat	0			
11. Results: If appropriate, was	yes	1			
12. Discussion: Were the	yes	1			
13. Other: Were funding	yes	1			
14. Other: Was ethical	no/notstat	0			
		12	14	85.71429	

Richardson2010

Question	Answer	Points	Relevant q	Overall quality	Options
1. Introduction: Were the	yes	1			yes
2. Methods: Was the	yes	1			partly
3. Methods: Was SEP	yes	1			no/notstated
4. Methods: Were SEP-	yes	1			not relevant
5. Methods: Were	partly	0.5			
6. Methods: Were the	partly	0.5			
7. Methods: Was SEP	partly	0.5			
8. Methods: Was missing	no/notstat	0			
9. Methods: Were the	yes	1			
10. Results: Were the	no/notstat	0			
11. Results: If	not relevar	0			
12. Discussion: Were the	yes	1			
13. Other: Were funding	yes	1			
14. Other: Was ethical	no/notstat	0			
		8.5	13	65.38462	

Question	Answer	Points	Relevant q	Overall quality	Options
1. Introduction: Were the aims/objectives of the study clear?	yes	1			yes
2. Methods: Was the sample frame taken from an appropriate population or ecological	yes	1			partly
3. Methods: Was SEP measured with more than 1 single indicator?	no/notstat	0			no/notstated
4. Methods: Were SEP-variables adequately described or do the authors refer to other	yes	1			not relevant
5. Methods: Were environmental variables adequately described or do the authors refer	yes	1			
6. Methods: Were the SEP- and environmental exposures measured within the same	yes	1			
7. Methods: Was SEP analysed at individual level?	no/notstat	0			
8. Methods: Was missing data handling adequately described?	no/notstat	0			
9. Methods: Were the methods (including statistical methods) sufficiently described to	no/notstat	0			
10. Results: Were the basic data adequately described?	no/notstat	0			
11. Results: If appropriate, was information about non-responders described?	not relevar	0			
12. Discussion: Were the limitations of the study discussed?	yes	1			
13. Other: Were funding sources and conflicts of interest disclosed?	no/notstat	0			
14. Other: Was ethical approval or consent of participants attained?	not relevar	0			
		6	12	50	

Question	Answer	Points	Relevant q	Overall quality	Options
1. Introduction: Were the	yes	1			yes
2. Methods: Was the sample frame	yes	1			partly
3. Methods: Was SEP measured with	no/notstat	0			no/notstated
4. Methods: Were SEP-variables	yes	1			not relevant
5. Methods: Were environmental	partly	0.5			
6. Methods: Were the SEP- and	no/notstat	0			
7. Methods: Was SEP analysed at	yes	1			
8. Methods: Was missing data	no/notstat	0			
9. Methods: Were the methods	partly	0.5			
10. Results: Were the basic data	yes	1			
11. Results: If appropriate, was	no/notstat	0			
12. Discussion: Were the limitations	yes	1			
13. Other: Were funding sources and	yes	1			
14. Other: Was ethical approval or	yes	1			
		9	14	64.28571	

Schaeffer2019

Question	Answer	Points	Relevant q	Overall quality		Options
1. Introduction: Were the aims/objectives of the	yes	1				yes
2. Methods: Was the sample frame taken from an	yes	1				partly
3. Methods: Was SEP measured with more than 1	no/notstat	0				no/notstated
4. Methods: Were SEP-variables adequately described	yes	1				not relevant
5. Methods: Were environmental variables	yes	1				
6. Methods: Were the SEP- and environmental	yes	1				
7. Methods: Was SEP analysed at individual level?	no/notstat	0				
8. Methods: Was missing data handling adequately	not relevar	0				
9. Methods: Were the methods (including statistical	yes	1				
10. Results: Were the basic data adequately	partly	0.5				
11. Results: If appropriate, was information about	not relevar	0				
12. Discussion: Were the limitations of the study	no/notstat	0				
13. Other: Were funding sources and conflicts of	no/notstat	0				
14. Other: Was ethical approval or consent of	not relevar	0				
		6.5	11	59.09091		

Question	Answer	Points	Relevant q	Overall quality	Options
1. Introduction: Were the aims/objectives	yes	1			yes
2. Methods: Was the sample frame taken	yes	1			partly
3. Methods: Was SEP measured with	no/notstat	0			no/notstated
4. Methods: Were SEP-variables	yes	1			not relevant
5. Methods: Were environmental	yes	1			
6. Methods: Were the SEP- and	partly	0.5			
7. Methods: Was SEP analysed at	no/notstat	0			
8. Methods: Was missing data handling	not relevar	0			
9. Methods: Were the methods (including	yes	1			
10. Results: Were the basic data	yes	1			
11. Results: If appropriate, was	not relevar	0			
12. Discussion: Were the limitations of	yes	1			
13. Other: Were funding sources and	yes	1			
14. Other: Was ethical approval or	not relevar	0			
		8.5	11	77.27273	

Question	Answer	Points	Relevant q	Overall quality	Options
1. Introduction: Were the	yes	1			yes
2. Methods: Was the sample frame	yes	1			partly
3. Methods: Was SEP measured with	yes	1			no/notstated
4. Methods: Were SEP-variables	yes	1			not relevant
5. Methods: Were environmental	yes	1			
6. Methods: Were the SEP- and	no/notstat	0			
7. Methods: Was SEP analysed at	no/notstat	0			
8. Methods: Was missing data	not relevar	0			
9. Methods: Were the methods	yes	1			
10. Results: Were the basic data	partly	0.5			
11. Results: If appropriate, was	not relevar	0			
12. Discussion: Were the limitations	no/notstat	0			
13. Other: Were funding sources and	yes	1			
14. Other: Was ethical approval or	not relevar	0			
		7.5	11	68.18182	

**Question**	**Answer**	**Points**	**Relevant q**	**Overall quality**	**Options**
1. Introduction: Were the	yes	1			yes
2. Methods: Was the sample frame	yes	1			partly
3. Methods: Was SEP measured with	yes	1			no/notstated
4. Methods: Were SEP-variables	yes	1			not relevant
5. Methods: Were environmental	yes	1			
6. Methods: Were the SEP- and	partly	0.5			
7. Methods: Was SEP analysed at	no/notstat	0			
8. Methods: Was missing data handling	not relevar	0			
9. Methods: Were the methods	yes	1			
10. Results: Were the basic data	partly	0.5			
11. Results: If appropriate, was	not relevar	0			
12. Discussion: Were the limitations of	yes	1			
13. Other: Were funding sources and	yes	1			
14. Other: Was ethical approval or	not relevar	0			
		9	11	81.81818	

Question	Answer	Points	Relevant q	Overall quality	Options
1. Introduction: Were the aims/objectives of the study clear?	yes	1			yes
2. Methods: Was the sample frame taken from an appropriate population or ecological (e.g.	yes	1			partly
3. Methods: Was SEP measured with more than 1 single indicator?	no/notstat	0			no/notstated
4. Methods: Were SEP-variables adequately described or do the authors refer to other	yes	1			not relevant
5. Methods: Were environmental variables adequately described or do the authors refer to	yes	1			
6. Methods: Were the SEP- and environmental exposures measured within the same year	no/notstat	0			
7. Methods: Was SEP analysed at individual level?	no/notstat	0			
8. Methods: Was missing data handling adequately described?	not relevar	0			
9. Methods: Were the methods (including statistical methods) sufficiently described to	yes	1			
10. Results: Were the basic data adequately described?	partly	0.5			
11. Results: If appropriate, was information about non-responders described?	not relevar	0			
12. Discussion: Were the limitations of the study discussed?	yes	1			
13. Other: Were funding sources and conflicts of interest disclosed?	yes	1			
14. Other: Was ethical approval or consent of participants attained?	not relevar	0			
		7.5	11	68.18182	

Stucki2023

Question	Answer	Points	Relevant q	Overall quality		Options
1. Introduction: Were the aims/objectives	yes	1				yes
2. Methods: Was the sample frame taken	yes	1				partly
3. Methods: Was SEP measured with more	yes	1				no/notstated
4. Methods: Were SEP-variables adequately	yes	1				not relevant
5. Methods: Were environmental variables	yes	1				
6. Methods: Were the SEP- and	yes	1				
7. Methods: Was SEP analysed at individual	yes	1				
8. Methods: Was missing data handling	yes	1				
9. Methods: Were the methods (including	yes	1				
10. Results: Were the basic data	partly	0.5				
11. Results: If appropriate, was information	no/notstat	0				
12. Discussion: Were the limitations of the	yes	1				
13. Other: Were funding sources and	yes	1				
14. Other: Was ethical approval or consent	yes	1				
		12.5	14	89.28571		

Question	Answer	Points	Relevant q	Overall qu	Notes	Options
1. Introduction: Were the aims/objectives	yes	1				yes
2. Methods: Was the sample frame taken	yes	1				partly
3. Methods: Was SEP measured with	yes	1				no/notstated
4. Methods: Were SEP-variables	yes	1				not relevant
5. Methods: Were environmental	yes	1				
6. Methods: Were the SEP- and	yes	1				
7. Methods: Was SEP analysed at	yes	1				
8. Methods: Was missing data handling	no/notstat	0				
9. Methods: Were the methods (including	yes	1				
10. Results: Were the basic data	yes	1			In supplementary	
11. Results: If appropriate, was	no/notstat	0				
12. Discussion: Were the limitations of	yes	1				
13. Other: Were funding sources and	partly	0.5				
14. Other: Was ethical approval or	yes	1				
		11.5	14	82.14286		

Question	Answer	Points	Relevant q	Overall quality	Options
1. Introduction: Were the	yes	1			yes
2. Methods: Was the sample	yes	1			partly
3. Methods: Was SEP measured	no/notstat	0			no/notstated
4. Methods: Were SEP-variables	no/notstat	0			not relevant
5. Methods: Were environmental	yes	1			
6. Methods: Were the SEP- and	yes	1			
7. Methods: Was SEP analysed at	no/notstat	0			
8. Methods: Was missing data	not relevar	0			
9. Methods: Were the methods	yes	1			
10. Results: Were the basic data	partly	0.5			
11. Results: If appropriate, was	not relevar	0			
12. Discussion: Were the	yes	1			
13. Other: Were funding sources	yes	1			
14. Other: Was ethical approval	not relevar	0			
		7.5	11	68.18182	

Question	Answer	Points	Relevant q	Overall quality	Options
1. Introduction: Were the aims/objectives	partly	0.5			yes
2. Methods: Was the sample frame taken	yes	1			partly
3. Methods: Was SEP measured with	yes	1			no/notstated
4. Methods: Were SEP-variables	yes	1			not relevant
5. Methods: Were environmental	yes	1			
6. Methods: Were the SEP- and	yes	1			
7. Methods: Was SEP analysed at	no/notstat	0			
8. Methods: Was missing data handling	not relevar	0			
9. Methods: Were the methods (including	yes	1			
10. Results: Were the basic data	partly	0.5			
11. Results: If appropriate, was	not relevar	0			
12. Discussion: Were the limitations of	yes	1			
13. Other: Were funding sources and	yes	1			
14. Other: Was ethical approval or	not relevar	0			
		9	11	81.81818	

Question	Answer	Points	Relevant q	Overall quality		Options
1. Introduction: Were the	yes	1				yes
2. Methods: Was the sample frame	yes	1				partly
3. Methods: Was SEP measured with	yes	1				no/notstated
4. Methods: Were SEP-variables	yes	1				not relevant
5. Methods: Were environmental	yes	1				
6. Methods: Were the SEP- and	yes	1				
7. Methods: Was SEP analysed at	no/notstated	0				
8. Methods: Was missing data	yes	1				
9. Methods: Were the methods	partly	0.5				
10. Results: Were the basic data	yes	1				
11. Results: If appropriate, was	no/notstated	0				
12. Discussion: Were the limitations of	yes	1				
13. Other: Were funding sources and	partly	0.5				
14. Other: Was ethical approval or	yes	1				
		11	14	78.57143		

Question	Answer	Points	Relevant q	Overall quality	Options
1. Introduction: Were the	yes	1			yes
2. Methods: Was the sample frame	yes	1			partly
3. Methods: Was SEP measured with	no/notstated	0			no/notstated
4. Methods: Were SEP-variables	yes	1			not relevant
5. Methods: Were environmental	yes	1			
6. Methods: Were the SEP- and	yes	1			
7. Methods: Was SEP analysed at	no/notstated	0			
8. Methods: Was missing data	not relevant	0			
9. Methods: Were the methods	partly	0.5			
10. Results: Were the basic data	partly	0.5			
11. Results: If appropriate, was	not relevant	0			
12. Discussion: Were the limitations of	no/notstated	0			
13. Other: Were funding sources and	partly	0.5			
14. Other: Was ethical approval or	not relevant	0			
		6.5	11	59.09091	

Tiznado-Aitken2022

Question	Answer	Points	Relevant q	Overall quality	Options
1. Introduction: Were the	partly	0.5			yes
2. Methods: Was the sample frame	yes	1			partly
3. Methods: Was SEP measured	yes	1			no/notstated
4. Methods: Were SEP-variables	yes	1			not relevant
5. Methods: Were environmental	no/notstat	0			
6. Methods: Were the SEP- and	no/notstat	0			
7. Methods: Was SEP analysed at	no/notstat	0			
8. Methods: Was missing data	not relevar	0			
9. Methods: Were the methods	no/notstat	0			
10. Results: Were the basic data	partly	0.5			
11. Results: If appropriate, was	not relevar	0			
12. Discussion: Were the	no/notstat	0			
13. Other: Were funding sources	yes	1			
14. Other: Was ethical approval or	not relevar	0			
		5	11	45.45455	

Question	Answer	Points	Relevant q	Overall quality	Options
1. Introduction: Were the	yes	1			yes
2. Methods: Was the sample	yes	1			partly
3. Methods: Was SEP	yes	1			no/notstated
4. Methods: Were SEP-	partly	0.5			not relevant
5. Methods: Were	yes	1			
6. Methods: Were the SEP- and	yes	1			
7. Methods: Was SEP analysed	partly	0.5			
8. Methods: Was missing data	yes	1			
9. Methods: Were the	yes	1			
10. Results: Were the basic	partly	0.5			
11. Results: If appropriate, was	no/notstat	0			
12. Discussion: Were the	yes	1			
13. Other: Were funding	yes	1			
14. Other: Was ethical	yes	1			
		11.5	14	82.14286	

Question	Answer	Points	Relevant q	Overall quality		Options
1. Introduction: Were the	partly	0.5				yes
2. Methods: Was the sample frame	yes	1				partly
3. Methods: Was SEP measured with	no/notstated	0				no/notstated
4. Methods: Were SEP-variables	yes	1				not relevant
5. Methods: Were environmental	yes	1				
6. Methods: Were the SEP- and	yes	1				
7. Methods: Was SEP analysed at	yes	1				
8. Methods: Was missing data	no/notstated	0				
9. Methods: Were the methods	yes	1				
10. Results: Were the basic data	yes	1				
11. Results: If appropriate, was	partly	0.5				
12. Discussion: Were the limitations	yes	1				
13. Other: Were funding sources and	partly	0.5				
14. Other: Was ethical approval or	yes	1				
		10.5	14	75		

Question	Answer	Points	Relevant q	Overall quality		Options
1. Introduction: Were the	yes	1				yes
2. Methods: Was the sample frame taken	no/notstat	0				partly
3. Methods: Was SEP measured with	yes	1				no/notstated
4. Methods: Were SEP-variables	yes	1				not relevant
5. Methods: Were environmental	yes	1				
6. Methods: Were the SEP- and	yes	1				
7. Methods: Was SEP analysed at	partly	0.5	Data also collected for census block but the results are not reported, only used for adjusment			
8. Methods: Was missing data handling	yes	1				
9. Methods: Were the methods	partly	0.5				
10. Results: Were the basic data	partly	0.5				
11. Results: If appropriate, was	no/notstat	0				
12. Discussion: Were the limitations of	yes	1				
13. Other: Were funding sources and	yes	1				
14. Other: Was ethical approval or	yes	1				
		10.5	14	75		

Question	Answer	Points	Relevant q	Overall quality	Options
1. Introduction: Were the	yes	1			yes
2. Methods: Was the sample	yes	1			partly
3. Methods: Was SEP	no/notstat	0			no/notstated
4. Methods: Were SEP-	no/notstat	0			not relevant
5. Methods: Were	yes	1			
6. Methods: Were the SEP-	no/notstat	0			
7. Methods: Was SEP	no/notstat	0			
8. Methods: Was missing	not relevar	0			
9. Methods: Were the	partly	0.5			
10. Results: Were the basic	partly	0.5			
11. Results: If appropriate,	not relevar	0			
12. Discussion: Were the	no/notstat	0			
13. Other: Were funding	yes	1			
14. Other: Was ethical	not relevar	0			
		5	11	45.45455	

Question	Answer	Points	Relevant q	Overall quality	Options
1. Introduction: Were the aims/objectives of the study clear?	yes	1			yes
2. Methods: Was the sample frame taken from an appropriate	partly	0.5			partly
3. Methods: Was SEP measured with more than 1 single indicator?	no/notstat	0			no/notstated
4. Methods: Were SEP-variables adequately described or do the	yes	1			not relevant
5. Methods: Were environmental variables adequately described or	partly	0.5			
6. Methods: Were the SEP- and environmental exposures measured	no/notstat	0			
7. Methods: Was SEP analysed at individual level?	yes	1			
8. Methods: Was missing data handling adequately described?	yes	1			
9. Methods: Were the methods (including statistical methods)	yes	1			
10. Results: Were the basic data adequately described?	yes	1			
11. Results: If appropriate, was information about non-responders	no/notstat	0			
12. Discussion: Were the limitations of the study discussed?	yes	1			
13. Other: Were funding sources and conflicts of interest disclosed?	yes	1			
14. Other: Was ethical approval or consent of participants attained?	yes	1			
		10	14	71.42857	

Question	Answer	Points	Relevant q	Overall quality	Comments	Options
1.	yes	1				yes
2.	yes	1				partly
3.	yes	1				no/notstated
4.	yes	1				not relevant
5.	yes	1				
6.	yes	1				
7.	no/notstat	0			city block	
8.	no/notstat	0				
9.	yes	1				
10.	yes	1				
11.	not relevar	0			ecological data	
12.	yes	1				
13. Other:	no/notstat	0				
14. Other:	not relevar	0			publicly available data	
		9	12	75		

Question	Answer	Points	Relevant q	Overall quality	Comments	Options
1.	yes	1				yes
2.	yes	1				partly
3.	no/notstat	0				no/notstated
4.	yes	1				not relevant
5.	yes	1				
6.	no/notstat	0			Not clear from which year green space data is	
7.	no/notstat	0				
8.	no/notstat	0				
9.	partly	0.5			Only a very general description of statistical analysis was provided	
10.	partly	0.5			regression data described in a different way for each country. Also no tables on regression results provided	
11.	not relevar	0			ecological data	
12.	yes	1				
13. Other:	partly	0.5			Only conflicts of interest disclosed	
14. Other:	not relevar	0			publicly available data	
		6.5	12	54.16667		

Question	Answer	Points	Relevant q	Overall quality	Comments	Options
1.	yes	1				yes
2.	yes	1				partly
3.	yes	1				no/notstated
4.	yes	1				not relevant
5.	yes	1				
6.	no/notstat	0			Not clear in which year environmental data was collected	
7.	no/notstat	0			Neighbourhood	
8.	yes	1				
9.	yes	1				
10.	yes	1				
11.	yes	1				
12.	yes	1				
13. Other:	yes	1				
14. Other:	yes	1				
		12	14	85.71429		

Question	Answer	Points	Relevant q	Overall quality	Comments	Options
1.	yes	1				yes
2.	yes	1				partly
3.	yes	1				no/notstated
4.	yes	1				not relevant
5.	yes	1				
6.	yes	1				
7.	no/notstat	0			census tract level	
8.	no/notstat	0				
9.	yes	1				
10.	yes	1				
11.	not relevar	0			ecological data	
12.	yes	1				
13. Other:	yes	1				
14. Other:	not relevar	0			publicly available data	
		10	12	83.33333		

Question	Answer	Points	Relevant q	Overall quality	Comments	Options
1.	yes	1				yes
2.	yes	1				partly
3.	yes	1				no/notstated
4.	yes	1				not relevant
5.	yes	1				
6.	no/notstat	0			Not clear from which year SEP data is	
7.	no/notstat	0				
8.	yes	1				
9.	yes	1				
10.	yes	1				
11.	no/notstat	0				
12.	yes	1				
13. Other:	yes	1				
14. Other:	yes	1				
		11	14	78.57143		

Question	Answer	Points	Relevant q	Overall quality	Comments	Options
1.	yes	1				yes
2.	yes	1				partly
3.	no/notstat	0				no/notstated
4.	yes	1				not relevant
5.	yes	1				
6.	yes	1				
7.	no/notstat	0				
8.	no/notstat	0				
9.	yes	1				
10.	yes	1				
11.	not relevar	0			ecological data	
12.	yes	1				
13. Other:	no/notstat	0				
14. Other:	not relevar	0			publicly available data, no participants involved	
		8	12	66.66667		

Question	Answer	Points	Relevant q	Overall quality	Comments	Options
1.	yes	1				yes
2.	yes	1				partly
3.	yes	1				no/notstated
4.	yes	1				not relevant
5.	yes	1				
6.	yes	1				
7.	no/notstat	0				
8.	no/notstat	0				
9.	yes	1				
10.	partly	0.5			Descriptive table of mean NDVI values per SEP category not available. Only numerical results of lowest and highest SEP crossed with NDVI which are showed fig.6 available in text.	
11.	not relevar	0			ecological data	
12.	no/notstat	0				
13. Other:	yes	1				
14. Other:	not relevar	0			publicly available data, no participants involved	
		8.5	12	70.83333		

Question	Answer	Points	Relevant q	Overall quality	Comments	Options
1.	yes	1				yes
2.	yes	1				partly
3.	yes	1				no/notstated
4.	yes	1				not relevant
5.	yes	1				
6.	yes	1				
7.	no/notstat	0				
8.	no/notstat	0				
9.	yes	1				
10.	yes	1				
11.	not relevar	0			ecological data	
12.	yes	1				
13. Other:	yes	1				
14. Other:	not relevar	0			no participants involved	
		10	12	83.33333		

Question	Answer	Points	Relevant q	Overall quality	Comments	Options
1.	yes	1				yes
2.	yes	1				partly
3.	yes	1				no/notstated
4.	yes	1				not relevant
5.	yes	1				
6.	partly	0.5			For some variables data collection years not clear	
7.	yes	1				
8.	yes	1				
9.	yes	1				
10.	yes	1				
11.	no/notstat	0				
12.	yes	1				
13. Other:	yes	1				
14. Other:	yes	1				
		12.5	14	89.28571		

Question	Answer	Points	Relevant q	Overall quality	Comments	Options
1.	yes	1				yes
2.	yes	1				partly
3.	no/notstat	0				no/notstated
4.	yes	1				not relevant
5.	no/notstat	0			Access to parks not clearly described, other refs needed to understand	
6.	no/notstat	0			data collection years parks not clear	
7.	no/notstat	0				
8.	no/notstat	0				
9.	no/notstat	0			Methodology lacks essential details to be able to be repeated	
10.	partly	0.5			Not clear about what unit the n=1,481 is (which is an essential part of the descriptive table)	
11.	not relevar	0			ecological data	
12.	yes	1				
13. Other:	yes	1				
14. Other:	not relevar	0			publicly available data, no participants involved	
		5.5	12	45.83333		

Question	Answer	Points	Relevant q	Overall quality	Comments	Options
1.	yes	1				yes
2.	yes	1				partly
3.	yes	1				no/notstated
4.	partly	0.5			Not all indicators of deprivation in	not relevant
5.	yes	1				
6.	yes	1				
7.	yes	1				
8.	no/notstat	0				
9.	no/notstat	0			Statistical analysis performed not described in detail	
10.	no/notstat	0			No table with descriptives/summary statistics provided	
11.	not relevan	0			Routinely collected data from health records, social services, and other administrative datasets	
12.	yes	1				
13. Other:	yes	1				
14. Other:	no/notstat	0			No ethics statement made	
		8.5	13	65.38462		

Question	Answer	Points	Relevant q	Overall quality	Comments	Options
1.	yes	1				yes
2.	yes	1				partly
3.	yes	1				no/notstated
4.	yes	1				not relevant
5.	yes	1				
6.	partly	0.5			Most SEP variables 2001, income 2005, NDVI 2006	
7.	yes	1				
8.	yes	1				
9.	yes	1				
10.	yes	1				
11.	not relevar	0			Administrative data used	
12.	yes	1				
13. Other:	yes	1				
14. Other:	no/notstat	0			No ethics statement made	
		11.5	13	88.46154		

Question	Answer	Points	Relevant q	Overall quality	Comments	Options
1.	yes	1				yes
2.	yes	1				partly
3.	yes	1				no/notstated
4.	yes	1				not relevant
5.	yes	1				
6.	no/notstat	0			No references provided for some data sources of built environment variables	
7.	no/notstat	0				
8.	no/notstat	0			Only missing data handling of individual level data (not used in this review) described	
9.	partly	0.5			Little information about built environment variables	
10.	yes	1				
11.	not relevar	0			ecological data	
12.	yes	1				
13. Other:	partly	0.5			conflicts of interest not disclosed	
14. Other:	not relevar	0			publicly available data	
		8	12	66.66667		

Question	Answer	Points	Relevant q	Overall quality	Comments	Options
1.	yes	1				yes
2.	yes	1				partly
3.	no/notstat	0				no/notstated
4.	yes	1				not relevant
5.	no/notstat	0			Not clear how/with which data source the walkability indicatores of 2 neighbourhoods were assessed, and what was the cut-off point for high/low walkability.	
6.	no/notstat	0			Data collection years not clear	
7.	yes	1				
8.	yes	1				
9.	no/notstat	0			Description of high/low walkable neighbourhood too vague to me repeated. Also not clear which test was performed to assess differences in education level	
10.	partly	0.5			Not clear from which test the P-values are retrieved, but rest of the descriptive table is clear	
11.	yes	1				
12.	yes	1				
13. Other:	partly	0.5			Conflicts of interest not disclosed	
14. Other:	yes	1				
		9	14	64.28571		

Saporito2015

Question	Answer	Points	Relevant q	Overall quality	Comments	Options
1.	yes	1				yes
2.	yes	1				partly
3.	no/notstat	0				no/notstated
4.	yes	1				not relevant
5.	yes	1				
6.	partly	0.5			2008-2012 and 2012	
7.	no/notstat	0				
8.	no/notstat	0				
9.	yes	1				
10.	yes	1				
11.	not relevar	0			ecological data	
12.	yes	1				
13. Other:	no/notstat	0				
14. Other:	not relevar	0			publicly available data, no participants involved	
		7.5	12	62.5		

Scheurer2019

Question	Answer	Points	Relevant q	Overall quality	Comments	Options
1.	yes	1				yes
2.	yes	1				partly
3.	yes	1				no/notstated
4.	yes	1				not relevant
5.	yes	1				
6.	no/notstat	0			Data collection years SEIFA not clear	
7.	no/notstat	0				
8.	no/notstat	0				
9.	yes	1				
10.	yes	1				
11.	not relevar	0			ecological data	
12.	no/notstat	0				
13. Other:	partly	0.5			conflicts of interest not disclosed	
14. Other:	not relevar	0			publicly available data, no participants involved	
		7.5	12	62.5		

Scheurer2017

Question	Answer	Points	Relevant q	Overall quality	Comments	Options
1.	yes	1				yes
2.	yes	1				partly
3.	yes	1				no/notstated
4.	yes	1				not relevant
5.	yes	1				
6.	yes	1				
7.	no/notstat	0				
8.	no/notstat	0				
9.	yes	1				
10.	yes	1				
11.	not relevar	0			ecological data	
12.	no/notstat	0				
13. Other:	partly	0.5			conflicts of interest not disclosed	
14. Other:	not relevar	0			publicly available data, no participants involved	
		8.5	12	70.83333		

Question	Answer	Points	Relevant q	Overall quality	Comments	Options
1.	yes	1				yes
2.	yes	1				partly
3.	yes	1				no/notstated
4.	yes	1				not relevant
5.	yes	1				
6.	yes	1				
7.	no/notstat	0				
8.	no/notstat	0				
9.	yes	1				
10.	yes	1				
11.	not relevar	0			ecological data	
12.	yes	1				
13. Other:	yes	1				
14. Other:	not relevar	0			no participants involved	
		10	12	83.33333		

Question	Answer	Points	Relevant q	Overall quality	Comments	Options
1.	yes	1				yes
2.	yes	1				partly
3.	no/notstat	0				no/notstated
4.	yes	1				not relevant
5.	yes	1				
6.	yes	1				
7.	no/notstat	0				
8.	no/notstat	0				
9.	yes	1				
10.	yes	1				
11.	not relevar	0			ecological data	
12.	yes	1				
13. Other:	partly	0.5			conflicts of interest not disclosed	
14. Other:	yes	1				
		9.5	13	73.07692		

Question	Answer	Points	Relevant q	Overall quality	Comments	Options
1.	yes	1				yes
2.	yes	1				partly
3.	yes	1				no/notstated
4.	yes	1				not relevant
5.	yes	1				
6.	partly	0.5			SEP variables between 2009 and 2015, greenspace variables in 2015	
7.	no/notstat	0				
8.	no/notstat	0				
9.	yes	1				
10.	yes	1				
11.	not relevar	0			ecological data	
12.	yes	1				
13. Other:	yes	1				
14. Other:	not relevar	0			publicly available data, no participants involved	
		9.5	12	79.16667		

Timperio2007

Question	Answer	Points	Relevant q	Overall quality	Comments	Options
1.	yes	1				yes
2.	yes	1				partly
3.	yes	1				no/notstated
4.	yes	1				not relevant
5.	yes	1				
6.	yes	1				
7.	no/notstat	0				
8.	no/notstat	0				
9.	yes	1				
10.	yes	1				
11.	not relevar	0			ecological data	
12.	yes	1				
13. Other:	partly	0.5			conflicts of interest not disclosed	
14. Other:	not relevar	0			publicly available data, no participants involved	
		9.5	12	79.16667		

Question	Answer	Points	Relevant q	Overall quality	Comments	Options
1.	yes	1				yes
2.	yes	1				partly
3.	yes	1				no/notstated
4.	yes	1				not relevant
5.	yes	1				
6.	no/notstat	0			data collection years exposure variables not clear	
7.	no/notstat	0				
8.	yes	1				
9.	no/notstat	0			Statistical analysis section not clearly described	
10.	yes	1				
11.	not relevar	0			ecological data	
12.	yes	1				
13. Other:	partly	0.5			conflicts of interest not disclosed	
14. Other:	not relevar	0			publicly available data, no participants involved	
		8.5	12	70.83333		

Question	Answer	Points	Relevant q	Overall quality	Comments	Options
1.	yes	1				yes
2.	yes	1				partly
3.	no/notstat	0				no/notstated
4.	partly	0.5			No reference to 2000 US census p	not relevant
5.	yes	1				
6.	no/notstat	0			SEP 2000, environment 2009-2010	
7.	yes	1				
8.	no/notstat	0				
9.	yes	1				
10.	yes	1				
11.	no/notstat	0				
12.	yes	1				
13. Other:	partly	0.5			conflicts of interest not disclosed	
14. Other:	yes	1				
		9	14	64.28571		

VanVelzen2023

Question	Answer	Points	Relevant q	Overall quality	Comments	Options
1.	yes	1				yes
2.	yes	1				partly
3.	yes	1				no/notstated
4.	yes	1				not relevant
5.	yes	1				
6.	yes	1				
7.	no/notstat	0				
8.	yes	1				
9.	yes	1				
10.	yes	1				
11.	not relevan	0			ecological data	
12.	yes	1				
13. Other:	partly	0.5			funding sources not disclosed	
14. Other:	not relevan	0			publicly available data, no participants involved	
		10.5	12	87.5		

Question	Answer	Points	Relevant q	Overall quality	Comments	Options
1.	yes	1				yes
2.	yes	1				partly
3.	no/notstat	0				no/notstated
4.	yes	1				not relevant
5.	yes	1				
6.	no/notstat	0			2005-2009 and 2011	
7.	no/notstat	0				
8.	partly	0.5			from the descriptive table you can see that missing data were removed, but missing data handling not mentioned in methods section	
9.	yes	1				
10.	yes	1				
11.	not relevar	0			ecological data	
12.	yes	1				
13. Other:	partly	0.5			conflicts of interest not disclosed	
14. Other:	not relevar	0			publicly available data, no participants involved	
		8	12	66.66667		

Question	Answer	Points	Relevant q	Overall quality	Comments	Options
1.	yes	1				yes
2.	yes	1				partly
3.	yes	1				no/notstated
4.	yes	1				not relevant
5.	yes	1				
6.	partly	0.5			SEP 2008-2019, NDVI and blue spaces 2020	
7.	no/notstat	0				
8.	no/notstat	0				
9.	partly	0.5			residential areas' from which the distance to blue spaces was measured is not described in more detail	
10.	no/notstat	0			no descriptive/summary table provided	
11.	not relevar	0			ecological data	
12.	no/notstat	0				
13. Other:	yes	1				
14. Other:	not relevar	0			publicly available data, no participants involved	
		7	12	58.33333		

Villanueva2016

Question	Answer	Points	Relevant q	Overall quality	Comments	Options
1.	yes	1				yes
2.	yes	1				partly
3.	yes	1				no/notstated
4.	yes	1				not relevant
5.	yes	1				
6.	no/notstat	0			2001 and 2005	
7.	yes	1				
8.	no/notstat	0				
9.	yes	1				
10.	partly	0.5			Unit of measurement descriptive table not reported	
11.	no/notstat	0				
12.	yes	1				
13. Other:	yes	1				
14. Other:	no/notstat	0				
		9.5	14	67.85714		

Wang2019

Question	Answer	Points	Relevant q	Overall quality	Comments	Options
1.	yes	1				yes
2.	yes	1				partly
3.	no/notstat	0				no/notstated
4.	yes	1				not relevant
5.	yes	1				
6.	no/notstat	0			Data collection NDVI not clear	
7.	no/notstat	0				
8.	no/notstat	0				
9.	no/notstat	0			Not reported what test was used to retrieve p-values from descriptive table	
10.	yes	1				
11.	not relevan	0			Register data	
12.	yes	1				
13. Other:	yes	1				
14. Other:	yes	1				
		8	13	61.53846		

Question	Answer	Points	Relevant q	Overall quality	Comments	Options
1.	yes	1				yes
2.	yes	1				partly
3.	yes	1				no/notstated
4.	yes	1				not relevant
5.	yes	1				
6.	yes	1				
7.	no/notstat	0				
8.	no/notstat	0				
9.	yes	1				
10.	yes	1				
11.	not relevan	0			ecological data	
12.	yes	1				
13. Other:	no/notstat	0				
14. Other:	not relevan	0			publicly available data, no participants involved	
		9	12	75		

Question	Answer	Points	Relevant q	Overall quality	Comments	Options
1.	yes	1				yes
2.	yes	1				partly
3.	no/notstat	0				no/notstated
4.	yes	1				not relevant
5.	yes	1				
6.	partly	0.5			SEP 2014, PA index variables collected between 2010 and 2017	
7.	no/notstat	0				
8.	no/notstat	0				
9.	yes	1				
10.	yes	1				
11.	not relevar	0			Ecological data	
12.	yes	1				
13. Other:	yes	1				
14. Other:	not relevar	0			publicly available data, no participants involved	
		8.5	12	70.83333		

Question	Answer	Points	Relevant q	Overall quality	Comments	Options
1.	yes	1				yes
2.	yes	1				partly
3.	no/notstat	0				no/notstated
4.	partly	0.5			No reference of ACS provided	not relevant
5.	yes	1				
6.	partly	0.5			SEP 2006-2010, distance to parks 2010, 2011, % vegetated land 2006	
7.	no/notstat	0				
8.	yes	1				
9.	yes	1				
10.	yes	1				
11.	not relevar	0			ecological data	
12.	yes	1				
13. Other:	yes	1				
14. Other:	not relevar	0			publicly available data, no participants involved	
		9	12	75		

Question	Answer	Points	Relevant q	Overall quality	Comments	Options
1.	yes	1				yes
2.	yes	1				partly
3.	yes	1				no/notstated
4.	partly	0.5			Not all variables included in the N;	not relevant
5.	yes	1				
6.	no/notstat	0				
7.	no/notstat	0				
8.	no/notstat	0				
9.	no/notstat	0				
10.	yes	1				
11.	not relevar	0			ecological data	
12.	yes	1				
13. Other:	partly	0.5			conflicts of interest not declared	
14. Other:	not relevar	0			publicly available data, no participants involved	
		7	12	58.33333		

Question	Answer	Points	Relevant q	Overall quality	Comments	Options
1.	yes	1				yes
2.	yes	1				partly
3.	yes	1				no/notstated
4.	partly	0.5			no reference provided for income	not relevant
5.	yes	1				
6.	no/notstat	0			Not clear when park data was collected	
7.	no/notstat	0				
8.	no/notstat	0				
9.	yes	1				
10.	yes	1				
11.	not relevan	0			ecological data	
12.	no/notstat	0				
13. Other:	partly	0.5			conflicts of interest not disclosed	
14. Other:	not relevan	0			publicly available data, no participants involved	
		7	12	58.33333		

Question	Answer	Points	Relevant q	Overall quality	Comments	Options
1.	yes	1				yes
2.	yes	1				partly
3.	yes	1				no/notstated
4.	partly	0.5			income and education not definec	not relevant
5.	yes	1				
6.	yes	1				
7.	yes	1				
8.	yes	1				
9.	yes	1				
10.	yes	1				
11.	no/notstat	0				
12.	yes	1				
13. Other:	partly	0.5			conflicts of interest not disclosed	
14. Other:	no/notstat	0				
		11	14	78.57143		

Question	Answer	Points	Relevant q	Overall quality	Comments	Options
1.	yes	1				yes
2.	yes	1				partly
3.	yes	1				no/notstated
4.	yes	1				not relevant
5.	yes	1				
6.	yes	1				
7.	no/notstat	0			school neighbourhood level	
8.	no/notstat	0				
9.	yes	1				
10.	partly	0.5			no descriptives on total sample	
11.	not relevar	0			ecological data	
12.	no/notstat	0				
13. Other:	no/notstat	0				
14. Other:	not relevar	0			publicly available data, no participants involved	
		7.5	12	62.5		

Question	Answer	Points	Relevant q	Overall quality	Comments	Options
1.	yes	1				yes
2.	yes	1				partly
3.	no/notstat	0				no/notstated
4.	yes	1				not relevant
5.	yes	1				
6.	yes	1				
7.	no/notstat	0				
8.	no/notstat	0				
9.	yes	1				
10.	partly	0.5			number of census areas and people living within those areas missing	
11.	yes	1				
12.	yes	1				
13. Other:	yes	1				
14. Other:	yes	1				
		10.5	14	75		

Question	Answer	Points	Relevant q	Overall quality	Comments	Options
1.	yes	1				yes
2.	yes	1				partly
3.	yes	1				no/notstated
4.	partly	0.5			Interpretation deprivation index n	not relevant
5.	yes	1				
6.	partly	0.5			Income 2010, Deprivation index data collection year not stated, NDVI data collection dependent of year of enrolment participant (between 2009 and 2014)	
7.	yes	1				
8.	yes	1				
9.	yes	1				
10.	yes	1				
11.	no/notstat	0				
12.	yes	1				
13. Other:	yes	1				
14. Other:	yes	1				
		12	14	85.71429		

Question	Answer	Points	Relevant q	Overall quality	Comments	Options
1.	yes	1				yes
2.	yes	1				partly
3.	yes	1				no/notstated
4.	yes	1				not relevant
5.	yes	1				
6.	yes	1				
7.	yes	1				
8.	yes	1				
9.	yes	1				
10.	yes	1				
11.	no/notstat	0				
12.	yes	1				
13. Other:	yes	1				
14. Other:	no/notstat	0				
		12	14	85.71429		

Question	Answer	Points	Relevant q	Overall quality	Comments	Options
1.	yes	1				yes
2.	yes	1				partly
3.	no/notstat	0				no/notstated
4.	yes	1				not relevant
5.	yes	1				
6.	partly	0.5			SEP 2010, environmental variables between 2008 and 2010	
7.	no/notstat	0				
8.	no/notstat	0				
9.	partly	0.5			Statistical methods only described very shortly without details	
10.	yes	1				
11.	not relevar	0			ecological data	
12.	yes	1				
13. Other:	partly	0.5			Funding sources not disclosed	
14. Other:	not relevar	0			Publicly available data, no participants involved	
		7.5	12	62.5		

Question	Answer	Points	Relevant q	Overall quality	Comments	Options
1.	yes	1				yes
2.	yes	1				partly
3.	yes	1				no/notstated
4.	partly	0.5			No reference of IMD provided	not relevant
5.	yes	1				
6.	no/notstat	0			Data collection years not clear	
7.	yes	1				
8.	yes	1				
9.	yes	1				
10.	yes	1				
11.	no/notstat	0				
12.	yes	1				
13. Other:	yes	1				
14. Other:	no/notstat	0				
		10.5	14	75		

Zhang2023a

Question	Answer	Points	Relevant q	Overall quality	Comments	Options
1.	yes	1				yes
2.	yes	1				partly
3.	yes	1				no/notstated
4.	yes	1				not relevant
5.	yes	1				
6.	no/notstat	0			Data collection years park proximity not clear	
7.	no/notstat	0				
8.	no/notstat	0				
9.	yes	1				
10.	partly	0.5			No summary/descriptive table provided	
11.	not relevar	0			ecological data	
12.	yes	1				
13. Other:	partly	0.5			funding sources not disclosed	
14. Other:	not relevar	0			publicly available data, no participants involved	
		8	12	66.66667		

Zhang2021

Question	Answer	Points	Relevant q	Overall quality	Comments	Options
1.	yes	1				yes
2.	yes	1				partly
3.	yes	1				no/notstated
4.	yes	1				not relevant
5.	yes	1				
6.	yes	1				
7.	no/notstat	0				
8.	no/notstat	0				
9.	yes	1				
10.	yes	1				
11.	not relevan	0			ecological dta	
12.	yes	1				
13. Other:	yes	1				
14. Other:	not relevan	0			publicly available data, no participants involved	
		10	12	83.33333		

Question	Answer	Points	Relevant q	Overall quality	Comments	Options
1.	yes	1				yes
2.	yes	1				partly
3.	yes	1				no/notstated
4.	yes	1				not relevant
5.	yes	1				
6.	yes	1				
7.	yes	1				
8.	no/notstat	0				
9.	yes	1				
10.	yes	1				
11.	no/notstat	0				
12.	yes	1				
13. Other:	yes	1				
14. Other:	yes	1				
		12	14	85.71429		

Question	Answer	Points	Relevant q	Overall quality	Comments	Options
1.	yes	1				yes
2.	yes	1				partly
3.	yes	1				no/notstated
4.	partly	0.5			Variables included in Nzdep not re	not relevant
5.	yes	1				
6.	yes	1				
7.	yes	1				
8.	yes	1				
9.	yes	1				
10.	yes	1				
11.	no/notstat	0				
12.	yes	1				
13. Other:	yes	1				
14. Other:	yes	1				
		12.5	14	89.28571		

Question	Answer	Points	Relevant q	Overall quality	Comments	Options
1.	yes	1				yes
2.	yes	1				partly
3.	no/notstat	0				no/notstated
4.	partly	0.5			No reference provided for poverty	not relevant
5.	yes	1				
6.	no/notstat	0			Data collection years not clear	
7.	no/notstat	0				
8.	no/notstat	0				
9.	no/notstat	0			Too little information on SEP variable to be able to be repeated	
10.	yes	1				
11.	not relevan	0			ecological data	
12.	yes	1				
13. Other:	yes	1				
14. Other:	not relevan	0			no participants involved	
		6.5	12	54.16667		

Question	Answer	Points	Relevant q	Overall quality	Comments	Options
1.	yes	1				yes
2.	yes	1				partly
3.	no/notstat	0				no/notstated
4.	partly	0.5			No reference for ACS provided	not relevant
5.	yes	1				
6.	partly	0.5			SEP 2006-2010, Walk Score 2010	
7.	no/notstat	0				
8.	partly	0.5			Authors mention "The final combined sample from the three sources with complete data included 115 neighborhoods" which implies that incomplete data were removed	
9.	yes	1				
10.	yes	1				
11.	not relevar	0			ecological data	
12.	no/notstat	0				
13. Other:	yes	1				
14. Other:	not relevar	0			publicly available data, no participants involved	
		7.5	12	62.5		

Question	Answer	Points	Relevant q	Overall quality	Comments	Options
1.	yes	1				yes
2.	yes	1				partly
3.	yes	1				no/notstated
4.	yes	1				not relevant
5.	yes	1				
6.	yes	1				
7.	no/notstat	0				
8.	no/notstat	0				
9.	no/notstat	0			Not described why certain variables were included in the regression model and others were not. Also not described how unit of analysis was aggregated in order to analyse on level of communit	
10.	yes	1				
11.	not relevar	0			ecological data	
12.	yes	1				
13. Other:	partly	0.5			funding sources not disclosed	
14. Other:	not relevar	0			no participants involved	
		8.5	12	70.83333		

ty

Question	Answer	Points	Relevant q	Overall quality	Comments	Options
1.	yes	1				yes
2.	yes	1				partly
3.	no/notstat	0				no/notstated
4.	partly	0.5			Short description of SEP variables	not relevant
5.	partly	0.5			No reference provided	
6.	no/notstat	0			Data collection years park variable not clear	
7.	no/notstat	0				
8.	no/notstat	0				
9.	yes	1				
10.	yes	1				
11.	not relevar	0			ecological data	
12.	no/notstat	0				
13. Other:	yes	1				
14. Other:	not relevar	0			publicly available data, no participants involved	
		6	12	50		

Question	Answer	Points	Relevant q	Overall quality	Comments	Options
1.	yes	1				yes
2.	yes	1				partly
3.	no/notstat	0				no/notstated
4.	yes	1				not relevant
5.	yes	1				
6.	partly	0.5			SEP 2014-2018, NDVI 2017-2018, parks not stated	
7.	no/notstat	0				
8.	no/notstat	0				
9.	yes	1				
10.	yes	1				
11.	not relevar	0			ecological data	
12.	no/notstat	0				
13. Other:	yes	1				
14. Other:	not relevar	0			publicly available data, no participants involved	
		7.5	12	62.5		

Question	Answer	Points	Relevant q	Overall quality	Comments	Options
1.	yes	1				yes
2.	yes	1				partly
3.	yes	1				no/notstated
4.	yes	1				not relevant
5.	yes	1				
6.	no/notstat	0			Eligibility for free lunch 2019, SVI 2018, land cover data 2014 and 2016	
7.	yes	1				
8.	no/notstat	0				
9.	yes	1				
10.	yes	1				
11.	not relevar	0			ecological data	
12.	yes	1				
13. Other:	yes	1				
14. Other:	not relevar	0			publicly available data, no participants involved	
		10	12	83.33333		

Question	Answer	Points	Relevant q	Overall quality	Comments	Options
1.	yes	1				yes
2.	yes	1				partly
3.	yes	1				no/notstated
4.	yes	1				not relevant
5.	yes	1				
6.	no/notstat	0			Data collection years walkable amenities not clear	
7.	yes	1				
8.	yes	1				
9.	yes	1				
10.	yes	1				
11.	yes	1				
12.	yes	1				
13. Other:	yes	1				
14. Other:	yes	1				
		13	14	92.85714		

Question	Answer	Points	Relevant q	Overall quality	Comments	Options
1.	yes	1				yes
2.	yes	1				partly
3.	yes	1				no/notstated
4.	no/notstat	0			No description of income- and education variables provided	not relevant
5.	partly	0.5			No references provided	
6.	yes	1				
7.	no/notstat	0				
8.	no/notstat	0				
9.	no/notstat	0			Many details of the methods are missing in order to be able to repeat them	
10.	partly	0.5				
11.	not relevar	0			ecological data	
12.	no/notstat	0				
13. Other:	partly	0.5			conflicts of interest not disclosed	
14. Other:	not relevar	0			no participants involved	
		5.5	12	45.83333		

Choi2020

Question	Answer	Points	Relevant q	Overall quality	Comments	Options
1.	yes	1				yes
2.	yes	1				partly
3.	yes	1				no/notstated
4.	yes	1				not relevant
5.	yes	1				
6.	no/notstat	0			2018 and 2014-2016	
7.	no/notstat	0				
8.	no/notstat	0				
9.	yes	1				
10.	yes	1				
11.	not relevar	0			ecological data	
12.	yes	1				
13. Other:	yes	1				
14. Other:	not relevar	0			publicly available data	
		9	12	75		

Question	Answer	Points	Relevant q	Overall quality	Comments	Options
1.	yes	1				yes
2.	yes	1				partly
3.	no/notstat	0				no/notstated
4.	yes	1				not relevant
5.	yes	1				
6.	yes	1				
7.	yes	1				
8.	yes	1				
9.	yes	1				
10.	yes	1				
11.	yes	1				
12.	yes	1				
13. Other:	yes	1				
14. Other:	yes	1				
		13	14	92.85714		

Question	Answer	Points	Relevant q	Overall quality	Comments	Options
1.	yes	1				yes
2.	yes	1				partly
3.	yes	1				no/notstated
4.	yes	1				not relevant
5.	yes	1				
6.	no/notstat	0			1990 and 2000-2001	
7.	no/notstat	0				
8.	no/notstat	0				
9.	yes	1				
10.	yes	1				
11.	not relevan	0			ecological data	
12.	yes	1				
13. Other:	yes	1				
14. Other:	not relevan	0			publicly available data	
		9	12	75		

Question	Answer	Points	Relevant q	Overall quality	Comments	Options
1.	yes	1				yes
2.	yes	1				partly
3.	yes	1				no/notstated
4.	yes	1				not relevant
5.	yes	1				
6.	yes	1				
7.	no/notstat	0				
8.	no/notstat	0				
9.	yes	1				
10.	partly	0.5			No descriptive table provided	
11.	not relevar	0			ecological data	
12.	yes	1				
13. Other:	yes	1				
14. Other:	not relevar	0			publicly available data	
		9.5	12	79.16667		

Question	Answer	Points	Relevant q	Overall quality	Comments	Options
1.	yes	1				yes
2.	yes	1				partly
3.	no/notstat	0				no/notstated
4.	yes	1				not relevant
5.	yes	1				
6.	no/notstat	0			Not clear when environmental exposures were measured	
7.	no/notstat	0				
8.	no/notstat	0				
9.	partly	0.5				
10.	yes	1				
11.	not relevan	0			ecological data	
12.	yes	1				
13. Other:	yes	1				
14. Other:	yes	1				
		8.5	13	65.38462		

Question	Answer	Points	Relevant q	Overall quality	Comments	Options
1.	yes	1				yes
2.	yes	1				partly
3.	no/notstat	0				no/notstated
4.	yes	1				not relevant
5.	yes	1				
6.	yes	1				
7.	no/notstat	0				
8.	yes	1				
9.	yes	1				
10.	yes	1				
11.	not relevar	0			ecological data	
12.	yes	1				
13. Other:	yes	1				
14. Other:	not relevar	0				
		10	12	83.33333		

Question	Answer	Points	Relevant q	Overall quality	Comments	Options
1.	yes	1				yes
2.	yes	1				partly
3.	no/notstat	0			Only income	no/notstated
4.	no/notstat	0			No description about income varia	not relevant
5.	partly	0.5				
6.	no/notstat	0			Year(s) of data collection not clear	
7.	no/notstat	0				
8.	no/notstat	0				
9.	partly	0.5				
10.	yes	1				
11.	not relevar	0			ecological data	
12.	yes	1				
13. Other:	partly	0.5			Conflicts of interest not mentioned	
14. Other:	yes	1				
		6.5	13	50		

Question	Answer	Points	Relevant q	Overall quality	Comments	Options
1.	yes	1				yes
2.	yes	1				partly
3.	yes	1				no/notstated
4.	no/notstat	0			Per capita income not further desc	not relevant
5.	yes	1				
6.	yes	1				
7.	no/notstat	0				
8.	no/notstat	0				
9.	partly	0.5				
10.	partly	0.5			No descriptives provided	
11.	not relevar	0			ecological data	
12.	no/notstat	0				
13. Other:	no/notstat	0				
14. Other:	not relevar	0				
		6	12	50		

Question	Answer	Points	Relevant q	Overall quality	Comments	Options
1.	yes	1				yes
2.	yes	1				partly
3.	yes	1				no/notstated
4.	yes	1				not relevant
5.	yes	1				
6.	no/notstat	0				
7.	yes	1				
8.	yes	1				
9.	yes	1				
10.	yes	1				
11.	no/notstat	0				
12.	yes	1				
13. Other:	partly	0.5				
14. Other:	yes	1				
		11.5	14	82.14286		

Question	Answer	Points	Relevant q	Overall quality	Comments	Options
1.	yes	1				yes
2.	yes	1				partly
3.	yes	1				no/notstated
4.	yes	1				not relevant
5.	yes	1				
6.	no/notstat	0			Data collection year environmental variables not clear	
7.	no/notstat	0			Census tract	
8.	yes	1				
9.	yes	1				
10.	yes	1				
11.	not relevar	0			ecological data	
12.	no/notstat	0				
13. Other:	partly	0.5			conflicts of interest not declared	
14. Other:	not relevar	0			publicly available data	
		8.5	12	70.83333		

DelaBarrera2016

Question	Answer	Points	Relevant q	Overall quality	Comments	Options
1.	yes	1				yes
2.	yes	1				partly
3.	no/notstat	0				no/notstated
4.	no/notstat	0			no further description of municipa	not relevant
5.	yes	1				
6.	no/notstat	0			data collection year of income variable not clear	
7.	no/notstat	0			municipality level	
8.	no/notstat	0				
9.	partly	0.5				
10.	yes	1				
11.	not relevar	0			ecological	
12.	no/notstat	0				
13. Other:	partly	0.5			conflicts of interest not declared	
14. Other:	not relevar	0				
		5	12	41.66667		

Question	Answer	Points	Relevant q	Overall quality	Comments	Options
1.	yes	1				yes
2.	yes	1				partly
3.	no/notstat	0				no/notstated
4.	yes	1				not relevant
5.	yes	1				
6.	no/notstat	0			2005 and 2008	
7.	no/notstat	0				
8.	no/notstat	0				
9.	yes	1				
10.	partly	0.5			Summary statistics only given in tekst, not in descriptive table	
11.	not relevar	0				
12.	yes	1				
13. Other:	partly	0.5			conflicts of interest not disclosed	
14. Other:	not relevar	0				
		7	12	58.33333		

Question	Answer	Points	Relevant q	Overall quality	Comments	Options
1.	yes	1				yes
2.	yes	1				partly
3.	no/notstat	0			Income	no/notstated
4.	yes	1				not relevant
5.	yes	1				
6.	yes	1				
7.	no/notstat	0			Census block level	
8.	no/notstat	0			Not mentioned	
9.	yes	1				
10.	yes	1				
11.	not relevar	0				
12.	yes	1				
13. Other:	yes	1				
14. Other:	yes	1				
		10	13	76.92308		

DelaBarrera2019

Question	Answer	Points	Relevant q	Overall quality	Comments	Options
1.	yes	1				yes
2.	yes	1				partly
3.	no/notstat	0				no/notstated
4.	no/notstat	0				not relevant
5.	yes	1				
6.	no/notstat	0				
7.	no/notstat	0				
8.	no/notstat	0				
9.	no/notstat	0				
10.	yes	1				
11.	not relevar	0				
12.	no/notstat	0				
13. Other:	partly	0.5				
14. Other:	not relevar	0				
		4.5	12	37.5		

note: this was a conference paper

Question	Answer	Points	Relevant q	Overall quality	Comments	Options
1.	yes	1				yes
2.	yes	1				partly
3.	yes	1				no/notstated
4.	yes	1				not relevant
5.	yes	1				
6.	partly	0.5			Data collection year walkability not clear	
7.	no/notstat	0			dissemination area	
8.	yes	1				
9.	yes	1				
10.	yes	1				
11.	not relevar	0			ecological	
12.	yes	1				
13. Other:	yes	1				
14. Other:	not relevar	0				
		10.5	12	87.5		

Question	Answer	Points	Relevant q	Overall quality	Comments	Options
1.	yes	1				yes
2.	yes	1				partly
3.	no/notstat	0				no/notstated
4.	yes	1				not relevant
5.	yes	1				
6.	yes	1				
7.	no/notstat	0				
8.	yes	1				
9.	yes	1				
10.	yes	1				
11.	not relevar	0			ecological	
12.	yes	1				
13. Other:	partly	0.5			conflicts of interest not mentioned	
14. Other:	not relevar	0			publicly available	
		9.5	12	79.16667		

Question	Answer	Points	Relevant q	Overall quality	Comments	Options
1.	yes	1				yes
2.	yes	1				partly
3.	yes	1				no/notstated
4.	yes	1				not relevant
5.	yes	1				
6.	partly	0.5			No bus stops data available for 2000 so 1998 was used	
7.	no/notstat	0			Census tract level	
8.	no/notstat	0			Not described	
9.	yes	1				
10.	yes	1				
11.	not relevar	0			ecological	
12.	yes	1				
13. Other:	partly	0.5			Conflicts of interest not mentioned	
14. Other:	not relevar	0				
		9	12	75		

Edwards2011

Question	Answer	Points	Relevant q	Overall quality	Comments	Options
1.	yes	1				yes
2.	yes	1				partly
3.	yes	1				no/notstated
4.	yes	1				not relevant
5.	yes	1				
6.	no/notstat	0			2000 and 2008	
7.	no/notstat	0				
8.	no/notstat	0				
9.	yes	1				
10.	yes	1				
11.	not relevan	0			ecological study	
12.	yes	1				
13. Other:	no/notstat	0				
14. Other:	not relevan	0			publicly available data	
		8	12	66.66667		

Question	Answer	Points	Relevant q	Overall quality	Comments	Options
1.	yes	1				yes
2.	yes	1				partly
3.	yes	1				no/notstated
4.	yes	1				not relevant
5.	partly	0.5			Not clear which sports were included	
6.	no/notstat	0			Data collection year of locations not clear	
7.	no/notstat	0			Local government areas	
8.	no/notstat	0				
9.	partly	0.5				
10.	yes	1				
11.	not relevar	0			ecological data	
12.	yes	1				
13. Other:	yes	1				
14. Other:	not relevar	0			publicly available data	
		8	12	66.66667		

Question	Answer	Points	Relevant q	Overall quality	Comments	Options
1.	yes	1				yes
2.	yes	1				partly
3.	no/notstat	0			only income	no/notstated
4.	yes	1				not relevant
5.	yes	1				
6.	no/notstat	0			2000 and 2009-2010	
7.	no/notstat	0			census block level	
8.	no/notstat	0				
9.	yes	1				
10.	yes	1				
11.	not relevar	0			ecological data	
12.	yes	1				
13. Other:	partly	0.5			funding not mentioned	
14. Other:	not relevar	0			publicly available data	
		7.5	12	62.5		

Question	Answer	Points	Relevant q	Overall quality	Comments	Options
1.	yes	1				yes
2.	yes	1				partly
3.	yes	1				no/notstated
4.	yes	1				not relevant
5.	partly	0.5			Only description of % green space is: the percentage of land covered by open green space	
6.	no/notstat	0			Year of measurement environmental variables not stated	
7.	no/notstat	0			Neighbourhood level	
8.	partly	0.5			Only missing data related information described is that COVID-19 cases without neighbourhood identifiers and unconfirmed COVID-19 cases were excluded	
9.	partly	0.5			See arguments above	
10.	no/notstat	0			Not described what the numbers in the table entail, also no measure of spread reported	
11.	not relevar	0				
12.	yes	1				
13. Other:	yes	1				
14. Other:	not relevar	0				
		7.5	12	62.5		

Question	Answer	Points	Relevant q	Overall quality	Comments	Options
1.	yes	1				yes
2.	yes	1				partly
3.	yes	1				no/notstated
4.	yes	1				not relevant
5.	yes	1				
6.	yes	1				
7.	no/notstat	0			School level	
8.	no/notstat	0			Not descibed	
9.	yes	1				
10.	yes	1				
11.	not relevar	0			ecological	
12.	yes	1				
13. Other:	partly	0.5			Conflicts of interest not disclosed	
14. Other:	not relevar	0				
		9.5	12	79.16667		

Question	Answer	Points	Relevant q	Overall quality	Comments	Options
1.	yes	1				yes
2.	yes	1				partly
3.	yes	1				no/notstated
4.	yes	1				not relevant
5.	yes	1				
6.	no/notstat	0			2006 and 2009	
7.	no/notstat	0			Neighbourhood level	
8.	no/notstat	0				
9.	partly	0.5				
10.	partly	0.5				
11.	not relevar	0				
12.	yes	1				
13. Other:	partly	0.5			No conflicts of interest declared	
14. Other:	no/notstat	0			No ethical approval declared	
		7.5	13	57.69231		

Question	Answer	Points	Relevant q	Overall quality	Comments	Options
1.	yes	1				yes
2.	yes	1				partly
3.	no/notstat	0			Only municipality income	no/notstated
4.	no/notstat	0			Income not further described	not relevant
5.	yes	1				
6.	no/notstat	0			Year of income collection not described	
7.	no/notstat	0			Municipality level	
8.	no/notstat	0			Not described	
9.	partly	0.5				
10.	no/notstat	0			No clear table with summary results	
11.	not relevar	0				
12.	no/notstat	0			Limitations not discussed	
13. Other:	partly	0.5			Conflicts of interest not declared	
14. Other:	not relevar	0				
		4	12	33.33333		

Question	Answer	Points	Relevant q	Overall quality	Comments	Options
1.	yes	1				yes
2.	yes	1				partly
3.	yes	1				no/notstated
4.	yes	1				not relevant
5.	yes	1				
6.	no/notstat	0			2011 and 2019	
7.	no/notstat	0			LSOA level	
8.	no/notstat	0			Not discussed	
9.	partly	0.5				
10.	yes	1				
11.	not relevar	0			Based on administrative data, not survey data	
12.	yes	1				
13. Other:	yes	1				
14. Other:	not relevar	0				
		8.5	12	70.83333		

Question	Answer	Points	Relevant q	Overall quality	Comments	Options
1.	yes	1				yes
2.	yes	1				partly
3.	no/notstat	0			only education	no/notstated
4.	yes	1				not relevant
5.	yes	1				
6.	yes	1				
7.	no/notstat	0				
8.	partly	0.5			only described for missing greenspace data	
9.	yes	1				
10.	yes	1				
11.	not relevar	0			ecological data	
12.	no/notstat	0				
13. Other:	yes	1				
14. Other:	not relevar	0			publicly available data	
		8.5	12	70.83333		

Question	Answer	Points	Relevant q	Overall quality	Comments	Options
1.	yes	1				yes
2.	yes	1				partly
3.	yes	1				no/notstated
4.	yes	1				not relevant
5.	yes	1				
6.	no/notstat	0			2006-2010, 2015	
7.	no/notstat	0			Block group	
8.	no/notstat	0			not mentioned	
9.	partly	0.5				
10.	yes	1				
11.	not relevar	0			ecological data	
12.	partly	0.5			Only limitations of walk score shortly discussed	
13. Other:	partly	0.5			Only conflicts of interest mentioned	
14. Other:	not relevar	0				
		7.5	12	62.5		

Question	Answer	Points	Relevant q	Overall quality	Comments	Options
1.	yes	1				yes
2.	yes	1				partly
3.	yes	1				no/notstated
4.	yes	1				not relevant
5.	yes	1				
6.	partly	0.5			SEP 2011, walkability indicators between 2011 and 2015	
7.	no/notstat	0			LGA level	
8.	yes	1				
9.	yes	1				
10.	yes	1				
11.	yes	1				
12.	yes	1				
13. Other:	yes	1				
14. Other:	yes	1				
		12.5	14	89.28571		

Question	Answer	Points	Relevant q	Overall quality	Comments	Options
1.	yes	1				yes
2.	yes	1				partly
3.	yes	1				no/notstated
4.	yes	1				not relevant
5.	yes	1				
6.	no/notstat	0			2000 and 2007	
7.	no/notstat	0			census block group level	
8.	no/notstat	0			Not mentioned	
9.	partly	0.5				
10.	partly	0.5			No title or description for Table 1. Not clear what the N is in this table	
11.	not relevar	0			ecological data	
12.	no/notstat	0			Not mentioned	
13. Other:	partly	0.5			conflicts of interest not disclosed	
14. Other:	not relevar	0			publicly available data	
		6.5	12	54.16667		

Question	Answer	Points	Relevant q	Overall quality	Comments	Options
1.	yes	1				yes
2.	yes	1				partly
3.	no/notstat	0				no/notstated
4.	yes	1				not relevant
5.	yes	1				
6.	yes	1				
7.	no/notstat	0				
8.	no/notstat	0				
9.	yes	1				
10.	yes	1				
11.	not relevan	0				
12.	yes	1				
13. Other:	yes	1				
14. Other:	not relevan	0				
		9	12	75		

Question	Answer	Points	Relevant q	Overall quality	Comments	Options
1.	yes	1				yes
2.	yes	1				partly
3.	yes	1				no/notstated
4.	yes	1				not relevant
5.	yes	1				
6.	no/notstat	0			Year of PARA measurement not stated	
7.	no/notstat	0				
8.	yes	1				
9.	yes	1				
10.	yes	1				
11.	no/notstat	0				
12.	yes	1				
13. Other:	yes	1				
14. Other:	yes	1				
		11	14	78.57143		

Question	Answer	Points	Relevant q	Overall quality	Comments	Options
1.	yes	1				yes
2.	yes	1				partly
3.	yes	1				no/notstated
4.	yes	1				not relevant
5.	yes	1				
6.	no/notstat	0			2006 and 2011	
7.	no/notstat	0				
8.	no/notstat	0				
9.	yes	1				
10.	yes	1				
11.	not relevar	0			ecological data	
12.	no/notstat	0				
13. Other:	partly	0.5			conflicts of interest not mentioned	
14. Other:	not relevar	0			publicly available data	
		7.5	12	62.5		

Question	Answer	Points	Relevant q	Overall quality	Comments	Options
1.	yes	1				yes
2.	yes	1				partly
3.	yes	1				no/notstated
4.	yes	1				not relevant
5.	yes	1				
6.	no/notstat	0				
7.	no/notstat	0				
8.	no/notstat	0				
9.	partly	0.5				
10.	yes	1				
11.	not relevar	0			ecological data	
12.	yes	1				
13. Other:	partly	0.5			conflicts of interest not mentioned	
14. Other:	not relevar	0				
		8	12	66.66667		

Question	Answer	Points	Relevant q	Overall quality	Comments	Options
1.	yes	1				yes
2.	yes	1				partly
3.	yes	1				no/notstated
4.	yes	1				not relevant
5.	yes	1				
6.	partly	0.5			Data on blue spaces not	
7.	yes	1				
8.	yes	1				
9.	yes	1				
10.	partly	0.5			No descriptives/summary statistics provided	
11.	no/notstat	0				
12.	yes	1				
13. Other:	yes	1				
14. Other:	yes	1				
		12	14	85.71429		

Question	Answer	Points	Relevant q	Overall quality	Comments	Options
1.	yes	1				yes
2.	yes	1				partly
3.	yes	1				no/notstated
4.	yes	1				not relevant
5.	yes	1				
6.	yes	1				
7.	no/notstat	0				
8.	no/notstat	0				
9.	yes	1				
10.	yes	1				
11.	not relevar	0			ecological data	
12.	yes	1				
13. Other:	partly	0.5			funding not mentioned	
14. Other:	not relevar	0			publicly available	
		9.5	12	79.16667		

Garrison2019

Question	Answer	Points	Relevant q	Overall quality	Comments	Options
1.	yes	1				yes
2.	yes	1				partly
3.	no/notstat	0				no/notstated
4.	yes	1				not relevant
5.	yes	1				
6.	yes	1				
7.	no/notstat	0				
8.	no/notstat	0				
9.	yes	1				
10.	partly	0.5			No descriptives/summary stats provided	
11.	not relevar	0			ecological data	
12.	no/notstat	0				
13. Other:	yes	1				
14. Other:	not relevar	0			publicly available data	
		7.5	12	62.5		

Question	Answer	Points	Relevant q	Overall quality	Comments	Options
1.	yes	1				yes
2.	yes	1				partly
3.	yes	1				no/notstated
4.	no/notstat	0			No reference to index-developers	not relevant
5.	yes	1				
6.	no/notstat	0			Data collection environmental variables not clear	
7.	yes	1				
8.	no/notstat	0				
9.	partly	0.5				
10.	yes	1				
11.	yes	1				
12.	yes	1				
13. Other:	partly	0.5			conflicts of interest not disclosed	
14. Other:	yes	1				
		10	14	71.42857		

Question	Answer	Points	Relevant q	Overall quality	Comments	Options
1.	yes	1				yes
2.	yes	1				partly
3.	yes	1				no/notstated
4.	yes	1				not relevant
5.	yes	1				
6.	no/notstat	0			2001 and 2005	
7.	no/notstat	0				
8.	no/notstat	0				
9.	partly	0.5				
10.	no/notstat	0			No table with descriptives/summary statistics provided	
11.	not relevar	0			ecological data	
12.	no/notstat	0				
13. Other:	no/notstat	0				
14. Other:	not relevar	0			publicly available data	
		5.5	12	45.83333		

Question	Answer	Points	Relevant q	Overall quality	Comments	Options
1.	yes	1				yes
2.	yes	1				partly
3.	yes	1				no/notstated
4.	yes	1				not relevant
5.	yes	1				
6.	partly	0.5			NDVI yes, green land cover no	
7.	yes	1				
8.	yes	1				
9.	yes	1				
10.	yes	1				
11.	no/notstat	0				
12.	yes	1				
13. Other:	partly	0.5			conflicts of interest not disclosed	
14. Other:	no/notstat	0				
		11	14	78.57143		

Gullón2017

Question	Answer	Points	Relevant q	Overall quality	Comments	Options
1.	yes	1				yes
2.	yes	1				partly
3.	yes	1				no/notstated
4.	yes	1				not relevant
5.	yes	1				
6.	partly	0.5			1 component of walkability index measured in 2011	
7.						
8.	no/notstat	0				
9.	yes	1				
10.	partly	0.5			No descriptives/summary statistics provided	
11.	not relevar	0			ecological data	
12.	yes	1				
13. Other:	yes	1				
14. Other:	not relevar	0				
		9	11	81.81818		

Gullón2023

Question	Answer	Points	Relevant q	Overall quality	Comments	Options
1.	yes	1				yes
2.	yes	1				partly
3.	yes	1				no/notstated
4.	yes	1				not relevant
5.	partly	0.5			No details on walkability described	
6.	partly	0.5			Data collection year walkability not clear	
7.	no/notstat	0				
8.	yes	1				
9.	yes	1				
10.	yes	1				
11.	no/notstat	0				
12.	yes	1				
13. Other:	yes	1				
14. Other:	yes	1				
		11	14	78.57143		

Question	Answer	Points	Relevant q	Overall quality	Comments	Options
1.	yes	1				yes
2.	yes	1				partly
3.	no/notstat	0				no/notstated
4.	yes	1				not relevant
5.	yes	1				
6.	no/notstat	0				
7.	no/notstat	0				
8.	yes	1				
9.	yes	1				
10.	yes	1				
11.	no/notstat	0				
12.	no/notstat	0				
13. Other:	partly	0.5			conflicts of interest not disclosed	
14. Other:	yes	1				
		8.5	14	60.71429		

Question	Answer	Points	Relevant q	Overall quality	Comments	Options
1.	yes	1				yes
2.	yes	1				partly
3.	yes	1				no/notstated
4.	yes	1				not relevant
5.	yes	1				
6.	no/notstat	0			Data collection years not clear	
7.	no/notstat	0			neighbourhood level	
8.	no/notstat	0				
9.	yes	1				
10.	no/notstat	0			No table with descriptives/summary statistics provided	
11.	not relevar	0			ecological data	
12.	yes	1				
13. Other:	yes	1				
14. Other:	not relevar	0			publicly available data	
		8	12	66.66667		

Question	Answer	Points	Relevant q	Overall quality	Comments	Options
1.	yes	1				yes
2.	yes	1				partly
3.	no/notstat	0				no/notstated
4.	no/notstat	0			no detailed description of public h	not relevant
5.	no/notstat	0			no detailed description of exposure variables	
6.	yes	1				
7.	yes	1				
8.	no/notstat	0				
9.	no/notstat	0			Methods section very short and not detailed. Not clear which statistical test was used for which result	
10.	yes	1				
11.	not relevar	0				
12.	yes	1				
13. Other:	no/notstat	0				
14. Other:	not relevar	0			no participants involved	
		6	12	50		

Question	Answer	Points	Relevant q	Overall quality	Comments	Options
1.	yes	1				yes
2.	yes	1				partly
3.	yes	1				no/notstated
4.	yes	1				not relevant
5.	yes	1				
6.	partly	0.5			Data collection years of environmental variables not very clear	
7.	no/notstat	0				
8.	no/notstat	0				
9.	yes	1				
10.	yes	1				
11.	not relevar	0			ecological data	
12.	yes	1				
13. Other:	partly	0.5			conflicts of interest not disclosed	
14. Other:	not relevar	0			publicly available data	
		9	12	75		

Question	Answer	Points	Relevant q	Overall quality	Comments	Options
1.	yes	1				yes
2.	yes	1				partly
3.	no/notstat	0				no/notstated
4.	yes	1				not relevant
5.	no/notstat	0				
6.	no/notstat	0			Data collection years environmental variables not clear	
7.	no/notstat	0				
8.	yes	1				
9.	yes	1				
10.	yes	1				
11.	not relevar	0			ecological data	
12.	yes	1				
13. Other:	yes	1				
14. Other:	not relevar	0			publicly available data and no participants involved	
		8	12	66.66667		

Question	Answer	Points	Relevant q	Overall quality	Comments	Options
1.	yes	1				yes
2.	yes	1				partly
3.	yes	1				no/notstated
4.	yes	1				not relevant
5.	yes	1				
6.	partly	0.5			SEP 2010-2012, Walk Score 2012	
7.	yes	1				
8.	yes	1				
9.	yes	1				
10.	yes	1				
11.	no/notstat	0				
12.	yes	1				
13. Other:	partly	0.5			Conflicts of interest not disclosed	
14. Other:	yes	1				
		12	14	85.71429		

Hoffiman2017

Question	Answer	Points	Relevant q	Overall quality	Comments	Options
1.	yes	1				yes
2.	yes	1				partly
3.	yes	1				no/notstated
4.	yes	1				not relevant
5.	yes	1				
6.	no/notstat	0			data collection SEP not clear	
7.	no/notstat	0			census tract	
8.	no/notstat	0				
9.	yes	1				
10.	yes	1				
11.	not relevan	0			ecological data	
12.	yes	1				
13. Other:	yes	1				
14. Other:	not relevan	0			no participants involved	
		9	12	75		

Question	Answer	Points	Relevant q	Overall quality	Comments	Options
1.	yes	1				yes
2.	yes	1				partly
3.	no/notstat	0				no/notstated
4.	yes	1				not relevant
5.	yes	1				
6.	partly	0.5			Data collection years of SEP variables for regression model with 2016 cycling variables not clear	
7.	no/notstat	0				
8.	no/notstat	0				
9.	yes	1				
10.	yes	1				
11.	not relevar	0			ecological data	
12.	no/notstat	0				
13. Other:	no/notstat	0				
14. Other:	not relevar	0			publicly available data and no participants involved	
		6.5	12	54.16667		

Hughey2016

Question	Answer	Points	Relevant q	Overall quality	Comments	Options
1.	yes	1				yes
2.	yes	1				partly
3.	yes	1				no/notstated
4.	yes	1				not relevant
5.	yes	1				
6.	no/notstat	0			years of data collection park availability not clear	
7.	no/notstat	0			block group level	
8.	no/notstat	0				
9.	yes	1				
10.	yes	1				
11.	not relevan	0			ecological data	
12.	yes	1				
13. Other:	partly	0.5			conflicts of interest not disclosed	
14. Other:	not relevan	0			publicly available data and no participants involved	
		8.5	12	70.83333		

Question	Answer	Points	Relevant q	Overall quality	Comments	Options
1.	yes	1				yes
2.	yes	1				partly
3.	no/notstat	0				no/notstated
4.	yes	1				not relevant
5.	yes	1				
6.	yes	1				
7.	no/notstat	0				
8.	no/notstat	0				
9.	yes	1				
10.	yes	1				
11.	not relevar	0			ecological study	
12.	no/notstat	0				
13. Other:	yes	1				
14. Other:	not relevar	0			no participants involved	
		8	12	66.66667		

Question	Answer	Points	Relevant q	Overall quality	Comments	Options
1.	yes	1				yes
2.	yes	1				partly
3.	yes	1				no/notstated
4.	yes	1				not relevant
5.	yes	1				
6.	yes	1				
7.	no/notstat	0				
8.	yes	1				
9.	yes	1				
10.	yes	1				
11.	no/notstat	0				
12.	yes	1				
13. Other:	yes	1				
14. Other:	not relevar	0			Quote: ´because existing data sources were used, no written consent was required for participation in the study´	
		11	13	84.61538		

Question	Answer	Points	Relevant q	Overall quality	Comments	Options
1.	yes	1				yes
2.	yes	1				partly
3.	yes	1				no/notstated
4.	yes	1				not relevant
5.	yes	1				
6.	partly	0.5			SEP partly 2000-2008, NDVI 2000	
7.	yes	1				
8.	yes	1				
9.	yes	1				
10.	partly	0.5			Not clear how census tract data was aggregated to measure NDVI in descriptive table	
11.	yes	1				
12.	yes	1				
13. Other:	yes	1				
14. Other:	yes	1				
		13	14	92.85714		

Question	Answer	Points	Relevant q	Overall quality	Comments	Options
1.	partly	0.5			Research aim not stated very clear	yes
2.	yes	1				partly
3.	no/notstat	0			Income	no/notstated
4.	yes	1				not relevant
5.	yes	1				
6.	yes	1				
7.	yes	1				
8.	no/notstat	0				
9.	yes	1				
10.	yes	1				
11.	no/notstat	0				
12.	no/notstat	0				
13. Other:	partly	0.5			Conflicts of interest not disclosed	
14. Other:	no/notstat	0				
		8	14	57.14286		

Question	Answer	Points	Relevant q	Overall quality	Comments	Options
1.	yes	1				yes
2.	yes	1				partly
3.	no/notstat	0			income	no/notstated
4.	no/notstat	0			Only descibed: "telephone survey	not relevant
5.	yes	1				
6.	yes	1				
7.	yes	1				
8.	no/notstat	0				
9.	no/notstat	0			too little information on income measurement provided	
10.	yes	1				
11.	no/notstat	0				
12.	no/notstat	0				
13. Other:	no/notstat	0				
14. Other:	no/notstat	0				
		6	14	42.85714		

Question	Answer	Points	Relevant q	Overall quality	Comments	Options
1.	yes	1				yes
2.	yes	1				partly
3.	yes	1				no/notstated
4.	yes	1				not relevant
5.	yes	1				
6.	yes	1				
7.	no/notstat	0			Block group	
8.	no/notstat	0				
9.	yes	1				
10.	no/notstat	0			No descriptives/summary statistics provided	
11.	not relevar	0			ecological data	
12.	yes	1				
13. Other:	no/notstat	0				
14. Other:	not relevar	0			publicly available data, no participants involved	
		8	12	66.66667		

Question	Answer	Points	Relevant q	Overall quality	Comments	Options
1.	yes	1				yes
2.	yes	1				partly
3.	yes	1				no/notstated
4.	yes	1				not relevant
5.	yes	1				
6.	no/notstat	0			2010 and 2005	
7.	no/notstat	0			planning unit level	
8.	no/notstat	0				
9.	yes	1				
10.	no/notstat	0			No descriptives/summary statistics provided	
11.	not relevar	0			ecological data	
12.	no/notstat	0				
13. Other:	no/notstat	0				
14. Other:	not relevar	0			publicly available data, no participants involved	
		6	12	50		

Question	Answer	Points	Relevant q	Overall quality	Comments	Options
1.	yes	1				yes
2.	yes	1				partly
3.	no/notstat	0				no/notstated
4.	yes	1				not relevant
5.	yes	1				
6.	yes	1				
7.	no/notstat	0				
8.	no/notstat	0				
9.	no/notstat	0			Not clear from methods section that univariate analyses were carried out	
10.	no/notstat	0			No descriptives/summary stats provided	
11.	not relevar	0			ecological data	
12.	yes	1				
13. Other:	yes	1				
14. Other:	not relevar	0			publicly available data, no participants involved	
		7	12	58.33333		

Question	Answer	Points	Relevant q	Overall quality	Comments	Options
1.	yes	1				yes
2.	yes	1				partly
3.	yes	1				no/notstated
4.	yes	1				not relevant
5.	yes	1				
6.	partly	0.5			SEP 2017, parks 2015, PA facilities 2018	
7.	no/notstat	0				
8.	no/notstat	0				
9.	yes	1				
10.	yes	1				
11.	not relevar	0			ecological data	
12.	yes	1				
13. Other:	partly	0.5			funding sources not disclosed	
14. Other:	not relevar	0			publicly available data, no participants involved	
		9	12	75		

Question	Answer	Points	Relevant q	Overall quality	Comments	Options
1.	yes	1				yes
2.	yes	1				partly
3.	yes	1				no/notstated
4.	yes	1				not relevant
5.	yes	1				
6.	no/notstat	0			Not clear whether Townsend score of 2001 or 2002 is used. Bus stop data from 2011 used	
7.	no/notstat	0				
8.	no/notstat	0				
9.	yes	1				
10.	no/notstat	0			Confusing from Table 1 which deprivation quintile is low/high	
11.	not relevar	0			ecologial data	
12.	no/notstat	0				
13. Other:	partly	0.5			conflicts of interest not disclosed	
14. Other:	not relevar	0			no participants involved	
		6.5	12	54.16667		

Question	Answer	Points	Relevant q	Overall quality	Comments	Options
1.	yes	1				yes
2.	yes	1				partly
3.	yes	1				no/notstated
4.	yes	1				not relevant
5.	yes	1				
6.	no/notstat	0			2000, 2003-2007 and 2018	
7.	yes	1				
8.	yes	1				
9.	yes	1				
10.	yes	1				
11.	no/notstat	0				
12.	yes	1				
13. Other:	yes	1				
14. Other:	yes	1				
		12	14	85.71429		

Question	Answer	Points	Relevant q	Overall quality	Comments	Options
1.	yes	1				yes
2.	yes	1				partly
3.	no/notstat	0				no/notstated
4.	yes	1				not relevant
5.	no/notstat	0			Walkability measure not described	
6.	no/notstat	0			Data collection years walkability not clear	
7.	yes	1				
8.	yes	1				
9.	no/notstat	0			Walkability, which is the main exposure, not described	
10.	yes	1				
11.	no/notstat	0				
12.	yes	1				
13. Other:	partly	0.5			conflicts of interest not disclosed	
14. Other:	yes	1				
		8.5	14	60.71429		

Question	Answer	Points	Relevant q	Overall quality	Comments	Options
1.	yes	1				yes
2.	yes	1				partly
3.	no/notstat	0				no/notstated
4.	yes	1				not relevant
5.	partly	0.5			Only reported that categorical years of education was included in the model	
6.	partly	0.5			2007-2009 and 2007	
7.	yes	1				
8.	no/notstat	0				
9.	yes	1				
10.	yes	1				
11.	no/notstat	0				
12.	yes	1				
13. Other:	partly	0.5			funding sources not disclosed	
14. Other:	yes	1				
		9.5	14	67.85714		

Question	Answer	Points	Relevant q	Overall quality	Comments	Options
1.	yes	1				yes
2.	yes	1				partly
3.	yes	1				no/notstated
4.	yes	1				not relevant
5.	no/notstat	0			Walkability measure retrieved with exploratory factor analysis, but no further details provided	
6.	no/notstat	0			Data collection neighbourhood deprivation not clear	
7.	yes	1				
8.	yes	1				
9.	yes	1				
10.	yes	1				
11.	no/notstat	0				
12.	yes	1				
13. Other:	yes	1				
14. Other:	yes	1				
		11	14	78.57143		

Question	Answer	Points	Relevant q	Overall quality	Comments	Options
1.	yes	1				yes
2.	yes	1				partly
3.	yes	1				no/notstated
4.	yes	1				not relevant
5.	no/notstat	0			No details on development of walkability index provided	
6.	no/notstat	0			Data collection years walkability components not clear	
7.	yes	1				
8.	no/notstat	0				
9.	no/notstat	0			Walkability not described clearly, which is one of the main exposure variables	
10.	yes	1				
11.	yes	1				
12.	yes	1				
13. Other:	yes	1				
14. Other:	yes	1				
		10	14	71.42857		

Question	Answer	Points	Relevant q	Overall quality	Comments	Options
1.	yes	1				yes
2.	yes	1				partly
3.	no/notstat	0				no/notstated
4.	yes	1				not relevant
5.	no/notstat	0			No details on development of walkability index provided	
6.	no/notstat	0			Data collection years walkability components not clear	
7.	no/notstat	0			Neighbourhood level	
8.	no/notstat	0				
9.	no/notstat	0			Walkability not described clearly, which is one of the main exposure variables	
10.	yes	1				
11.	no/notstat	0				
12.	yes	1				
13. Other:	yes	1				
14. Other:	yes	1				
		7	14	50		

Question	Answer	Points	Relevant q	Overall quality	Comments	Options
1.	yes	1				yes
2.	yes	1				partly
3.	no/notstat	0				no/notstated
4.	no/notstat	0			Only description for income is "Deprivation was derived from rank of income deprivation, a domain of tl	not relevant
5.	yes	1				
6.	no/notstat	0			2004 and 2009	
7.	no/notstat	0			LSOA	
8.	no/notstat	0				
9.	no/notstat	0			Too little description on one of the main determinants (income), also statistical analysis not described in detail	
10.	no/notstat	0			No descriptives/summary stats provided	
11.	not relevar	0			ecological data	
12.	yes	1				
13. Other:	yes	1				
14. Other:	not relevar	0			publicly available data, no participants involved	
		5	12	41.66667		

Question	Answer	Points	Relevant q	Overall quality	Comments	Options
1.	yes	1				yes
2.	yes	1				partly
3.	yes	1				no/notstated
4.	yes	1				not relevant
5.	partly	0.5			Reference provided for green space data, but not for public transport data	
6.	no/notstat	0			SEP in 2017, greens paces in 2013, public transport data collection years not clear	
7.	no/notstat	0				
8.	no/notstat	0				
9.	partly	0.5			Public transport data not described detailed enough to be able to repeat analyses	
10.	yes	1				
11.	yes	1				
12.	yes	1				
13. Other:	partly	0.5			conflicts of interest not disclosed	
14. Other:	no/notstat	0				
		8.5	14	60.71429		

Question	Answer	Points	Relevant q	Overall quality	Comments	Options
1.	yes	1				yes
2.	yes	1				partly
3.	yes	1				no/notstated
4.	yes	1				not relevant
5.	yes	1				
6.	yes	1				
7.	no/notstat	0			SA1	
8.	no/notstat	0				
9.	yes	1				
10.	yes	1				
11.	not relevan	0			ecological data	
12.	yes	1				
13. Other:	partly	0.5			conflicts of interest not disclosed	
14. Other:	not relevan	0			publicly available data, no participants involved	
		9.5	12	79.16667		

Question	Answer	Points	Relevant q	Overall quality	Comments	Options
1.	yes	1				yes
2.	yes	1				partly
3.	no/notstat	0				no/notstated
4.	yes	1				not relevant
5.	yes	1				
6.	yes	1				
7.	yes	1				
8.	partly	0.5			Only stated: Using a geographic information system (GIS; ArcGIS 10.2.2), 94.6% of the youths' home addresses were successfully geocoded at the point address level, which was used as the final sample (n = 13,469). No missing data handling of other variables described.	
9.	partly	0.5			Data collection method sociodemographic variables not reported	
10.	yes	1				
11.	no/notstat	0			Not clear if sociodemographics were collected via questionnaires, so also not clear whether or not non-response was relevant	
12.	yes	1				
13. Other:	partly	0.5			Funding sources not disclosed. Conflicts of interest statement only about financial conflicts: No competing financial interests exist.	
14. Other:	no/notstat	0				
		9.5	14	67.85714		

Question	Answer	Points	Relevant q	Overall quality	Comments	Options
1.	partly	0.5			Only in abstract	yes
2.	yes	1				partly
3.	no/notstat	0				no/notstated
4.	yes	1				not relevant
5.	yes	1				
6.	no/notstat	0			SEP 2001, NDVI 1991-1995	
7.	yes	1				
8.	partly	0.5			Missings reported in descriptive table, but no information on missing data handling	
9.	yes	1				
10.	yes	1				
11.	yes	1				
12.	partly	0.5			Only this sentence stated: 'Our use of self-reported measures of physical activity inevitably introduces some degree of exposure misclassification.'	
13. Other:	no/notstat	0				
14. Other:	yes	1				
		9.5	14	67.85714		

Question	Answer	Points	Relevant q	Overall quality	Comments	Options
1.	yes	1				yes
2.	yes	1				partly
3.	no/notstat	0				no/notstated
4.	no/notstat	0			Income operationalisation not described in methods	not relevant
5.	no/notstat	0			Methods to measure median number of parks and park acreage nog described	
6.	no/notstat	0			Data collection years not clear	
7.	no/notstat	0			block group level	
8.	no/notstat	0				
9.	no/notstat	0			Methods very short and no details provided	
10.	yes	1				
11.	not relevar	0			ecological data	
12.	no/notstat	0				
13. Other:	no/notstat	0				
14. Other:	not relevar	0			no participants involved	
		3	12	25		

Question	Answer	Points	Relevant q	Overall quality	Comments	Options
1.	yes	1				yes
2.	yes	1				partly
3.	yes	1				no/notstated
4.	partly	0.5			Index described accurately, but separate income variable not	not relevant
5.	yes	1				
6.	no/notstat	0			2012 and 2018	
7.	no/notstat	0			Block/commune	
8.	no/notstat	0				
9.	no/notstat	0			Variables and statistical methods not described detailed enough to be able to be repeated. Not clear how bike lanes within certain distance from people's homes were measured.	
10.	no/notstat	0			No descriptives/summary stats on socioeconomic data provided	
11.	not relevar	0			ecological data	
12.	no/notstat	0				
13. Other:	partly	0.5			conflicts of interest not disclosed	
14. Other:	not relevar	0			no participants involved	
		5	12	41.66667		

Question	Answer	Points	Relevant q	Overall quality	Comments	Options
1.	yes	1				yes
2.	yes	1				partly
3.	yes	1				no/notstated
4.	yes	1				not relevant
5.	yes	1				
6.	yes	1				
7.	yes	1				
8.	yes	1				
9.	yes	1				
10.	yes	1				
11.	no/notstat	0				
12.	yes	1				
13. Other:	yes	1				
14. Other:	yes	1				
		13	14	92.85714		

Question	Answer	Points	Relevant q	Overall quality	Comments	Options
1.	yes	1				yes
2.	yes	1				partly
3.	yes	1				no/notstated
4.	partly	0.5			Referred to ACS with: 'The social vulnerability indicators were retrieved from the most updated 5-year	not relevant
5.	yes	1				
6.	no/notstat	0			2014-2018 and 2020	
7.	no/notstat	0			parcel level and zip code level	
8.	no/notstat	0				
9.	no/notstat	0			Too little information on operationalisation of socioeconomics to be able to repeat methods	
10.	partly	0.5			No descriptives on socioeconomics provided	
11.	no/notstat	0				
12.	no/notstat	0				
13. Other:	yes	1				
14. Other:	no/notstat	0				
		6	14	42.85714		

Question	Answer	Points	Relevant q	Overall quality	Comments	Options
1.	yes	1				yes
2.	yes	1				partly
3.	yes	1				no/notstated
4.	yes	1				not relevant
5.	yes	1				
6.	no/notstat	0			2011 and 2021	
7.	yes	1				
8.	yes	1				
9.	yes	1				
10.	yes	1				
11.	no/notstat	0				
12.	yes	1				
13. Other:	yes	1				
14. Other:	yes	1				
		12	14	85.71429		

Question	Answer	Points	Relevant q	Overall quality	Comments	Options
1.	yes	1				yes
2.	yes	1				partly
3.	yes	1				no/notstated
4.	yes	1				not relevant
5.	yes	1				
6.	no/notstat	0			Not clear when population density was collected	
7.	yes	1				
8.	no/notstat	0				
9.	yes	1				
10.	yes	1				
11.	yes	1				
12.	yes	1				
13. Other:	yes	1				
14. Other:	yes	1				
		12	14	85.71429		

Question	Answer	Points	Relevant q	Overall quality	Comments	Options
1.	yes	1				yes
2.	yes	1				partly
3.	no/notstat	0				no/notstated
4.	yes	1				not relevant
5.	yes	1				
6.	no/notstat	0			Data collection years active transportation infrastructure not clear	
7.	no/notstat	0			Census tract	
8.	no/notstat	0				
9.	yes	1				
10.	yes	1				
11.	not relevan	0			ecological data	
12.	yes	1				
13. Other:	no/notstat	0				
14. Other:	not relevan	0			publicly available data, no participants involved	
		7	12	58.33333		

Question	Answer	Points	Relevant q	Overall quality	Comments	Options
1.	partly	0.5			`While the 2007 Urban Forest Action Plan has no mention of or strategies to address urban green equity	yes
2.	yes	1				partly
3.	yes	1				no/notstated
4.	no/notstat	0			% bachelor, master and professional degree not described in methods section	not relevant
5.	yes	1				
6.	partly	0.5			SEP 2009-2013, NDVI 2010, park data not stated	
7.	no/notstat	0			block group	
8.	no/notstat	0				
9.	partly	0.5			More desription of education operationalisation necessary to be able to repeat analyses	
10.	no/notstat	0			No summary stats/descriptives provided	
11.	not relevar	0			ecological data	
12.	no/notstat	0				
13. Other:	partly	0.5			funding not disclosed	
14. Other:	not relevar	0			publicly available data, no participants involved	
		5	12	41.66667		

Question	Answer	Points	Relevant q	Overall quality	Comments	Options
1.	yes	1				yes
2.	yes	1				partly
3.	no/notstat	0				no/notstated
4.	yes	1				not relevant
5.	yes	1				
6.	partly	0.5			SEP 2013, parks 2010, NDVI 2013-2015	
7.	no/notstat	0			CT and Block group	
8.	no/notstat	0				
9.	yes	1				
10.	yes	1				
11.	not relevar	0			ecological data	
12.	yes	1				
13. Other:	partly	0.5			conflicts of interest not disclosed	
14. Other:	not relevar	0			publicly available data	
		8	12	66.66667		

Question	Answer	Points	Relevant q	Overall quality	Comments	Options
1.	yes	1				yes
2.	yes	1				partly
3.	no/notstat	0				no/notstated
4.	yes	1				not relevant
5.	yes	1				
6.	partly	0.5			metro and underground stop locations collected from 2010. All other variables measured in 2020 or 2021	
7.	partly	0.5			Residential location and residential dwelling	
8.	no/notstat	0				
9.	yes	1				
10.	yes	1				
11.	not relevar	0			ecological data	
12.	yes	1				
13. Other:	partly	0.5			conflicts of interest not disclosed	
14. Other:	not relevar	0			publicly available data, no participants involved	
		8.5	12	70.83333		

Question	Answer	Points	Relevant q	Overall quality	Comments	Options
1.	yes	1				yes
2.	yes	1				partly
3.	no/notstat	0				no/notstated
4.	yes	1				not relevant
5.	yes	1				
6.	no/notstat	0			Data collection year vegetation index not clear	
7.	no/notstat	0			census tract	
8.	no/notstat	0				
9.	yes	1				
10.	yes	1				
11.	not relevar	0			ecological data	
12.	no/notstat	0				
13. Other:	partly	0.5			conflicts of interest not disclosed	
14. Other:	not relevar	0			publilcy available data and no participants involved	
		6.5	12	54.16667		

Question	Answer	Points	Relevant	Overall quality	Comments	Options
1.	yes	1				yes
2.	yes	1				partly
3.	yes	1				no/notstated
4.	yes	1				not relevant
5.	yes	1				
6.	partly	0.5			GVI 2009-2019, NDVI 2020, SEP 2016	
7.	no/notsta	0				
8.	no/notsta	0				
9.	yes	1				
10.	yes	1				
11.	not releva	0			ecological data	
12.	partly	0.5			Only: "This research assumes that greenspace exposure is determined based on its availability, which is a key requirement for greenspace use. However, other determinants contribute to greenspace use,79 for instance, accessibility to greenspace. Another factor that impacts greenspace use is perceived safety and security. High crime rates and antisocial behavior discourage residents from utilizing such spaces. For this large-scale study, data to provide insights for such factors are not readily available or easy to gather."	
13.	yes	1				
14.	not releva	0			publicly available data, no participants involved	
		9	12	75		

Question	Answer	Points	Relevant q	Overall quality	Comments	Options
1.	yes	1				yes
2.	yes	1				partly
3.	yes	1				no/notstated
4.	yes	1				not relevant
5.	yes	1				
6.	no/notstat	0			Data collection years greenspaces not clear	
7.	no/notstat	0			census block	
8.	no/notstat	0				
9.	yes	1				
10.	yes	1				
11.	not relevar	0			ecological data	
12.	yes	1				
13. Other:	yes	1				
14. Other:	not relevar	0			publicly available data, no participants involved	
		9	12	75		

Question	Answer	Points	Relevant q	Overall quality	Comments	Options
1.	yes	1				yes
2.	yes	1				partly
3.	yes	1				no/notstated
4.	yes	1				not relevant
5.	yes	1				
6.	no/notstat	0			Data collection years exposure variables not clear	
7.	yes	1				
8.	yes	1				
9.	no/notstat	0			Methods section on statistical analysis very short and general	
10.	yes	1				
11.	yes	1				
12.	yes	1				
13. Other:	no/notstat	0				
14. Other:	no/notstat	0				
		10	14	71.42857		

Question	Answer	Points	Relevant q	Overall quality	Comments	Options
1.	yes	1				yes
2.	yes	1				partly
3.	yes	1				no/notstated
4.	yes	1				not relevant
5.	yes	1				
6.	no/notstat	0			data collection years exposure variable not clear	
7.	no/notstat	0			census block groups	
8.	no/notstat	0				
9.	yes	1				
10.	yes	1				
11.	not relevar	0			ecological data	
12.	yes	1				
13. Other:	partly	0.5			funding sources not disclosed	
14. Other:	not relevar	0			publicly available data, no participants involved	
		8.5	12	70.83333		

Question	Answer	Points	Relevant q	Overall quality	Comments	Options
1.	yes	1				yes
2.	yes	1				partly
3.	no/notstat	0				no/notstated
4.	yes	1				not relevant
5.	yes	1				
6.	no/notstat	0			2000 and 2003,2004	
7.	no/notstat	0			census tract	
8.	no/notstat	0				
9.	yes	1				
10.	yes	1				
11.	not relevar	0			ecological data	
12.	yes	1				
13. Other:	yes	1				
14. Other:	not relevar	0			publicly available data, no participants involved	
		8	12	66.66667		

Question	Answer	Points	Relevant q	Overall quality	Comments	Options
1.	yes	1				yes
2.	yes	1				partly
3.	yes	1				no/notstated
4.	yes	1				not relevant
5.	yes	1				
6.	yes	1				
7.	yes	1				
8.	yes	1				
9.	yes	1				
10.	yes	1				
11.	no/notstat	0				
12.	yes	1				
13. Other:	yes	1				
14. Other:	yes	1				
		13	14	92.85714		

Question	Answer	Points	Relevant q	Overall quality	Comments	Options
1.	yes	1				yes
2.	yes	1				partly
3.	no/notstat	0				no/notstated
4.	no/notstat	0			Mentioned that income was retrie	not relevant
5.	no/notstat	0			The terms 'public parks with community playgrounds', 'community parks' and 'community playgrounds' were used interchangeably without description/definition	
6.	no/notstat	0			Data collection years not clear	
7.	no/notstat	0			Census tract	
8.	no/notstat	0				
9.	no/notstat	0			Methods section very short and general, not possible to repeat	
10.	no/notstat	0			No descriptives/summary stats provided	
11.	not relevar	0			Ecological data	
12.	yes	1				
13. Other:	yes	1				
14. Other:	not relevar	0			publicly available data, no participants involved	
		4	12	33.33333		

Note: This

Question	Answer	Points	Relevant q	Overall quality	Comments	Options
1.	yes	1				yes
2.	yes	1				partly
3.	no/notstat	0				no/notstated
4.	yes	1				not relevant
5.	yes	1				
6.	no/notstat	0			SEP 2004, 2005, Greenspaces 2000-2001	
7.	no/notstat	0				
8.	no/notstat	0				
9.	yes	1			Statistical methods used to retrieve results of figure 1 not clearly stated	
10.	partly	0.5			No descriptives/summary stats provided	
11.	not relevar	0			ecological data	
12.	yes	1				
13. Other:	no/notstat	0				
14. Other:	not relevar	0			No participants involved	
		6.5	12	54.16667		

Question	Answer	Points	Relevant q	Overall quality	Comments	Options
1.	yes	1				yes
2.	yes	1				partly
3.	yes	1				no/notstated
4.	yes	1				not relevant
5.	yes	1				
6.	partly	0.5			SEP 2009-2013, NDVI 2011, other environmental exposures not clear when collected	
7.	no/notstat	0			census block	
8.	no/notstat	0				
9.	yes	1				
10.	yes	1				
11.	not relevar	0			ecological data	
12.	no/notstat	0				
13. Other:	no/notstat	0				
14. Other:	not relevar	0			publicly available data, no participants involved	
		7.5	12	62.5		

Question	Answer	Points	Relevant q	Overall quality	Comments	Options
1.	yes	1				yes
2.	yes	1				partly
3.	no/notstat	0				no/notstated
4.	yes	1				not relevant
5.	yes	1				
6.	no/notstat	0			data collection years SEP not clear	
7.	no/notstat	0				
8.	no/notstat	0				
9.	yes	1				
10.	yes	1				
11.	not relevar	0			ecological data	
12.	no/notstat	0				
13. Other:	partly	0.5			conflicts of interest not disclosed	
14. Other:	not relevar	0			publicly available data, no participants involved	
		6.5	12	54.16667		

Question	Answer	Points	Relevant q	Overall quality	Comments	Options
1.	yes	1				yes
2.	yes	1				partly
3.	yes	1				no/notstated
4.	yes	1				not relevant
5.	yes	1				
6.	no/notstat	0			2016 and 2012	
7.	yes	1				
8.	partly	0.5			missing data handling NDVI described but not missing data handling SEP	
9.	yes	1				
10.	yes	1				
11.	yes	1				
12.	yes	1				
13. Other:	no/notstat	0				
14. Other:	not relevan	0			publicly available data	
		10.5	13	80.76923		

Question	Answer	Points	Relevant q	Overall quality	Comments	Options
1.	yes	1				yes
2.	yes	1				partly
3.	yes	1				no/notstated
4.	partly	0.5			Scale and interpretation of index r	not relevant
5.	yes	1				
6.	yes	1				
7.	yes	1				
8.	yes	1				
9.	yes	1				
10.	yes	1				
11.	yes	1				
12.	yes	1				
13. Other:	yes	1				
14. Other:	yes	1				
		13.5	14	96.42857		

Question	Answer	Points	Relevant q	Overall quality	Comments	Options
1.	yes	1				yes
2.	yes	1				partly
3.	yes	1				no/notstated
4.	yes	1				not relevant
5.	yes	1				
6.	no/notstat	0			data collection environmental variables not clear	
7.	yes	1				
8.	yes	1				
9.	yes	1				
10.	partly	0.5			Measures used for numbers in descriptive table not mentioned	
11.	no/notstat	0				
12.	yes	1				
13. Other:	yes	1				
14. Other:	yes	1				
		11.5	14	82.14286		

Question	Answer	Points	Relevant q	Overall quality	Comments	Options
1.	yes	1				yes
2.	yes	1				partly
3.	yes	1				no/notstated
4.	partly	0.5			Difference household income and	not relevant
5.	yes	1				
6.	yes	1				
7.	yes	1				
8.	yes	1				
9.	partly	0.5			Not stated which statistics were used to retrieve p-values of descriptive table	
10.	yes	1				
11.	yes	1				
12.	yes	1				
13. Other:	yes	1				
14. Other:	yes	1				
		13	14	92.85714		

Question	Answer	Points	Relevant q	Overall quality	Comments	Options
1.	yes	1				yes
2.	yes	1				partly
3.	yes	1				no/notstated
4.	yes	1				not relevant
5.	yes	1				
6.	yes	1				
7.	no/notstat	0			dissemination area	
8.	no/notstat	0				
9.	partly	0.5			direct, indirect, and total regression coefficients of spatial lag model not described	
10.	no/notstat	0			no descriptive/summary table provided	
11.	not relevar	0			ecological data	
12.	yes	1				
13. Other:	yes	1				
14. Other:	not relevar	0			publicly available data, no participants involved	
		8.5	12	70.83333		

Question	Answer	Points	Relevant q	Overall quality	Comments	Options
1.	yes	1				yes
2.	yes	1				partly
3.	yes	1				no/notstated
4.	yes	1				not relevant
5.	yes	1				
6.	no/notstat	0			SEP 1980, 1990, 2000, sports facilities 1998	
7.	yes	1				
8.	partly	0.5			Only stated: 'The 20% of subjects with missing information on income were included in an additional category'	
9.	no/notstat	0			Not clear how p values for descriptive table were retrieved	
10.	partly	0.5			p values not described	
11.	yes	1				
12.	yes	1				
13. Other:	partly	0.5			funding sources not disclosed	
14. Other:	no/notstat	0				
		9.5	14	67.85714		

Question	Answer	Points	Relevant q	Overall quality	Comments	Options
1.	yes	1				yes
2.	yes	1				partly
3.	yes	1				no/notstated
4.	yes	1				not relevant
5.	yes	1				
6.	no/notstat	0			Data collection years used for descriptives not very clear	
7.	yes	1				
8.	yes	1				
9.	yes	1				
10.	partly	0.5			Data collection years used for descriptives not very clear	
11.	partly	0.5			Response rate not specifically mentioned, however, numbers of people who provided data on HOMAR-IR and data on covariates out of total live singleton births provied	
12.	yes	1				
13. Other:	yes	1				
14. Other:	yes	1				
		12	14	85.71429		

Question	Answer	Points	Relevant q	Overall quality	Comments	Options
1.	yes	1				yes
2.	yes	1				partly
3.	no/notstat	0				no/notstated
4.	yes	1				not relevant
5.	partly	0.5			commercial recreational facilities not further described	
6.	partly	0.5			SEP collected 2007-2011, parks 2009-2012, recr facilities 2010	
7.	no/notstat	0				
8.	yes	1				
9.	yes	1				
10.	yes	1				
11.	not relevar	0			Ecological data	
12.	yes	1				
13. Other:	yes	1				
14. Other:	not relevar	0			publicly available data	
		9	12	75		

Question	Answer	Points	Relevant q	Overall quality	Comments	Options
1.	yes	1				yes
2.	yes	1				partly
3.	yes	1				no/notstated
4.	yes	1				not relevant
5.	yes	1				
6.	yes	1				
7.	no/notstat	0				
8.	no/notstat	0				
9.	yes	1				
10.	yes	1				
11.	yes	1				
12.	yes	1				
13. Other:	partly	0.5			funding not disclosed	
14. Other:	not relevan	0			publicly available data and not participants involved	
		10.5	13	80.76923		

Question	Answer	Points	Relevant q	Overall quality	Comments	Options
1.	yes	1				yes
2.	yes	1				partly
3.	yes	1				no/notstated
4.	yes	1				not relevant
5.	yes	1				
6.	no/notstat	0			Data collection years Walk Score not clear	
7.	no/notstat	0				
8.	yes	1				
9.	yes	1				
10.	yes	1				
11.	not relevar	0			ecological data	
12.	yes	1				
13. Other:	partly	0.5			funding sources not disclosed	
14. Other:	not relevar	0			publicly available data	
		9.5	12	79.16667		

Question	Answer	Points	Relevant q	Overall quality	Comments	Options
1.	yes	1				yes
2.	yes	1				partly
3.	no/notstat	0				no/notstated
4.	yes	1				not relevant
5.	yes	1				
6.	partly	0.5			2016 for income and 2014-2018 for GVI	
7.	yes	1				
8.	no/notstat	0				
9.	yes	1				
10.	yes	1				
11.	no/notstat	0				
12.	yes	1				
13. Other:	partly	0.5			conflicts of interest not disclosed	
14. Other:	no/notstat	0				
		9	14	64.28571		

Question	Answer	Points	Relevant q	Overall quality	Comments	Options
1.	yes	1				yes
2.	yes	1				partly
3.	yes	1				no/notstated
4.	yes	1				not relevant
5.	yes	1				
6.	yes	1				
7.	no/notstat	0			Dong level	
8.	no/notstat	0				
9.	partly	0.5			Not clear whether p-value for x-y relation was also tested besides the p-value for spatial variability in GWR model	
10.	yes	1				
11.	not relevar	0			ecological data	
12.	yes	1				
13. Other:	yes	1				
14. Other:	not relevar	0			Publicly available data and no participants involved	
		9.5	12	79.16667		

Kim2023a

Question	Answer	Points	Relevant q	Overall quality	Comments	Options
1.	yes	1				yes
2.	yes	1				partly
3.	yes	1				no/notstated
4.	yes	1				not relevant
5.	partly	0.5			NDVI yes, park access not described in detail.	
6.	no/notstat	0			Data collection years SEP index not clear	
7.	no/notstat	0			census tract	
8.	yes	1				
9.	yes	1				
10.	yes	1				
11.	no/notstat	0				
12.	yes	1				
13. Other:	yes	1				
14. Other:	yes	1				
		10.5	14	75		

Question	Answer	Points	Relevant q	Overall quality	Comments	Options
1.	yes	1				yes
2.	yes	1				partly
3.	no/notstat	0				no/notstated
4.	yes	1				not relevant
5.	partly	0.5			% Park not described in detail	
6.	no/notstat	0			income 2015-2019, parks 2018, NDVI not clear, water not clear	
7.	no/notstat	0				
8.	yes	1				
9.	partly	0.5			methods not repeatable with this small amount of information of % parks and % water features	
10.	yes	1				
11.	not relevar	0			ecological data	
12.	yes	1				
13. Other:	yes	1				
14. Other:	not relevar	0			publicly available data	
		8	12	66.66667		

Question	Answer	Points	Relevant q	Overall quality	Comments	Options
1.	yes	1				yes
2.	yes	1				partly
3.	yes	1				no/notstated
4.	yes	1				not relevant
5.	yes	1				
6.	no/notstat	0			years of data collection green space not clear	
7.	no/notstat	0			neighbourhood	
8.	yes	1				
9.	yes	1				
10.	yes	1				
11.	yes	1				
12.	yes	1				
13. Other:	yes	1				
14. Other:	yes	1				
		12	14	85.71429		

Question	Answer	Points	Relevant q	Overall quality	Comments	Options
1.	yes	1				yes
2.	yes	1				partly
3.	yes	1				no/notstated
4.	yes	1				not relevant
5.	yes	1				
6.	no/notstat	0			Data collection years parks not clear	
7.	no/notstat	0				
8.	partly	0.5			Only from n of regression tables is clear that missing data was removed (lower number of block groups than in descriptive table)	
9.	yes	1				
10.	yes	1				
11.	not relevar	0			ecological data	
12.	yes	1				
13. Other:	yes	1				
14. Other:	not relevar	0			publicly available data	
		9.5	12	79.16667		

Question	Answer	Points	Relevant q	Overall quality	Comments	Options
1.	yes	1				yes
2.	yes	1				partly
3.	yes	1				no/notstated
4.	yes	1				not relevant
5.	yes	1				
6.	yes	1				
7.	no/notstat	0				
8.	yes	1				
9.	yes	1				
10.	yes	1				
11.	not relevar	0			ecological data	
12.	yes	1				
13. Other:	yes	1				
14. Other:	not relevar	0			publicly available data	
		11	12	91.66667		

Question	Answer	Points	Relevant q	Overall quality	Comments	Options
1.	yes	1				yes
2.	yes	1				partly
3.	yes	1				no/notstated
4.	yes	1				not relevant
5.	yes	1				
6.	no/notstat	0			2006-2010 and 2012	
7.	no/notstat	0				
8.	no/notstat	0				
9.	no/notstat	0			statistical details on regression analyses not descibed in methods	
10.	yes	1				
11.	not relevar	0			ecological data	
12.	yes	1				
13. Other:	no/notstat	0				
14. Other:	not relevar	0			publicly available data	
		7	12	58.33333		

Question	Answer	Points	Relevant q	Overall quality	Comments	Options
1.	yes	1				yes
2.	yes	1				partly
3.	yes	1				no/notstated
4.	yes	1				not relevant
5.	yes	1				
6.	yes	1				
7.	no/notstat	0				
8.	no/notstat	0				
9.	yes	1				
10.	yes	1				
11.	not relevar	0			ecological data	
12.	yes	1				
13. Other:	yes	1				
14. Other:	not relevar	0			no participants involved and publicly available data	
		10	12	83.33333		

Question	Answer	Points	Relevant q	Overall quality	Comments	Options
1.	yes	1				yes
2.	yes	1				partly
3.	yes	1				no/notstated
4.	yes	1				not relevant
5.	yes	1				
6.	no/notstat	0			data collection years not clear	
7.	no/notstat	0				
8.	no/notstat	0				
9.	yes	1				
10.	yes	1				
11.	not relevar	0			ecological data	
12.	yes	1				
13. Other:	yes	1				
14. Other:	not relevar	0			public data, no participants involved	
		9	12	75		

Question	Answer	Points	Relevant q	Overall quality	Comments	Options
1.	yes	1				yes
2.	yes	1				partly
3.	yes	1				no/notstated
4.	yes	1				not relevant
5.	yes	1				
6.	no/notstat	0			Data collection years of SEP and some exposure variables not clear	
7.	no/notstat	0				
8.	no/notstat	0				
9.	yes	1				
10.	yes	1				
11.	not relevar	0			ecological data	
12.	yes	1				
13. Other:	yes	1				
14. Other:	not relevar	0			publicly available and register data	
		9	12	75		

Question	Answer	Points	Relevant q	Overall quality	Comments	Options
1.	yes	1				yes
2.	yes	1				partly
3.	yes	1				no/notstated
4.	yes	1				not relevant
5.	yes	1				
6.	no/notstat	0				
7.	partly	0.5				
8.	yes	1				
9.	yes	1				
10.	yes	1				
11.	no/notstat	0				
12.	yes	1				
13. Other:	yes	1				
14. Other:	yes	1				
		11.5	14	82.14286		

Question	Answer	Points	Relevant q	Overall quality	Comments	Options
1.	yes	1				yes
2.	yes	1				partly
3.	yes	1				no/notstated
4.	partly	0.5				not relevant
5.	yes	1				
6.	yes	1				
7.	no/notstat	0				
8.	no/notstat	0				
9.	yes	1				
10.	yes	1				
11.	not relevar	0				
12.	no/notstat	0				
13. Other:	partly	0.5				
14. Other:	not relevar	0				
		8	12	66.66667		

Question	Answer	Points	Relevant q	Overall quality	Comments	Options
1.	yes	1				yes
2.	yes	1				partly
3.	yes	1				no/notstated
4.	yes	1				not relevant
5.	yes	1				
6.	yes	1				
7.	partly	0.5				
8.	yes	1				
9.	yes	1				
10.	yes	1				
11.	no/notstat	0				
12.	yes	1				
13. Other:	partly	0.5				
14. Other:	yes	1				
		12	14	85.71429		
