# Supplementary material for: Socioeconomic inequalities in exposure to neighbourhood environments for physical activity: a systematic review
Source: Int J Behav Nutr Phys Act. 2026 Apr 9;23:58. doi: 10.1186/s12966-026-01912-1 (PMC13231669; doi:10.1186/s12966-026-01912-1)
Supplement: Supplementary file 8 — Supplementary Material 8. [file 12966_2026_1912_MOESM8_ESM.pdf]

Figure S2

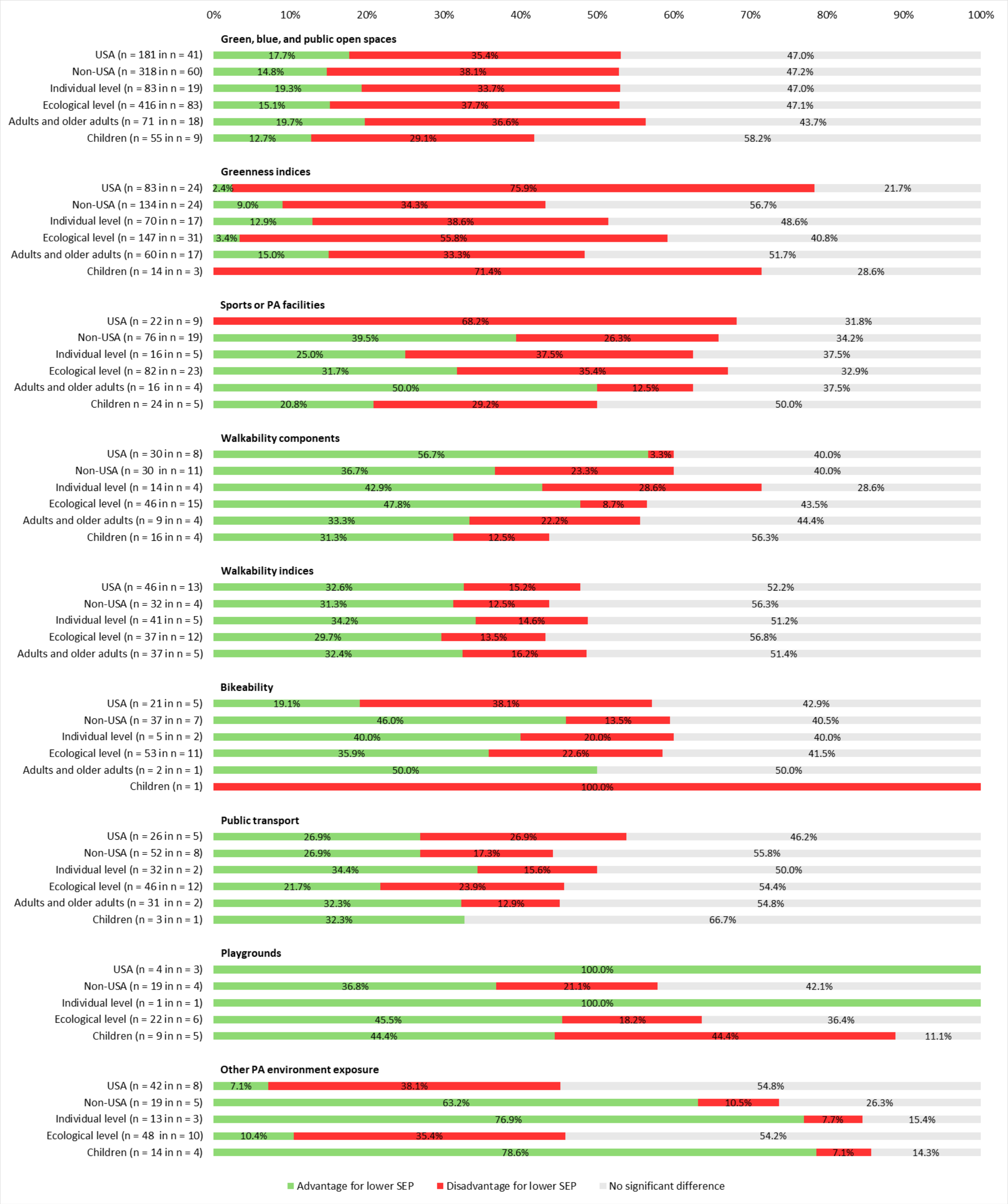

Associations between SEP and PA environment exposure by USA/non-USA, unit of analysis, and age group. Note: For these additional analyses, regression and non-regression results, as well as all SEP measures, were combined. SEP: Socioeconomic position; PA: Physical activity; notation ‘n = … in n = …’ indicates the number of associations (first n) and the number of studies from which these associations were derived (second n).

Figure S3

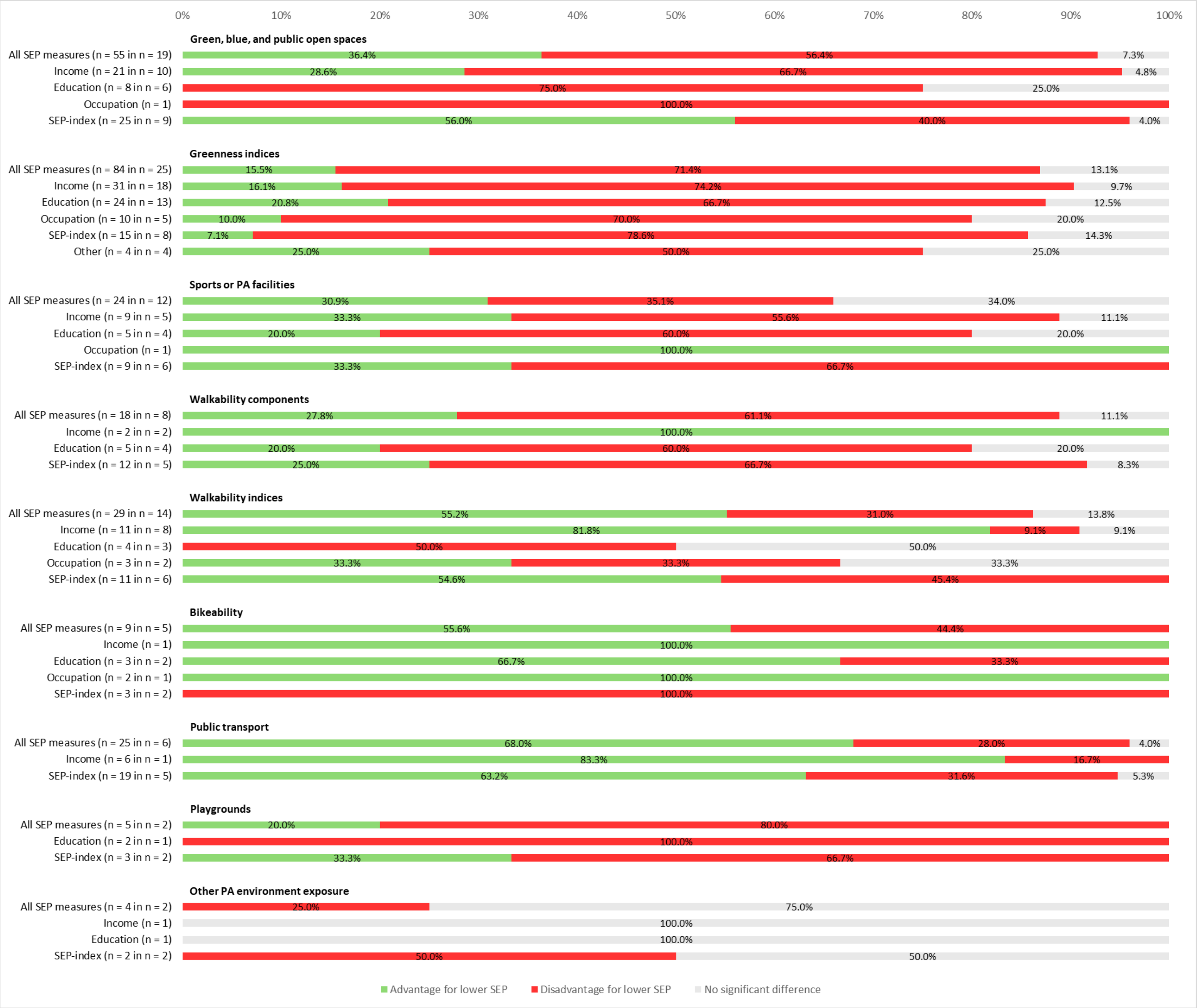

Results of descriptive statistics for SEP and PA environment exposure by PA resource and SEP proxy. SEP: Socioeconomic position; PA: Physical activity; notation ‘n = … in n = …’ indicates the number of associations (first n) and the number of studies from which these associations were derived (second n).
