# Supplementary material for: Socioeconomic inequalities in exposure to neighbourhood environments for physical activity: a systematic review
Source: Int J Behav Nutr Phys Act. 2026 Apr 9;23:58. doi: 10.1186/s12966-026-01912-1 (PMC13231669; doi:10.1186/s12966-026-01912-1)
Supplement: Supplementary file 9 — Supplementary Material 9. [file 12966_2026_1912_MOESM9_ESM.pdf]

Supplementary File 8: Robustness checks excluding high-volume studies

To assess whether studies reporting the largest number of associations disproportionately influenced the aggregated findings, we conducted robustness checks for all PA resource categories. For each category, we removed the study contributing the largest number of associations and recalculated the distribution of advantage, disadvantage, and no statistically significant difference findings.

<u>**Regression associations**</u>

Greenness indices

- Before excluding Quinton et al., 2022[1]: 138 associations; 6.5% advantage, 38.4% disadvantage, 55.1% no statistically significant difference
- After excluding Quinton et al., 2022[1]: 76 associations; 7.9% advantage, 51.3% disadvantage, 40.8% no statistically significant difference
- Pattern: disadvantage increased; no statistically significant difference decreased.

Bikeability

- Before excluding Houde et al., 2018[2]: 45 associations; 42.2% advantage, 22.2% disadvantage, 35.6% no statistically significant difference
- After excluding Houde et al., 2018[2]: 21 associations; 23.8% advantage, 47.6% disadvantage, 28.6% no statistically significant difference
- Pattern: shifted from advantage-dominant to disadvantage-dominant.

Walkability indices

- Before excluding Robinson et al., 2018[3]: 57 associations; 29.8% advantage, 14.0% disadvantage, 56.1% no statistically significant difference
- After excluding Robinson et al., 2018[3]: 28 associations; 32.1% advantage, 14.3% disadvantage, 53.6% no statistically significant difference
- Pattern: stable.

Playgrounds

- Before excluding Schneider et al., 2019[4]: 19 associations; 42.1% advantage, 21.1% disadvantage, 36.8% no statistically significant difference
- After excluding Schneider et al., 2019[4]: 7 associations; 100% advantage
- Pattern: all remaining associations showed advantage.

Public transport

- Before excluding Robinson et al., 2018[3]: 52 associations; 30.8% advantage, 25.0% disadvantage, 44.2% no statistically significant difference
- After excluding Robinson et al., 2018[3]: 23 associations; 26.1% advantage, 39.1% disadvantage, 34.8% no statistically significant difference

- Pattern: disadvantage increased; no statistically significant difference decreased.

Other PA facilitators

- Before excluding Rodgers et al., 2012[5]: 40 associations; 35.0% advantage, 20.0% disadvantage, 45.0% no statistically significant difference
- After excluding Rodgers et al., 2012[5]: 29 associations; 13.8% advantage, 27.6% disadvantage, 58.6% no statistically significant difference
- Pattern: advantage decreased; no statistically significant difference increased.

<u>**Non-regression associations**</u>

Green, blue and public open spaces

- Before excluding Aamodt et al., 2023[6]: 291 associations; 15.8% advantage, 34.7% disadvantage, 49.5% no statistically significant difference
- After excluding Aamodt et al., 2023[6]: 213 associations; 17.8% advantage, 44.1% disadvantage, 38.0% no statistically significant difference
- Pattern: disadvantage increased; no statistically significant difference decreased.

Sports facilities

- Before excluding Schneider et al., 2015[7]: 61 associations; 39.3% advantage, 26.2% disadvantage, 34.4% no statistically significant difference
- After removing Schneider et al., 2015[7]: 46 associations; 19.6% advantage, 34.8% disadvantage, 45.7% no statistically significant difference
- Pattern: advantage decreased; disadvantage and no statistically significant difference increased

Walkability components

- Before excluding Carroll et al., 2023[8]: 35 associations; 45.7% advantage, 17.1% disadvantage, 37.1% no statistically significant difference
- After excluding Carroll et al., 2023[8]: 27 associations; 44.4% advantage, 11.1% disadvantage, 44.4% no statistically significant difference
- Pattern: largely stable

References

1. Quinton J, Nesbitt L, Czekajlo A. Wealthy, educated, and ... non-millennial? Variable patterns of distributional inequity in 31 Canadian cities. LANDSCAPE AND URBAN PLANNING. 2022;227.
2. Houde M, Apparicio P, Séguin AM. A ride for whom: Has cycling network expansion reduced inequities in accessibility in Montreal, Canada? JOURNAL OF TRANSPORT GEOGRAPHY. 2018;68:9–21.
3. Robinson O, Tamayo I, de Castro M, Valentin A, Giorgis-Allemand L, Hjertager Krog N, et al. The Urban Exposome during Pregnancy and Its Socioeconomic Determinants. Environ Health Perspect. 2018;126(7):077005.
4. Schneider S, Bolbos A, Fessler J, Buck C. Deprivation amplification due to structural disadvantage? Playgrounds as important physical activity resources for children and adolescents. Public health. 2019;168:117–27.
5. Rodgers SE, Demmler JC, Dsilva R, Lyons RA. Protecting health data privacy while using residence-based environment and demographic data. Health & Place. 2012;18(2):209–17.
6. Aamodt G, Nordh H, Nordbo ECA. Relationships between socio-demographic/socio-economic characteristics and neighborhood green space in four Nordic municipalities-results from NORDGREEN. URBAN FORESTRY & URBAN GREENING. 2023;82.
7. Schneider S, D'Agostino A, Weyers S, Diehl K, Gruber J. Neighborhood Deprivation and Physical Activity Facilities - No Support for the Deprivation Amplification Hypothesis. Journal of physical activity & health. 2015;12(7):990–7.
8. Carroll SJ, Dale MJ, Turrell G. Neighbourhood socioeconomic disadvantage and body size in Australia's capital cities: The contribution of obesogenic environments. PLoS One. 2023;18(1):e0280223.
